# “Figuring stuff out myself” – a qualitative study on maternal vaccination in socially and ethnically diverse areas in England

**DOI:** 10.1186/s12889-023-16317-z

**Published:** 2023-07-21

**Authors:** Sima Berendes, Sandra Mounier-Jack, Oyinkansola Ojo-Aromokudu, Alice Ivory, Joseph D. Tucker, Heidi J. Larson, Caroline Free

**Affiliations:** 1 Department of Population Health, London School of Hygiene and Tropical Medicine, London, UK; 2 Department of Global Health and Development, London School of Hygiene and Tropical Medicine, London, UK; 3 Department of Clinical Research, London School of Hygiene and Tropical Medicine, London, UK; 4 Department of Medicine, University of North Carolina, Chapel Hill, NC USA; 5 Department of Infectious Disease Epidemiology, London School of Hygiene and Tropical Medicine, London, UK; 6 Department of Health Metrics Sciences, University of Washington, Seattle, WA USA

**Keywords:** Public health, Health services, Maternal vaccination, Influenza, Pertussis, Covid-19, Antenatal care, Vaccine confidence, Pregnancy, Healthcare providers

## Abstract

**Background:**

Maternal vaccinations against Influenza, Pertussis, and Covid-19 are recommended in the UK, and vaccines against further infections may become available soon. However, many pregnant women, especially in socially and ethnically diverse areas, have low vaccine uptake. Qualitative studies on the reasons and possible solutions are needed that are inclusive of disadvantaged and minority ethnic groups. We therefore aimed to understand the complex interplay between structural and behavioural factors contributing to the low maternal vaccine uptake in socially and ethnically diverse areas in London in the Covid-19 context.

**Methods:**

In 2022, we conducted semi-structured interviews and a focus group discussion among a purposive sample of 38 pregnant/recently pregnant women and 20 health service providers, including 12 midwives. Participants were recruited in ethnically diverse London boroughs. We followed a critical realist paradigm and used a thematic analysis approach.

**Results:**

The sample included participants who took all, some or none of the maternal vaccines, with some participants unsure whether they had taken or been offered the vaccines. Decision-making was passive or active, with the expectation for pregnant women to do their 'own research'. Participants described various individual, social and contextual influences on their decision-making as they navigated the antenatal care system. Missing or conflicting information from providers meant knowledge gaps were sometimes filled with misinformation from unreliable sources that increased uncertainties and mistrust. Both pregnant women and providers described structural and organisational factors that hindered access to information and vaccinations, including lack of training, time and resources, and shortcomings of health information systems and apps. Some participants described factors that facilitated vaccination uptake and many made recommendations for improvements.

**Conclusions:**

Our study showed how structural and organisational factors can compound uncertainties around maternal vaccination among socially and ethnically diverse populations. Results highlight the need for more reliable resources, streamlined workflows, improved electronic information systems and training in their use. Roles and responsibilities should be clarified with potential greater involvement of nurses and pharmacists in vaccine provision. Education and communication should consider individual (language/digital) skills and needs for information and reassurance. Further research is needed to co-produce solutions with service users and providers.

**Supplementary Information:**

The online version contains supplementary material available at 10.1186/s12889-023-16317-z.

## Introduction

Pregnant women and their newborns are particularly susceptible to the effects of influenza, pertussis, and Covid-19, and maternal vaccinations against these infections have been recommended in the United Kingdom (UK) since 2010, 2012, and 2021 respectively [1–3]. Maternal vaccinations are dually beneficial, as they protect both mother and baby via transplacental antibodies. Vaccinations can not only significantly reduce infection-related morbidity, mortality and costs, but also indirectly reduce the risk of antimicrobial drug resistance through reduction of (secondary) bacterial infection and unnecessary prescribing of antibiotics for viral infections [4–9].

Recent successful trials of maternal vaccines against Respiratory Syncytial Virus (RSV) mean that these will most likely become available soon [10, 11]. Further maternal vaccines, including against Group B Streptococcus, are currently under development [12, 13].

However, maternal vaccination uptake has been far below national targets and unevenly distributed across ethnic groups and regions. Pregnant women in London and from minority ethnic groups have particularly low vaccination rates [14–17]. For example, in 2021–2022 the maternal influenza vaccine coverage among women of Black Caribbean background in London was only 11%, well below the average for London (30%) and far below the national target (75%) [14]. A recent decline in maternal pertussis vaccine coverage in England (2019: 69%, 2022: 61%), has largely been driven by a decrease in London (2019: 57%, 2022: 39%) [16]. Similarly, Covid-19 vaccine uptake has been lower in London, in socio-economically deprived areas and among some minority ethnic groups [15, 18]. For example, a survey at an antenatal/maternity outpatient clinic in London in October/November 2021 showed that 88% of pregnant women from mixed/multiple ethnicity, 73% of black, 59% of white/other white and 31% of Asian ethnicity had been unvaccinated against Covid-19 [19].

Qualitative research on the low maternal vaccine uptake and possible solutions are limited, focus mostly on behavioural factors, were either conducted before Covid-19 vaccinations became available to pregnant women or did not include in-depth accounts from minority ethnic groups and from both service providers and users. A recent systematic review and meta-analysis included mostly quantitative studies showing that healthcare professional (HCP) recommendation, and vaccine safety and efficacy beliefs were main factors influencing vaccination decision-making [20]. Additional factors revealed by qualitative studies related to personal sentiments, rumours, trust and cultural values. Overall, of 120 included studies, four were from the UK, including one qualitative study (from Northern Ireland) [21]. Since then, only few qualitative studies were published, including one conducted in East-London in 2015/2016 [22]. Other more recent qualitative studies included only few participants from minority ethnic groups and were conducted before maternal Covid-19 vaccines became available [23–25].

Qualitative research is particularly well-suited to understand the interplay of individual, social and contextual factors that may influence vaccine uptake and to explore issues in-depth. For example, we know from previous international studies that lack of provider recommendation is an important barrier to vaccine uptake, but we do not know why providers, especially midwives, do not make unambiguous recommendations in certain contexts [20, 26–28]. The reasons are likely complex.

We therefore conducted a qualitative study to understand the complex interplay between structural and behavioural factors contributing to low maternal vaccine uptake in socially and ethnically diverse areas in London during the Covid-19 pandemic.

## Methods

### Qualitative approach and theory

As part of the VIP-IDEAL (Vaccination in pregnancy—ideas, experiences and attitudes) study, we conducted semi-structured interviews and a focus group discussion to obtain rich and detailed information on factors relevant to the uptake of vaccinations in pregnancy. All research team members have training and experience in qualitative research and expertise in the areas of sexual and reproductive health, vaccine confidence or health services research. The lead researcher kept a reflective journal throughout the research process and maintained self-reflexivity, including on her positionality, not only during field work, but also during analysis, aiming to avoid imposing own assumptions and pre-defined theories onto participants’ narratives [29].

Study design and implementation were guided by a critical realist paradigm [30, 31] and informed by discussions with patient and public involvement (PPI) contributors and a conceptual framework developed based on existing theory and research [20, 22, 32–39], and adapted during analysis (details in Supplementary file 1). We followed Standards for Reporting Qualitative Research guidance [40] (Supplementary file 2).

### Study population and setting

We conducted the study among a purposive sample of pregnant and post-partum (i.e. recently given birth) women and health service providers in South London. We focused on those South London boroughs (Southwark, Lambeth, Lewisham and Croydon) and areas within these boroughs with populations who, based on Index of Multiple Deprivation (IMD) and ethnicity, were less likely to accept or access maternal vaccinations [14–17, 24, 41].

Pregnant/postpartum women were eligible if they were at least 16 years old, lived and/or received healthcare in South London and were pregnant or had given birth to a baby any time after April 2021 (when pregnant women in the UK had been advised to get vaccinated against Covid-19 at the same time as the general population). Healthcare providers or other relevant stakeholders were eligible, who regularly provided services to pregnant/post-partum women and/or might have an influence on the current or future uptake of maternal vaccinations in South London.

With support from the National Institute for Health and Care Research Clinical Research Network (NIHR CRN) and PPI contributors we recruited participants via clinics and within the community (e.g., via parent–child groups, social media and posters/flyers, details in Supplementary file 1). We screened 62 pregnant/post-partum women, among whom 55 were eligible, 47 willing to participate, and 38 were recruited (five pregnant and 33 post-partum women). We had planned to conduct 20–25 interviews with pregnant/postpartum women (including based on factors described by Malterud et al. [42]) and 1–3 FGDs for further insight. However, we completed 31 interviews and only one FGD (with seven participants) due to scheduling challenges, most women’s preference for interviews and as data saturation for many important themes relevant to our research question had been reached (based on concurrent data analysis, reflective notes and team discussions).

All healthcare providers that had responded to our recruitment efforts via email were eligible and participated, including 12 midwives, one maternal support worker, and two GPs. We stopped recruitment of midwives after only little new information was generated from further interviews, and recruitment of GPs after it emerged during interviews and PPI work that they played a comparably minor role only in maternal vaccinations in London. For additional insight we also spoke to nine other service providers, among whom two pharmacists, one pharmacy manager/vaccinator, one public health specialist, one community engagement and one digital health data specialist agreed to participate in semi-structured interviews.

### Data collection and analysis

According to participants’ preferences, interviews were conducted either face-to-face or via video/telephone call and lasted 22–93 min. The FGD was held via video call (130 min). All participants received £20 as a token of appreciation. Translations/interpretation was available to participants if needed and mothers were allowed to bring babies/older siblings to the interviews/FGD (details in Supplementary file 1).

One researcher (SB) obtained informed consent and completed all interviews and two researchers (SB & OOA, both female researchers not involved in clinical NHS work) facilitated the FGD following semi-structured topic guides. These explored maternal vaccine-related experiences, information sources, knowledge, attitudes, beliefs, behaviour, recommendations for improvements and factors that (may) enable or impede current and future vaccine uptake and intervention development (Supplementary file 3). The interviews and FGD were audio recorded, transcribed verbatim, anonymized, stored, and coded using a qualitative data analysis software (NVivo12). Part of the data were duplicate-coded by OOA and AI. We followed the six steps of thematic analysis (in an iterative way and alongside data collection), including 1. familiarising ourselves with the data, 2. generating initial codes, 3. searching for themes, 5. defining and naming themes, and 6. producing the report [43]. We also followed guidance by Nowell et al. (2017 [44]) to establish trustworthiness during each phase of thematic analysis, including through peer debriefing, reflexive journaling, documentation of reflective thoughts and documentation of team meetings (details in Supplementary file 1).

## Results

Between April and September 2022 a total of 38 pregnant/postpartum women (pregnant during the Covid-19 pandemic with a delivery/due date after Covid-19 vaccines) and 20 health service providers, including 12 midwives, participated in the study. Participant characteristics are provided in Table 1.

**Table 1:** Characteristics of qualitative research participants, South London, 2022 (total *N* = 58)

Characteristic	Pregnant /Post-partum women^a^		Service providers
	Interviewees, n (*N* = 31)	FGD particip., n (*N* = 7)	Interviewees, n (*N* = 20)
**Age**^**b**^			
< 30 years	5	0	7
30 – 40 years	24	6	4
> 40 years	2	1	9
**Gender**^**c**^			
Female	31	7	18
Male	0	0	2
**Ethnic group**			
Asian/Asian British	2	0	2
Black/Black British—Afr./ Carib	15	0	4
White British	2	4	8
Other white	5	2	4
Mixed/Multiple/Other	7	1	2
**IMD decile**			
1–2 (most deprived)	7	1	-
3–4	12	1	-
5–6	1	1	-
7–8	1	0	-
9–10 (least deprived)	0	0	-
No data/ no full post code given	10	4	-
**Borough in South London**			
Southwark	14	5	-
Lambeth	8	2	-
Lewisham	4	0	
Other^**d**^	5	0	-
**Religion**			
Christian	17	-	7
Muslim	6	-	0
None/ Agnostic/ Atheist	7	-	10
Other	1	-	3
**Marital status**			
Married/ in partnership	22	-	-
Not married/ in partnership	9	-	-
**Highest level of education**			
Primary or secondary school	5	-	0
College	4	-	1
Undergraduate degree (e.g. BA)	11	-	12
Postgraduate (e.g. MSc, Dipl.)	11	-	7
**Employment**			
Yes, full-time	17	-	16
Yes, part-time	4	-	4
No	10	-	0
**Profession**			
Midwife	-	-	12
GP^**e**^	-	-	2
Pharmacist/Pharmacy manager	-	-	3
Other^**f**^	-	-	4
**Delivery/Due date**^**g**^			
Spring/Summer 21 (May-Aug21)	7	0	-
Autumn 21 ( Sep-Nov21)	4	1	-
Winter 21/22 (Dec21-Feb22)	6	3	-
Spring 22 (Mar-May22)	8	1	-
Summer 22 (Jun-Aug22)	5	2	-
Autumn 22 (Sep-Nov22)	2	0	-
**Older child/ previous life birth(s)**			
Yes	12	2	-
**Vaccination in pregnancy**			
Took/ plans to take all vaccines	9	5	-
Took/ plans to take some vaccines	14	2	-
Took/ plans to take no vaccines	8	0	-

*FGD particip* Focus group discussion participants, *IMD* 2019 Index of Multiple Deprivation [45]

^a^*n* = 4 pregnant and *n* = 28 post-partum interviewees (one interviewee was pregnant *and* post-partum); *n* = 1 pregnant and *n* = 6 post-partum FGD participants

^b^Age: Mean age of pregnant/post-partum interviewees: 33.7 (range 21–49); Mean age of providers: 38.8 (range 24–59)

^c^Gender: No participant with non-binary/other gender

^**d**^Borough of London-other: Croydon (*n* = 2), Bexley (*n* = 1), Sutton (*n* = 1), Wandsworth (*n* = 1)

^e^Profession-GP: Data do not add up to 20, as one of the GPs was also a post-partum woman and counted among these

^**f**^Profession-other: 1 community engagement officer, 1 digital health specialist, 1 maternity support worker, 1 public health specialist

^g^Delivery/Due date: Data add up to *N* = 32, instead of *N* = 31, as one interviewee was ‘double eligible’, i.e. pregnant *and* post-partum

The sample purposively included those living in more deprived areas (according to IMD-2019 [45]) and those from Black/ Black British, non-British white, mixed and other ethnic groups. Pregnant/post-partum women took all, some or none of the maternal vaccines with some unsure whether they had taken or been offered the vaccines. Among those participants who accepted only some vaccines, most opted for the pertussis and some for the flu vaccine, but declined the Covid-19 vaccine (Table 2).

**Table 2:** Maternal vaccine uptake among pregnant/post-partum interview and FGD participants (*n*, Total *N* = 38)

Maternal vaccine uptake	Pertussis vaccine	Influenza vaccine	Covid-19 vaccine
Yes	27	19	9
Yes, but with delay	2	2	4
No, but offered	3	5	19
No, not offered (despite indicated/ in season)	1	1	1
No (unsure if offered/ other)	4	7	1
No, not required, as already taken before pregnancy	n/a	3	2
No, not yet available (for season or age group)	n/a	0	2
Unsure	1	1	0

While most providers interviewed were proponents of maternal vaccination, some were hesitant towards one or all of the maternal vaccines and most opposed mandatory Covid-19 vaccination of healthcare professionals (HCP).

An overview of the main themes identified is provided in Table 3, and these are further described below with exemplary anonymised quotes. (When stating ‘participant’ in the narrative, we mean participants from both groups; when referring to participants from the provider group only, we say for example ‘midwife’ or ‘HCP’. By ‘women’ or ‘mothers’ we mean pregnant/post-partum participants; acronyms after quotes: W = pregnant/post-partum woman; M = Midwife, O = Other provider). Further quotes illustrating (sub-) themes can be found in Supplementary file 4.

**Table 3:** Overview of categories and (sub-)themes, VIP-IDEAL study, 2022

Overarching Categories	Categories	(Sub-)Themes^a^
Structural and organisational factors	Organisation of and access to ANC	Limited GP involvement due to new midwife-led ANC services and access via new online self-referral system (and related confusion); delayed access/ disrupted ANC; organisational challenges and changes due to the Covid-19 pandemic (including remote ANC visits and suspension of continuity of care system)
	ANC booking visit	Lack of time and limited vaccination-related information
	Subsequent ANC visits	Lack of time and lack of vaccination-related information and reminders
	Access to influenza and pertussis vaccines within maternity and at GP clinics	Access to influenza and pertussis vaccines within maternity; access to influenza and pertussis vaccines via GP versus maternity and recent changes; opportunistic vaccination
	Access to Covid vaccines within maternity and via vaccination centres	Temporary access to Covid vaccines in maternity in hospital; Access to Covid-vaccines at vaccination centres (including lack of special arrangement for pregnant women and reduced trust/confusion about change of guidance)
	Access to maternal vaccines via pharmacies	Access to maternal vaccines via pharmacies (including advantages and barriers)
	Resources, roles and responsibilities	Resource challenges in maternity services (including lack of staffing, space and supply) and in pharmacies; vaccination-related roles and responsibilities of different providers
	Health information system and Apps	Insufficient information transfer between providers and shortcomings of electronic health records, referral systems and documentation; lack of user-friendliness of apps and not used to access vaccine information; information for participants via app versus hardcopy material (including digital exclusion and language barriers)
Behavioural factors	Passive versus active decision-making process	Passive decision-making; active decision-making (with own research)
	Interaction with HCP/ Provider recommendations	Recommendation against Covid-19 vaccines or no clear recommendation or information; no clear recommendation/ no information/dialogue regarding pertussis and influenza vaccines; importance of not putting pressure on women, and vaccination as personal decision; unambiguous recommendation as facilitator of vaccine uptake
	Engagement with information material and social media	Hardcopy or electronic leaflets; Searching online/ via social media (including to fill information gaps and to search for personal stories from others)
	Interaction with family, friends and others	Interaction with and influence of mothers, partners and others (including personal stories); interest in decisions of other pregnant women (including in waiting rooms)
	Individual characteristics and influences on vaccination decisions:	
	Risk–benefit perceptions	Risk benefit evaluation and motivation to accept/recommend vaccines (including based on own/ others previous experience of vaccine/vaccine-preventable disease); perceived risk and benefit of vaccine/vaccine-preventable disease on baby and/or/versus mother; perception of pregnancy as vulnerability; perceived susceptibility/ risks in case of underlying health conditions or complications; perceived risk and individual biological differences; risk evaluation based on exposure; alternative risk-mitigating behaviour
	Knowledge and skills	Conflicting information; misconceptions and general health knowledge; lack of awareness or information about vaccines; ability/ opportunity to ask HCPs questions; lack of HCP training, knowledge or specific information to pass on to clients; commitment of some HCP to relay information and have honest dialogue; lack of research evidence; language and digital skills
	Emotions and trust	Fear of vaccine and/or vaccine-preventable disease; trust in healthcare system and HCP; reduced trust and/or confusion due to change of guidance; lack of trust and historical events; lack of trust and ethnicity; lack of trust and conspiracy theories/ misinformation; emotions and trust linked to pressure and mandatory vaccines
	Cultural norms, philosophy and beliefs	Social and cultural norms, including in country of origin/ abroad; religion and Covid-19 vaccination; other beliefs and philosophy (including letting nature take its course, preference for natural products and homeopathy); attitudes regarding altruistic reasons for vaccination
	Attitude towards mandatory Covid-19 vaccines	Vaccination needed for travel or work; planned mandatory vaccines for HCP; perceived pressure/ no pressure to get vaccinated
Participant recommendations	(Relating to various categories above)	Clearer provider recommendation, vaccination dialogue and more information; vaccination programmes and messages more targeted to pregnant women, and more personalised; provider training; organisational changes (including opt-out versus opt-in approach); increased accessibility; improved health information systems and apps; Provision of hardcopy information material to avoid digital exclusion; addressing language barriers

^a^Overarching categories and categories correlate with headings and sub-headings in the results section; The (sub-)themes or explanations that are placed in the third column are described/referred to in the text with additional anonymised exemplary quotes in supplementary file 4; *ANC* Antenatal care, *HCP* Health care professional

**Fig.1:**
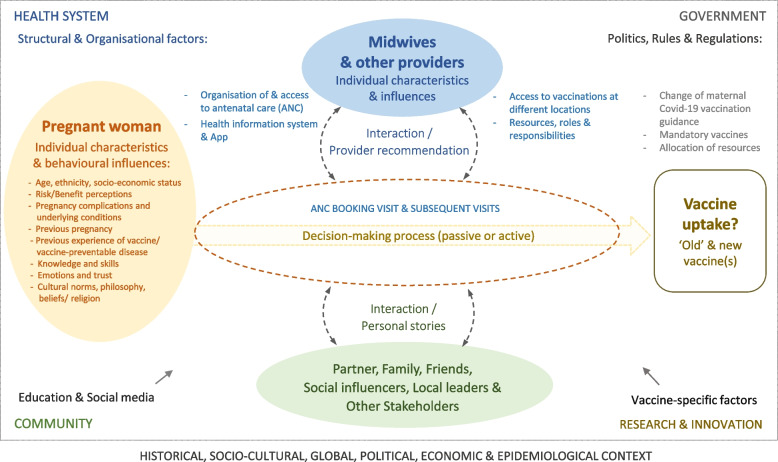
Conceptional framework of factors influencing maternal vaccine uptake based on literature and VIP-IDEAL results, 2022

Figure 1 was developed based on previous literature [20, 22, 32–39] and our results to visualise the relationships between these themes.

### Structural and organisational factors

Participants explained how ANC and the provision of maternal vaccination had been organized, which challenges they encountered and what changes had recently been made, including in response to the Covid-19 pandemic.

#### Organisation of and access to ANC

In most South London Boroughs included in the study, ANC was reportedly midwife-led and GPs played a relatively minor role. Only one HCP mentioned that in one of the boroughs, GPs still see pregnant women for two of about ten ANC visits. In other boroughs, GPs reportedly often only learned about a woman’s pregnancy after the baby was born, given that pregnant women now had to self-refer for their initial midwife visit by completing an online form. While some participants found this straightforward, many still first contacted their GP clinics, and reported confusions and delays in accessing ANC.

All pregnant women can freely access ANC in the UK’s National Health System (NHS). One immigrant from South America, however, initially did not know that she could do so without having ‘her papers’. A young Black African participant was repeatedly moved from council to council for temporary social housing, which disrupted her ANC.

During the pandemic, some of the ANC visits were temporarily held remotely (phone/online), while others still took place in-person, usually either at hospitals or within the community at child health centres. Due to the pandemic, the ‘continuity of carer’ system (support by the same team of 4–6 midwives throughout pregnancy), that had reportedly been available in some areas to socially vulnerable women, had to be interrupted. Some women found it frustrating to always have different midwives, who often asked same questions, if previous discussions had not been documented.

#### ANC booking visit

Most midwives said that during the booking visit (at 8–10 weeks pregnancy) they usually only briefly (e.g. for about*’20 s’*) mentioned vaccines due to lack of time, and as women would ‘forget about it’ due to the amount of other information conveyed at booking. Systems seemed to differ by borough, but overall few women mentioned vaccine-related hardcopy or online information material provided at booking, and some said that vaccines had not been mentioned at all. Many women, however, would have liked more information at this, or even earlier stages, including on how exactly they could access the vaccines.

“I felt like it had to be a very proactive experience of me figuring stuff out myself. There was no like, here’s an information pack, at your first midwife appointment…” (W).

One participant recounted that the midwife did not remove from the ANC notes provided at booking a ‘*big A4 piece of paper’* warning that Covid vaccines were ‘*not safe in pregnancy’*, although the guidance had already changed.

#### Subsequent ANC visits

Many midwives said they reminded clients about one or all vaccines at one or more subsequent visits, but according to mothers this was not always the case.

“almost at the end of the pregnancy they were asking me, oh have you had your whooping cough vaccine? I was like, I don’t, I didn’t remember, I didn’t know I was meant to have it, you didn’t tell me when and where to have it, you know, yeah so I didn’t have that vaccine.” (W).

A few midwives explained that there was often no time to remind clients or that reminders were not needed, as there were posters up at the hospital.

#### Access to influenza and pertussis vaccines within maternity and at GP clinics

Influenza and pertussis vaccines could reportedly be accessed at GP clinics, but increasingly also within maternity at newly introduced vaccination clinics at hospitals. While some women found access to vaccines given by nurses at close-by GP clinics easier, a few others mentioned problems of getting appointments there. This and/or temporary closures of GP clinics during lockdowns reportedly led to the introduction of (increased opening hours of) maternity vaccination clinics at hospitals. One midwife regretted that in their hospital they had meanwhile reverted to the old system with only limited opening times, although the system had been ‘*very popular and was working very well’*. Some women said they found the system convenient, as they could walk to the vaccination clinic directly after their week 20 scan – provided they had been told about it previously and/or were reminded by their midwives. In a few instances sonographers reportedly pointed women to the vaccination clinic, and some vaccination clinic staff seemed to make women aware of the clinic in the waiting area, but this was inconsistent.

“And whoever’s doing the Vaccine Clinic[…] I always come out like “anyone here waiting for vaccine”, so I just sell it like tomatoes but… some midwives are more shy and they just wait in the room.” (M).

One midwife mentioned that at their hospital vaccination clinic they had changed from an appointment to a walk-in system. Another clinic reportedly offered both options, but a few women wished opening times were ‘*more accessible around work*’.

GPs were reportedly encouraged by the Government to provide ‘opportunistic’ vaccinations (when patients attended for other reasons) during the flu season, but the interviewed GPs mentioned that time restrictions made additional vaccination discussions with pregnant women challenging. Flu vaccine invitations/reminders could also only be sent to other eligible patients, because pregnancy was no longer routinely coded in their system.

#### Access to Covid vaccines within maternity services and via vaccination centres

In a few boroughs, midwives mentioned pilot trials of offering Covid-19 vaccines within maternity (in a separate room) in their hospital, which ‘*didn’t really work’* partly for logistical reasons and ‘*uptake wasn’t that great*’. One midwife also criticized the way decision-makers had ‘*imposed*’ the Covid vaccination clinic without thinking ‘*about what was in place before*’. This also linked to issues around competition for space and resources mentioned by a few HCPs.

Midwives therefore reportedly reverted back to the old system of referring pregnant women to the general Covid-19 vaccination centre within the same hospitals. A few women, however, complained about long waiting times at such vaccination centres.

Another problem, mentioned by a vaccinator at such a centre, was that women became suspicious when they were asked at arrival ‘*are you pregnant?*’, although this was designed to monitor vaccine uptake. Another interviewee said that upon arrival at a vaccination centre her pregnant friends ‘*got asked are you pregnant? They then were told oh wait, we need to read this information’* and these reactions were ‘*enough to put them off*’. This linked to the theme of ‘reduced trust and/or confusion about the change of guidance’, including among HCPs, with a few women saying ‘*the initial advice was don’t get pregnant for three months after your vaccine*’, which ‘*stuck*’ with them.

A few providers recounted that they had rejected the idea to also offer maternal Covid-19 vaccines at their child health centre, because they felt access to vaccinations was not a problem, or women were already ‘*absolutely bombarded*’ about vaccination and they feared losing the established trust of the ‘*very deprived and ethnically diverse and historically distrusting population*’ they served.

#### Access to maternal vaccines via pharmacies

As a few women had accessed flu and/or Covid vaccines via pharmacies, we conducted additional interviews with staff at three pharmacies that offered maternal flu and/or Covid vaccines. They thought that maternal vaccines should be increasingly offered at pharmacies. One pharmacist for example who had worked within his community for a long time and enjoyed peoples’ trust, said that pregnant women often took his advice and accepted his (opportunistic) offer of freely available flu vaccines. He said he lacked the staff and space, however, for offering also Covid-19 vaccinations. Another pharmacist, who had recently modernised and offered Covid-19 vaccines to 300–400 people a day at the height of the pandemic, was convinced that other pharmacists would make the necessary investments, too, if they were given a contract that guaranteed they would then be allowed to provide the service for the NHS. He was adamant that all vaccinations should be shifted from GPs to pharmacies, as it would be ‘*a waste of time for someone to go to the GP to get the vaccine done when it can be done through the pharmacy’.*

One of the GPs had concerns that pharmacists might not know how to deal with potential allergic reactions, but said that they had to pre-screen for allergies anyway. Another pregnant/postpartum participant had raised the issue of lacking toilets, but when prompted about this, one of the pharmacists said that he would generally allow waiting pregnant women and children to use the staff toilet if needed.

#### Resources, roles & responsibilities

Lack of resources, especially in terms of staffing was a common theme and meant that less time and effort could be directed to maternal vaccinations. Problems of high workload exacerbated by the pandemic with ‘*a lot of sickness and a lot of burnout’* and redeployment of staff. One midwife mentioned ‘*in one year, from one site we lost about forty midwives*’ who moved to different areas or professions. Another midwife recounted that they had trained high numbers of midwives for the vaccination clinic paid as temporary ‘bank staff’, who ‘*come and go’,* and they now tried to arrange for a permanent staff member, who could also be a nurse or vaccinator. Staff shortages during the pandemic meant that they often had to ‘*pull out a midwife that was booked to do the vaccination clinic to cover the other clinics’* so that they had to ‘*close it once and again’*.

Problems of staffing, space and supply in one hospital vaccination clinic with inconsistent opening hours, meant that one woman ‘*ended up not having the whooping cough vaccine, and not for want of trying’* after unsuccessfully trying to access the vaccine several times.

“I got told by my midwife it’s a walk-in service, so I went […], they said, oh, no, we’ve run out of the vaccine, you’ll have to come back another time.” (W).

Lack of resources also raised questions of roles, responsibilities and priorities with a few midwives arguing that the provision of vaccinations was ‘*more a nurse’s role, than midwives’*.

“I think it is like the hot potato and they’re all passing it to each other, no one wants to take it, it’s like “you do it”, “no you do it” because again it’s not maternity. We are doing it out of our good hearts for our women because we love our women, we want to give a good service but it’s not maternity, it’s not our training, it’s not our profession, it’s not a part of what we do or what we are.” (M).

The GP participants thought that given that ANC was now mainly midwife-led, the provision of vaccinations within maternity made sense. They and a few other HCPs agreed that pharmacies should also increasingly offer vaccines, provided the funding, administration and infrastructure was in place and it was a ‘*united effort’* and clear to clients which pharmacies offered which vaccines. Generally, pregnant/post-partum women thought that midwives were the most important providers to be involved in maternal vaccination-related initiatives.

“I still do think that midwives play a big role because that is who you mostly spend your time with in pregnancy… I know they’ve got a lot to get through, but it definitely doesn’t seem like vaccines is a priority on the list.” (W).

A few participants suggested various providers should offer vaccines to make them more accessible to pregnant women.

#### Health information system and apps

Insufficient bidirectional information transfer between GPs and Midwives was repeatedly highlighted, not only regarding GPs not being informed about patients’ pregnancy. Some midwives also explained that they would not be able to see in their system if maternal vaccinations were received outside maternity care, and one midwife recalled that it happened that a ‘*woman had the vaccine twice’*. Midwives reportedly had to rely on what patients told them, who could not always remember though which vaccines they had.

Lack of data integration and cumbersome documentation requirements had also been among the challenges encountered during the pilot trial of offering Covid-19 vaccines within maternity according to one midwife:

“They use different services and different types of documentation for each vaccine, so it was a nightmare for the midwife having to document every single vaccine on three different systems.” (M)

Due to electronic record system shortcomings and inconsistent documentation, midwives also often did not know whether their colleagues had already discussed vaccines during previous ANC visits and what the discussion outcomes were. This could lead to either vaccine-related discussions being completely omitted or patients getting annoyed if asked repeatedly the same question.

The recent transition from hardcopy medical records and info material to the use of maternity-specific apps reported in some boroughs, was not well received by many pregnant/post-partum women, although some generally liked the idea of a mobile phone app. Some (albeit higher educated) pregnant/postpartum women complained that the app used in their borough was ‘*very confusing’*, not ‘*user-friendly’*, not’*easy to navigate’* or ‘*horrendous*’ with problems of missing or unspecific appointment records or results. Pregnant women were reportedly not told how to use the app and a few participants suspected that midwives were ‘*not trained enough to use the app’* either.

Almost none of the women were aware that their app included links to vaccine-related information leaflets and a few midwives confirmed that usually only other pregnancy-related information/leaflets were accessed by pregnant women.

During the FGD, one mother concluded ‘*the paperless model that we are all striving to get to, which is important, maybe has been too much in the maternity sett*ing’ and that a hardcopy booklet might be more useful for conveying information, including about vaccinations.

App use directly linked to the theme of ‘digital exclusion’ with a few women living in more deprived areas not using the app as they lacked internet access or were ‘*not good at internet’*.

Similarly, ‘language barriers’ were reportedly a ‘*very big problem in terms of accessing vaccinations’* in more deprived areas. A few midwives mentioned that officially translated leaflets were not available in a sufficient number of languages, and calling the telephone interpretation service was often too time consuming for both midwives and clients.

### Behavioural factors

#### Passive versus active decision-making process

Decision-making was not always ‘active’ or even conscious, but ‘passive’ if participants were either not aware of or forgot about the vaccines or if they just went with what had been recommended or what they had previously decided (e.g. during previous pregnancies). Passive versus active decision-making linked to the topic of ‘opt-out versus op-in’ that several participants raised and discussed during the FGD suggesting that it would be better if all maternal vaccines were treated as ‘routine’. Some participants actually saw the pertussis and sometimes also influenza vaccine as a routine and just went with what was recommended trusting the system and/or thinking ‘it had to be like this’.

Many women, however, engaged in an active information seeking process, especially regarding Covid-19 vaccines. Women would do their ‘*own research’* either because they had not received sufficient information from their HCP or did not trust their advice. This active information-seeking seemed to be an expected norm, including by HCP, who saw it their role to let clients know about the NHS recommendation, perhaps signpost them to NHS information material, but then leave it up to them to ‘*do their own research’* and ‘*make their own decision’*.

##### Interaction with HCP/ provider recommendations

According to mothers, provider recommendations regarding Covid-19 vaccinations were not always clear, sometimes ambiguous, and in a few instances midwives reportedly advised against Covid-19 vaccines or agreed that they were probably not safe.

*“I ask her [the midwife] what she thinks about the Covid vaccine and yeah, she said she wouldn’t do it personally because it’s not long research.*” (W).

During ANC visits vaccines were generally only briefly mentioned and pregnant women were often aware of midwives’ high workload and thought there was no time for questions. One woman felt ‘*like on a conveyor belt’*. A few doubted the vaccine importance and/or safety, when midwives did not ask why they did not have their vaccines. A few of the midwives interviewed, however, said they only refrained from probing when assured clients had received the necessary information for an informed decision. Many seemed to try their best to provide the necessary information within the tight schedule, but a few found that most clients had already made up their mind. A few others, however, recounted instances where they were able to ‘*reassure*’ vaccine-hesitant women without trying to ‘*persuade*’ or ‘*convince*’ them.

For midwives it was generally important not to ‘*put pressure’* on patients and to remain impartial, as vaccination was a ‘*personal decision’*. One midwife explained:

“vaccination is kind of like what information can I give you to be empowered to make your own choice rather than I think you should have this so I’m going to convince you […]. Because a lot of midwifery is about empowering people, you know, because they’re not unwell, they’re not poorly […] this is your body and your baby and your choice, so I think maybe we were slightly different from nursing in that sense.” (M).

Similarly, one GP reflected about the need for more ‘*shared decision-making’* and giving patients sufficient information of ‘*both sides of the story’*, including ‘*positives and negatives’*. This was also important regarding potential future complaints, should patients mis-attribute any negative birth outcomes to the vaccine.

Providers’ impartiality was appreciated by many women, but others felt somewhat ‘left alone’ and would have liked more information from midwives, whom they generally trusted. Some suspected that HCP themselves were not really ‘behind the vaccines’ and/or did not know whether especially Covid-19 vaccines were safe.

##### Engagement with information material and social media

As mentioned above, most women did not know about electronic vaccination leaflets via the app and only few had obtained hardcopy leaflets at booking. To fill the information gap many resorted to online searches with the risk of misinformation.

“I hadn’t really had a conversation with anybody about what the benefits of them, taking them were, what the possible side-effects were, what the impacts were, and so all that meant is then I had to turn around and go on Google and do my research. The worst thing to do was go on Google because you’re going to get a whole lot of horror stories…” (W).

Even if trying to find reliable websites, a few women reported that these were not always on top of the search, saying ‘*by the time you click on the NHS website you’ve already received lots of contradictory information’*.

Some women reported actively searching for information and personal stories on social media (Facebook, Twitter, WhatsApp groups, Online mums forums and apps, Blogs, YouTube/TikTok videos), which sometimes exposed them to misinformation and conspiracy theories.

##### Interaction with family, friends & others

Many women recounted various interactions with family and friends, whose opinions and personal stories influenced vaccination decisions in both directions. A few HCPs highlighted the need to involve partners during discussions, especially in ethnically diverse areas. According to many women, however, their partners just went along with what they decided, as it was ‘*their body and their decision*’. A few, especially younger participants sought advice from their mothers.

Many women seemed eager to hear from other currently or recently pregnant women. Some had reportedly been deterred from vaccines after interacting with other pregnant women, including on social media and in waiting rooms. One woman, for example, declined the vaccines after seeing the reaction of other women in the ANC waiting area next to the vaccination room, when the vaccinator told them that they could just walk-in if they wanted the vaccines now.

#### Individual characteristics and influences on vaccination decisions

##### Risk–benefit perceptions

Womens’ vaccination-decision was often motivated by their perception of the risk of the vaccine versus the risk of the vaccine-preventable disease and the benefit of the vaccine versus letting nature take its course and/or taking alternative risk-mitigating measures, such as social distancing, hygiene and wearing masks. This risk–benefit evaluation was often influenced by their own or others’ previous experiences of the vaccine and/or vaccine-preventable disease, and depended on individual factors, such as age. For example, a few younger women associated the influenza vaccine (termed by one participant as ‘*cold vaccine’*) with elderly people. Some concepts were controversially discussed, e.g. underlying health conditions were given as argument for and against vaccination, and some felt pregnancy rendered women more ‘*vulnerable*’, while others denied this.

Overall, many pregnant women felt that the risk of the maternal Covid-19 vaccine was higher than their risk of contracting or falling severely ill from the disease. Many had mainly concerns about potential acute and long-term side effects on the baby and a few wondered why vaccines were promoted during pregnancy, if most drugs and even nasal spray were not allowed. A few single mothers feared that in case of vaccine side effects nobody could care for their babies and/or older siblings.

The risks of the flu and pertussis vaccines were generally perceived as lower, because ‘*they had been better researched*’ and had ‘*been around*’ for longer. However, many women saw only the importance of the pertussis vaccine, because it ‘*benefits the baby’*. Most HCP seemed to generally find it easier to recommend and advise people on the pertussis vaccine as it was ‘*more for the protection of the new-born baby rather than the mum’*.

HCP who had personally witnessed the severity of vaccine-preventable diseases among pregnant women, seemed more likely to recommend vaccines. A few women observed that hospital midwives were more likely to recommend the Covid-19 vaccine than community midwives.

##### Knowledge and skills

Many participants felt they lacked the knowledge to make an informed decision (or provide specific advice), especially regarding Covid-19 vaccines after the change of guidance.

“There was also a lot of conflicting information from the authorities so a lot of women were quite nervous about it and we could see why…” (O).

Some women lacked awareness and/or basic information and were too shy or had no opportunity to ask questions. A few HCPs suggested that better general health education was needed. Accordingly, a few participants confused vaccinations with other types of injection or blood tests, or thought the flu vaccine could cause flu. As mentioned above, limited digital and language skills made it more difficult to access information.

Some, especially higher educated women, had sought second opinions and/or asked midwives questions. After being referred to generic NHS and other websites, however, a few were still ‘*craving*’ for further ‘*mid-level*’ information that was neither too basic nor too scientific. One midwife found a poster containing statistical charts helpful to explain current Covid-19 vaccine research results to pregnant women that had actually been targeted to HCP. A few other midwives gave detailed examples of their commitment to relay relevant information and have an honest dialogue with clients within the given time.

Many HCP, however, shared pregnant women’s concerns regarding insufficient research on potential longer-term side effects of the Covid-19 vaccine, and a few mentioned the lack of inclusion of pregnant women and representation of minority ethnic groups in vaccine trials.

One midwife felt that information was withheld from midwives and patients, when she heard from a client that the pertussis vaccine is given in combination with other vaccines (against diphtheria, tetanus and poliomyelitis).

None of the HCPs indicated that official ‘vaccine leads’ were included in their team, but GPs mentioned that if in doubt they would first ask the nurse who provides vaccinations in their practice for further information.

##### Emotions and Trust

Emotions and trust reportedly played an important role in decision-making. One midwife believed that society was divided into ‘*either pro or anti’* vaccine and there was no ‘*middle ground’* because of ‘*fear*’:

“*So either you have fear of the disease or you have fear of the vaccine, and whatever fear you have, that's what you go with*.“ (M)

Fears were reportedly triggered through own or others’ personal stories, especially if linked to miscarriages and fertility issues. Only few women mentioned fear of needles. A few mothers also mentioned that during pregnancy they were different from their normal self, and many were more anxious about what to ‘*put in their body’*. One GP participant said that only after being pregnant herself, she could ‘*totally understand’* why pregnant women were often perceived as the ‘*most difficult patients’* and she and others believed vaccination campaigns should be better targeted at pregnant women to relieve their anxieties.

One woman with a history of fertility issues was upset when she was approached in the ANC waiting room during a Covid-19 vaccination campaign. She felt that the pressure around Covid-19 vaccines led to mistrust against other vaccines, too. She found it unfair that the Government gave women the right to abortion on a ‘their body their choice’ basis, but conversely ‘pressed’ her to take a maternal vaccine she did not want. A few participants felt also annoyed by constant Covid-19 vaccine-related NHS text message reminders without reply options.

Many participants generally trusted their HCP and the NHS, but the afore-mentioned government’s change of guidance regarding maternal vaccinations led to confusion and distrust.

A few participants linked safety fears and mistrust to historical events, including the Thalidomide crisis and human rights abuses among minority groups with fears to be ‘*used as an experiment*’. One midwife with a minority ethnic background explained that the ‘*push for the BAME community*’ to have the Covid-19 vaccine first due to increased susceptibility, was perceived as ‘*let’s all wait and see but you guys go first*’.

One participant lost trust after feeling discriminated against by her initial midwife, and requested a different midwife. She subsequently had a very short booking visit without information on vaccinations.

Some women also lost trust due to misinformation (e.g. relating to ‘autism’) and conspiracy theories. One mother believed, for example, claims circulated by South-American HCP via social media that the Covid-19 vaccine was injected to kill people or make them infertile. She also worried about a mark on her baby’s arm that she attributed to the maternal flu vaccine.

One midwife noted that increasingly hearing from clients about vaccine-related safety concerns since the pandemic, made her now question vaccines more, where she had previously just trusted NHS advice.

##### Cultural norms, philosophy and beliefs

As mentioned, many women considered other pregnant women’s vaccination-decisions. Many women from abroad were influenced by their country of origin’s vaccine-related culture and behaviour, e.g. some saw no need for routine maternal vaccines not recommended in their home countries. Conversely, another woman was eager to get the maternal Covid-19 vaccine, as her sister in America said that ‘*a lot of her friends were pretending to be pregnant so they could get the vaccine as a priority in America*’ and many were having it while actually pregnant.

A few women took vaccines for altruistic reasons, while a few others, however, opposed the framing of messages around altruism.

Religious and philosophical believes, including the concept of ‘*nature will take its course’* whether we vaccinate or not, could influence vaccination decisions, too. A few participants preferred to keep things ‘natural’ where possible; one woman felt more comfortable to take the pertussis vaccine, after obtaining a preventative ‘*antidot*’ against vaccine side effects from her homeopath.

One midwife told they ‘*struggle during Ramadan, women will decline’* vaccines, although she thought that pregnant women were exempt.

##### Attitude towards mandatory Covid-19 vaccines

During interviews the ‘*tough, touchy subject’* of mandatory vaccines for HCP (which the government had planned to introduce before a sudden U-turn) was mentioned and further explored. While one midwife could not see a difference to their mandatory Hepatitis-B vaccine, most HCP interviewed, although generally pro-vaccine, opposed mandatory Covid-19 vaccinations. Some midwives felt betrayed, and said they no longer needed a vaccine, as most had contracted Covid-19 after being forced to work ‘without PPE’.

Resentment was also felt by a few pregnant woman who took the Covid-19 vaccines only for travel and/or work reasons, while a few others did not seem to mind.

### Participant recommendations

Many mothers emphasized the need for clearer provider recommendations, more time for bi-directional dialogue and more information. Some participants said further HCP training, more resources and ‘mid-level’ information material were needed. A few women made specific suggestions on how to access more specific information, e.g. via telephone help lines, chatbots and interactive Q&A websites.

A few HCPs also highlighted the need to educate the wider community about maternal vaccinations given social influences on pregnant women’s vaccination decisions. Some participants suggested campaigns needed to be more targeted at pregnant women and include for example videos with ‘real people’.

To make it easier to access maternal vaccinations, FGD participants recommended:

“*it should be an opt-out rather than an opt-in. […] Meaning that it’s standardised, it’s in the calendar and you just need to decline it, if you don’t want it, but that if not you just go ahead with it. Rather than actually having to proactively go and get it, and discuss it.*”

A few midwives were generally against a ‘*blanket approach’* and felt that individual risk assessments were needed. One midwife also requested the re-introduction of the 15-min observation rule post-vaccination in case of potential allergic reactions.

As mentioned above, some HCPs suggested shifting roles and responsibilities regarding maternal vaccine provision.

Many participants emphasized that better IT systems were needed and training in their use. A few HCPs mentioned that in two South-London NHS Trusts a new integrated system would be launched in 2023 with new patient portal and app, and a phased-in approach for different functionalities which might solve some of the problems.

Some participants found that especially for service users lacking digital skills or internet access, hardcopy information material should be provided. Similarly, language barriers needed to be addressed:

“I think we definitely need to be more proactive in trying to offer vaccine information in as many languages accessible as possible […] it may need to go as far as even educating interpreters about how to deliver information about vaccines because we have many that don’t actually can interpret what the vaccines are.” (M).

## Discussion

Our study shed light on various structural, organisational and behavioural factors that could explain the low maternal vaccination uptake in socially and ethnically diverse areas in London during Covid-19. Especially during the acute phases of the pandemic, maternity services in South London experienced high staff turn-over and other pandemic-related pressures that exacerbated existing structural challenges and raised questions of roles, responsibilities and priorities. Political factors contributing to these challenges included the proposed mandatory vaccination of HCPs and the change of guidance regarding maternal Covid-19 vaccines that confused many ANC service providers and users alike.

Structural and organisational factors, including workflow and IT systems shortcomings, meant that depending on additional individual and social factors, it was relatively straightforward for some pregnant women, but confusing and challenging for others, especially those with specific concerns, insufficient time and/or limited digital and language skills to access reliable information and vaccines at different locations. Not only time pressure, but possibly also discrepancies in the attitudes of midwives and pregnant women might have been contributing factors. Many women wanted stronger or clearer recommendations, detailed information and discuss vaccinations already at an early stage, while many midwives wanted to avoid overloading women with information that they would forget. At later stages, some pregnant women wanted midwives to probe and discuss why they had not had their vaccines, but some midwives seemed to shy away from probing, as they were anxious to respect their clients’ choice. Inconsistent recommendations and signposting, in addition to the general notion that pregnant women had to do their ’own research’ meant that pregnant women often turned to the internet and social media to fill information gaps with the risk of misinformation that compounded (pre-existing) uncertainties and mistrust.

Fear of negative short- and long-term effects of a newly developed vaccine meant that most woman in our sample declined or delayed maternal Covid vaccines. Conversely, most women opted for the pertussis and many for the influenza vaccine. The latter was perceived as less important by many, including because its assumed purpose was to protect only the mother and not the baby. Eight interviewees took none of the maternal vaccines.

### Strengths and limitations

A study strength is that we conducted a large number of interviews, including also pregnant/post-partum women who took none of the vaccines,. We also obtained in-depth accounts from women from socio-economic and ethnic backgrounds that have been less likely to volunteer to participate in comparable qualitative maternal vaccination studies. For example, in four previous studies (conducted in the UK and Ireland before maternal Covid-19 vaccines became available) none or only one interview was conducted with participants of Black ethnicity [23, 24, 37, 46]. As with all qualitative studies, however, results from this study cannot be generalised and people who volunteered to participate might be different from those who did not. We noticed, for example, that most midwives included in our study were generally pro vaccine and reportedly recommended all maternal vaccines, which did not seem to match the experiences of many included pregnant/post-partum women. Nevertheless, it is another study strength that we included perspectives from both service users and providers, where most other comparable recent maternal vaccination studies included only one perspective [46, 47], with few exceptions, including the studies cited below [22, 48, 49].

Previous international studies called for further research on the reasons for lack of recommendations, especially among midwives [20, 26–28]. Our comparably long interviews (about 50 min average, compared to 5–20 min elsewhere [46, 50]) have provided rich insight on possible reasons for inconsistent recommendations and low vaccine uptake, including on practical challenges (further discussed below). Accounts from other providers, such as pharmacists, have been included for additional insight, and could be further explored in future studies.

### Interpretation of results and implications

Already prior to the Covid-19 pandemic in 2019, in the context of resurging vaccine-preventable childhood diseases and threats of influenza pandemics, the World Health Organisation (WHO) listed vaccine hesitancy among the top ten global health threats [51]; among reasons it highlighted ‘lack of confidence, complacency and convenience’ [52].

By the time of the Covid-19 pandemic, various factors, including spread of misinformation via social media, decreasing trust in institutions, and failings within health systems had diminished confidence among some populations, including among marginalised groups [53].

In an overcrowded metropolis like London, Covid-19 hit early when people and systems were unprepared. During times of distress, people are in particular need of clear and up-to-date information and reassurance from trusted leaders, in the absence of which misinformation and conspiracy theories can flourish [54, 55]. This may be especially true in what one of the interviewed providers described as a ‘very deprived and ethnically diverse and historically distrusting population’. Besides, pregnancy in itself renders women more suspicious of what to ‘put in their bodies’ and particularly concerned about adverse effects of new vaccines (developed during trials that excluded pregnant women) [25]. This also explains why some pregnant women felt left out by the more general Covid-19 vaccination campaign and ‘craved’ for more information and reassurance that overworked HCP were often unable to provide due to lack of time, training and resources.

To reduce the threat of misinformation, experts have highlighted the importance of partnerships between the vaccination community and social media platforms; a communication strategy that gives a voice to a ‘silent majority’ with positive beliefs about vaccination; and focusing on understanding context-specific factors among various populations and innovative approaches to address these [53].

Our study provides important new insight on context-specific structural and organisational factors that may be addressed by future research, given that so far the evidence-base for interventions to increase immunisation coverage in the current British health system is limited [56–60]. We have shed some light on why previously recommended electronic vaccination prompts and call/recall systems [22, 47] are still not in place or feasible, including because GP databases no longer routinely show if a patient is pregnant, or because both midwives and clients often do not know how to use the limited functions of their maternity apps. A new more integrated electronic health record system and app will be introduced in two London Boroughs soon [61]. Findings from our study could inform necessary improvements to be implemented within this or other future new IT systems. However, digital exclusion and language barriers, also mentioned elsewhere [62, 63], should be addressed, including through better training in app use and alternative modes of information in various languages.

Our study also identified the need to streamline processes to make it easier for women to access timely vaccinations. Recent evidence on default opt-out policies for maternal vaccines, as suggested by our FGD participants, is missing or equivocal [64, 65]. However, a promising recent maternal vaccination delivery model involved phone calls to pregnant women if appointment booking was missed during initial ANC visits, and reminder texts prior to scheduled pertussis and influenza vaccination appointments that coincided with ANC visits [46]. Optimized models are also needed for new vaccines, as our participants mentioned unsuccessful attempts of offering Covid-19 vaccines within maternity services. Research on alternative ways should involve all stakeholders, consider pre-existing infrastructure and preferred roles and responsibilities. Pharmacists are reportedly eager to take on greater roles, and further enquiries into necessary structural changes and resources to facilitate this are needed.

In line with our findings, researchers in Spain observed that changes of maternal Covid-19 vaccination guidance happened fast, and were not always implemented by HCPs due to mistrust, which led to vaccine hesitancy [49]. According to our study, confusion around changes of guidance, and the fact that at vaccination centres women were routinely asked about pregnancies for monitoring reasons, reportedly discouraged some from accepting the Covid-19 vaccine. Plans to introduce mandatory vaccines for healthcare workers in England (prior to a sudden U-turn in January 2022) also affected midwives’ trust in the health system and the vaccination programme [66].

Many of the individual-level behavioural factors on both provider and user side that we observed, including related to personal attitudes, emotions, trust, conspiracy theories, misperception, knowledge, skills, risk/benefit perception, cultural norms, philosophy and beliefs have also been observed elsewhere not only for maternal, but also for other types of vaccines and are not discussed in detail here [20, 25, 67]. However, we highlight several key points important to our specific setting and population that may be relevant for the development of future tailored interventions.

The fact that participants used underlying health conditions as arguments for and against vaccines, and either emphasized the ‘vulnerability’ of pregnant women or explicitly denied it, is relevant for framing and tailoring of vaccination messages. These should also consider that a few interviewees felt too young for maternal flu vaccines, after seeing campaign pictures showing only elderly people. Others felt that for Covid-19 vaccines, pregnant women had been made a priority far too late. Carefully framed messages should also emphasize the benefits of maternal influenza and Covid-19 vaccines for the baby [68]. We found that generally only maternal pertussis vaccines were portrayed as benefiting the infant, in keeping with a study conducted in 2018 [38]. Message framing around altruism and ‘community immunity’ recently suggested for the general population might not work in all contexts among all ethnic groups [69], in line with resentments expressed in the context of maternal vaccines by a few of our participants.

Some of our findings around ethnicity and trust in vaccines and health systems have also been reported elsewhere, including a study conducted in East London in 2015 [22]. in this study, similar to ours, black participants reported preferably seeking advice from female family members and friends, including their mothers, suggesting that providing information more widely may be important. We found that women were influenced by their country of origin’s vaccine-related culture and behaviour, and such differences may need to be explicitly discussed, as suggested by a recent study on flu vaccinations among Polish and Romanian communities in England [70]. Social influences from other pregnant women in clinic waiting areas that our study found, could be further explored in future studies along with potential interventions to provide factual information in this setting, e.g. via television screens. Personal stories can be powerful deterrents or motivators, especially if linked to emotions [71]. Women in our study often referred to emotive personal stories they heard from others, including on social media, as arguments against vaccination. In contrary, while HCPs who had witnessed more morbidity in hospital seemed more likely to recommend vaccines, they seemed to only rarely communicate personal stories from their own practice to pregnant women. Indeed, there seemed to be a social norm that HCP should remain neutral (in both directions), just transfer the information from the NHS and leave it up to women to make ‘their own decision’.

This links to another notable context-specific key point. In our study, pregnant women were generally expected to do their ‘own research’, which reportedly was not the case in Spain [49]. In New Zealand, however, midwives (other than nurses and pharmacists) also reportedly followed a ‘your body/your choice’ philosophy and encouraged women to seek information for themselves [48]. The authors were concerned, however, that this could inadvertently render women doubtful about the benefit and safety of maternal vaccines. In our study, a few participants also noted that not all women had the skills and time to do their own research. Even a few highly educated women felt left alone with the responsibility and burden to make their own decision during times of uncertainty, and craved for more personalised and specific information and reassurance from HCPs. Midwives might need to be made aware of this and that women from minority ethnic groups might want midwives to probe for reasons why they had not been vaccinated and discuss underlying concerns (without putting them under pressure). There is an ongoing discourse around different styles of patient-provider vaccine communication, including the advantages and risks of presumptive versus participatory approaches [22, 32, 72]. Participatory and motivational interviewing approaches [73, 74], and proposed decision-making tools for personalised support [75] are more in line with the ‘informed choice’ ideals at the core of midwifery practice, but would require more staff time for relationship building and training. Others have also highlighted the need to train HCPs to recognizing various cognitive biases [76] and deflect misconceptions without eliciting resistance [77].

Many participants in our study indicated that HCP were often unable to respond to specific vaccine-related questions. Service users and providers in our study and elsewhere felt there was insufficient (long-term) research on Covid-19 vaccinations in pregnancy [78], in keeping with recent calls for inclusion of pregnant women in clinical trials [79]. This also supports calls for better pre-service training and continuous education for HCP with updated information on new vaccines and opportunities for open and honest dialogue [77, 80]. HCP should also be able to advise on what exactly vaccines contain. For example, omitting that the pertussis vaccine is combined with diphtheria, tetanus and polio might provoke mistrust, as also recently observed by researchers in Spain [49]. Pregnant women should not only receive the factual information and empathic support they need to make vaccination decisions; Pregnant women should also be given tools to recognise potential misinformation [81]and this may require structural changes to reduce people’s exposure to misinformation disseminated via social media [25, 82].

Our study has confirmed the importance of unambiguous provider recommendations and described various behavioural determinants of maternal vaccine uptake during the Covid-19 pandemic. It is important not to tackle these in isolation, but to co-produce solutions that also address the deeper structural and organisational factors we have revealed.

Ultimately, such strategy will require further resources and careful balancing of priorities. At the time of writing, the UK experiences the ‘triple burden of flu, RSV and Covid-19’ and increased group A streptococcal infections, in the context of concerns over antimicrobial resistance and potential human spill-over of avian flu outbreaks [8, 83–85]. Conversely, successful RSV vaccine trials hold promise that new vaccines will likely become available soon to protect mothers and infants [10, 11]. It is therefore crucial that investments are made to improve maternal vaccination programmes in areas of low uptake.

## Conclusion

There is need for clearer recommendations and information provision to alleviate pregnant women’s burden of decision-making. Processes need to be streamlined to make it easier for women to remember vaccinations and less likely to forget. We identified important context-specific factors on structural and organisational levels, relating to roles and responsibilities of different providers, workflows and IT systems that should be addressed in the future. This includes improving maternity apps and training both, pregnant women and midwives in their use. Providers also need more resources and training to address their lack of knowledge, especially on new maternal vaccines. Improvements on the organisational and provider level, and a communication and education strategy that considers women’s personal circumstances and (digital/language) skills, should make it easier for pregnant women to obtain the information and reassurance they need, navigate the ANC system and access vaccines at convenient locations and times. Beyond improving the quality of service provision, addressing the deep seated concerns that many women in our study population expressed, will require co-produced tailored interventions and programmes.

## Supplementary Information

**Additional file 1.**

**Additional file 2.**

**Additional file 3.**

**Additional file 4.**

## Data Availability

We have included study materials and anonymized qualitative data extracts in the supplementary files. Sharing of further anonymized qualitative data extracts on reasonable request would have to be in agreement with data protection laws and subject to appropriate ethics committee approval. We welcome readers to get in touch with the corresponding author for any collaboration and/or to discuss further results regarding specific concepts and participant recommendations.

## Abbreviations

ANC: Antenatal care
GP: General practitioner (family doctor)
NHS: National Health Service
NIHR: National Institute for Health and Care Research
HCP: Healthcare provider
VIP-IDEAL: Vaccination in pregnancy-ideas, experiences and attitudes
W: Pregnant/post-partum woman
M: Midwife
O: Other provider
