# Supplementary material for: “Figuring stuff out myself” – a qualitative study on maternal vaccination in socially and ethnically diverse areas in England
Source: BMC Public Health. 2023 Jul 21;23:1408. doi: 10.1186/s12889-023-16317-z (PMC10362695; doi:10.1186/s12889-023-16317-z)
Supplement: Supplementary file 1 — Additional file 1. [file 12889_2023_16317_MOESM1_ESM.pdf]

SUPPLEMENATRY FILE 1 – Details on methods of VIP-IDEAL study, 2022

A Qualitative approach and theory

As mentioned in the manuscript, the study design and implementation were guided by a critical realist paradigm [1, 2] and informed by discussions with patient and public involvement (PPI) contributors and a conceptual framework developed based on existing theory and research[3-12] (which was then adapted as explained in section C)

Below we provide details and examples on how 1) the critical research paradigm in the context of the researchers' positionality, 2) the PPI work, and 3) the initial conceptual framework guided and informed the study design and implementation.

A.1 Positionality and critical realist paradigm

The researchers involved in fieldwork and analysis maintained self-reflexivity throughout the research process, in order to become aware of their assumptions and clarify presuppositions and how these could influence the research process. The researchers' reflexivity informed their positionality throughout different stages of the research process[13].

Below, we have pasted an extract of the positionality stated at the design stage of the research process by the lead researcher (with sections that included personal information excluded). It describes what we mean by and why we chose a "critical realism paradigm".

> *"My positionality and theoretical approach to this research have been influenced by my training, professional development and experiences that (…) do not easily fit into traditional philosophical categories. In terms of ontology, epistemology, methodology and axiology, I therefore often position myself in the middle of a continuum between two extreme positions. For example, my past (…) are traditionally associated with positivism, naïve realism and objectivism[2, 14, 15]. However, other experiences from the past fifteen years (…), have tended to push me in the opposite direction of the continuum towards interpretivism, relativism and constructionism[2, 14]. My (…) and the fact that I lived and worked in various high- and low-and middle- income countries (…), allowed me to gain multiple perspectives, learn about different versions of social reality, and become aware of the importance of tacit knowledge, and the situation-, context- and time-dependency of meaning. I am a proponent for the decolonisation of global health, which at its roots requires acknowledgement that "there are many ways of being and doing, unlearning the universality of being and actively engaging with pluriversalities[16]". I also believe in a holistic, systems approach towards human needs, am interested in emancipatory approaches and in 'crowdsourcing', which overlap with the theoretical approaches of 'human-centred design' and 'participatory action research'[14, 17-21].*
>
> *Taking all this together, my position in terms of research paradigm is closest to 'critical realism', which represents a combination of views that contrast with those associated with both, traditional positivist and interpretivist positions[1, 2]. According to critical realism (CR), there is a reality that exists independent of our thoughts about it, and while we can become more confident about what exists by observing, existence itself is not dependent on observation. CR also sees the social world as layered, complex and an open system and characterized by change. Critical realists are typically pragmatic in their approach to methodology and methods, and in terms of axiology*

often include emancipatory objectives and believe that when phenomena are under investigation it may be possible to identify how these features may be influenced in order to ameliorate harmful effects or to enhance beneficial effects[22].

Regarding my positionality in terms of the insider/outsider dichotomy, I agree with researchers who argue that this dichotomoy may be an artificial construct and in reality a continuum with multiple dimensions, and that a researcher may inhabit multiple positions along this continuum at the same time[13, 23, 24]. For example, regarding research participants from group 1 (pregnant/post-partum women), I could be seen by some participants as an 'insider' in that I am (…); others, however, will see me as an outsider, including in that (…). The advantages and disadvantages of being an outsider versus insider (and the inverse) and their impact on the quality and trustworthiness of research, have been subject to continuous debate[13]; these debates, as well as discussions within the research team and with public and patient involvement (PPI) contributors will continue to inform my self-reflexivity during the course of the research process.

My positionality in terms of the subject of investigation, i.e. vaccinations during pregnancy, is placed on the outer 'pro-vaccine' pole of a 'continuum of vaccine acceptance', with acceptance of all recommended vaccinations, whilst the other pole would constitute refusal of all vaccinations. However, I am aware of the complexity of decision-making and multitude of factors that can influence vaccination uptake (as outlined in figure S1) and the importance of truly listening to people's concerns.

Having said all this, I aim to achieve 'empathic neutrality' during field work and analysis, while acknowledging that this can never be fully attained. There will always still be some form of bias or subjectivity, and there is no completely 'neutral' or 'objective' knowledge[25]. It will therefore be all the more important to maintain self-reflexivity, discussions with the team and transparency about the research process."

The researchers believe that while some aspects of individuals' positionality may be culturally ascribed or generally regarded as being fixed (such as ethnicity and gender), other aspects may be highly situation and context-dependent, so that overall, their positionality may not be static over the course of the research process[13]. Different aspects of the researchers' positionality could slightly shift (temporarily or on long-term), influenced by interactions with participants, self-reflection and discussions with team members.

[For example, although in terms of subject of investigation, the lead researcher placed herself on the outer 'pro-vaccine' pole of a 'continuum of vaccine acceptance', but aimed for 'empathic neutrality', this did not mean that she was not influenced by participants during interviews and analysis in one way or another. She had, for example, initially a relatively neutral attitude on mandatory vaccines for healthcare workers (HCW), and the topic had not been included in the topic guide. Her attitude, however, shifted during the research process in the context of the experiences of HCW during the initial acute phases of the Covid-19 pandemic. In London, most midwives had reportedly already been infected, after having to work without personal protective equipment (PPE), and many did not see why they should be coerced to get vaccinated when it was too late, because they had not received the protection they needed. The lead researcher therefore probed more into this direction in subsequent interviews, gave more attention to the topic during the concurrent analysis and was tempted to give it more space in the results section of the paper. However, she then agreed with co-authors to mention it only briefly in the report for word count reasons and since it was not a central topic of the research question, but include related quotes in the supplementary file.]

The lead researcher discussed various topics and themes that were raised during data collection and generated during the analysis with her colleagues, including OOA, who contributed to part of the field work and the analysis. Below an extract of the positionality stated by her at the design stage of the research process.

> *"As an anthropologist, I am acutely aware of my positionality and how this manifests in my reflexivity. I am entirely bound by my body, and as such my experiences in this research will be mediated through my body. In the words of Okely and Callaway, 'as an anthropologist, I cannot escape myself; nor should I try[26]'. In this research project, as all others, I will endeavour to make my body a productive premise since '[t]here is no option for us as social members or as social anthropologists but to proceed from the premise of self[26]'. As a female Nigerian anthropologist having grown up in the United Kingdom, doing research with pregnant women of ethnic minorities in South London will produce a very unique meeting point[27]. […] "*

A.2 Patient & Public Involvement (PPI)

The design and implementation of the study has been informed by discussions with and feedback from members of the public and the target population, including by commenting on and providing ideas for the recruitment strategy, and how to communicate study information to potential participants.

This included for example discussions with GP clinic staff on the feasibility of running a database search to identify eligible pregnant and post-partum women and send them text messages about the study (with a link to the study website and the researcher's contact information). The researchers learned that at least in some London Boroughs pregnancy was no longer routinely coded in the GP patient database (which was later also reported during some of the interviews), but that they could send text messages or emails to eligible postpartum women. The researchers also sought advice from members of the target population (pregnant/post-partum women) on the length and content of the text messages.

In addition, the researchers discussed with pregnant/post-partum women how best to recruit participants from within the community. (This became even more important, when the researchers found that those participants who responded to GP text messages tended to be more pro vaccine than those recruited from within the community.)

For example, the poster and social media advertisements used for recruitment were designed with support from pregnant/post-partum women of black, white non-British and mixed ethnic groups living in socially and ethnically diverse communities in South London. They suggested/chose poster colours and photos that they thought would appeal to potential participants from different backgrounds, including black, white non-British, mixed and other backgrounds. To account for differing levels of digital skills of potential participants, PPI contributors also suggested that posters should include both a barcode leading to the study website and slips with contact information to remove.

PPI contributors also helped by advising through which channels and at what events the target population could be reached, including at which free parent-child groups. One

important point that was confirmed during recruitment was that one had to actively go to places where pregnant/post-partum women congregate. In addition, participation had to be made as easy as possible (which the lead researcher could very much sympathise with, given that she has children herself). Interviews were therefore conducted at times and places most convenient to participants and where babies or older siblings could attend and play with toys (e.g. at their homes or in local parks near a playground).

In addition, PPI contributors reviewed, commented on and helped to revise the participant information sheets, informed consent forms, topic guides and lay summary of the results that will be made accessible to participants/the public once this article has been published.

A.3 Initial conceptual framework

The study design was informed by the initial conceptual framework below (Figure S1) that was created by the researchers based on their own expertise, and on existing theory and literature [3-12]. For example, in line with the Ecological model by McLeroy et al, 1988 [6], we focused not only on individual and interpersonal factors, but also factors on the organisational (here health systems) and community level in the context of public policy. Our framework was also informed by a previous model by one of our co-authors (HJL) and colleagues. In 2012, the Strategic Advisory Group of Experts (SAGE) on Immunisation had established a Working Group that drafted a "Model of determinants of Vaccine Hesitancy" that was later used in a literature review to categorise concerns surrounding vaccination during pregnancy[5, 9]. The model was organised around three overarching categories, including a. contextual influences (not only political and institutional, but also socio-cultural factors, historical influences, and the media environment), b. individual and group influences and c. vaccine and vaccination-specific issues.
The creation of our model had also been influenced by some of the co-authors' expertise in the fields of health policy and systems and quality of care research, as well as behavioural research and intervention development. We therefore ensured to visualise both the provider and client side within the wider institutional, social and political context and also incorporated the COM-B (Capability, Opportunity, Motivation and Behaviour) model by Michie et al, 2011[7]. This model had also recently been used by Castillo et al[3] (alongside the Theoretical Domains Framework[28]) when illustrating modifiable factors that would facilitate midwives to offer and administer vaccinations in pregnancy.

Our initial conceptual framework influenced our study design and implementation, not only in terms of choice of study participants (i.e. including pregnant/post-partum women, healthcare providers and other stakeholders), but also the lines of questioning during interviews and FGD. Although the topic guides (See supplementary file 3) were designed to elicit information on both structural factors (e.g. by enquiring about experiences with the health system) and behavioural factors, we intentionally asked more general and open questions that would allow participants to tell their own stories (before probing further, where appropriate, with the domains of the framework in mind). During the analysis, we adapted our framework to better match the data, as further described in section C below.

Figure S1 – Initial conceptional framework of factors influencing maternal vaccine uptake based on relevant literature[3-12]

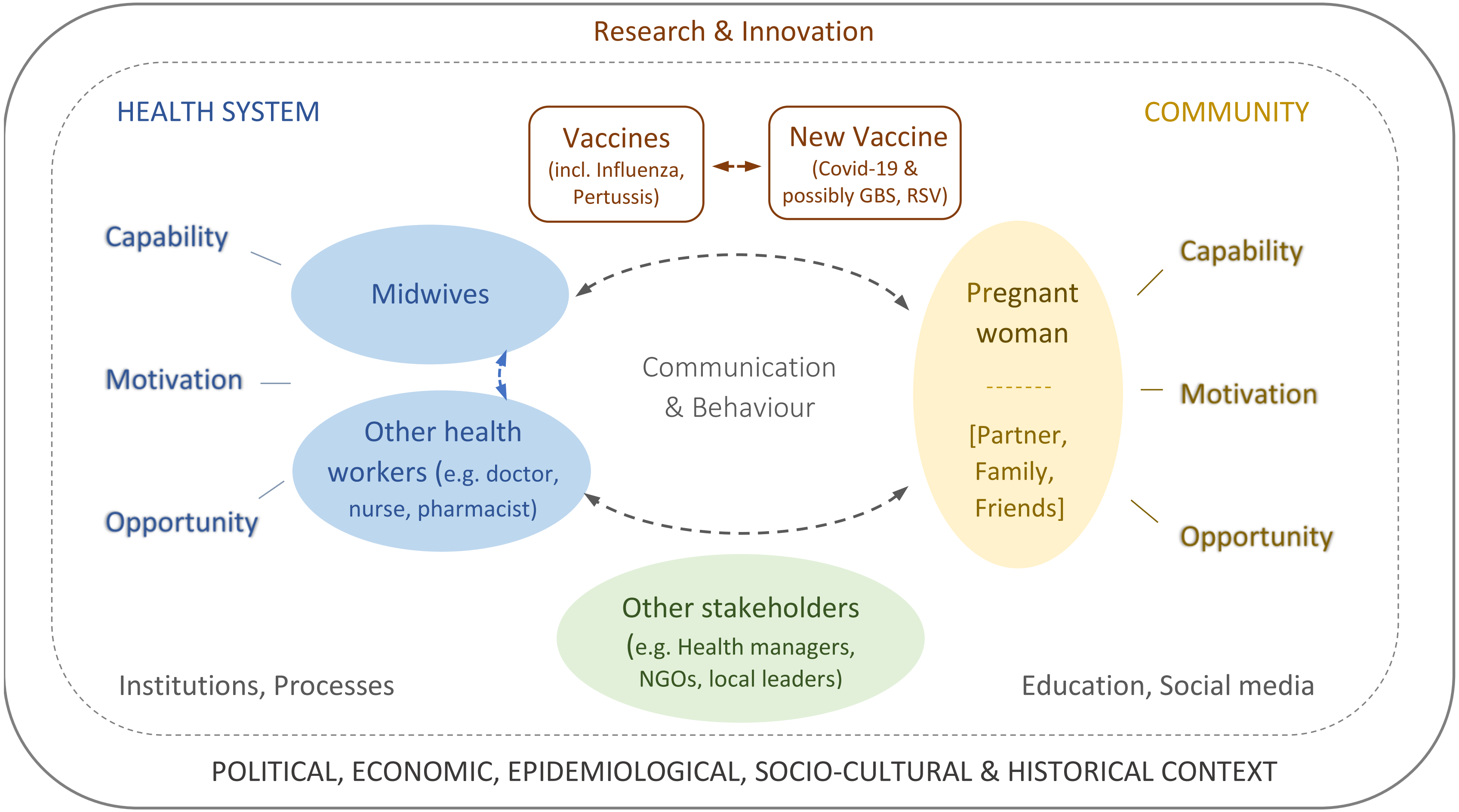

B Study population and setting - details on the recruitment of pregnant/post-partum women

We screened participants whom we had approached during parent-child sessions or who had come in touch with us in response to other recruitment efforts (e.g. posters, text messages, WhatsApp or email lists) for eligibility.

An initial 55 pregnant/post-partum women were eligible, among whom six individuals were unresponsive, two declined (for time reasons and unwillingness to disclose any personal information) and 47 were willing to participate, among whom 38 were recruited. Recruitment details are provided in Table S1 below.

Table S1 – Recruitment of VIP-IDEAL study participants (pregnant/post-partum women, total N=38)

Recruitment via	Interviewees (n)	FGD participants (n)	Total
GP clinics	7	5	12
Parent-child groups	9	0	9
Social media*	5	0	5
Poster*	5	1	6
Word of mouth	5	1	6
Total	31	7	38

C Details on data collection and analysis

According to participants' preferences, interviews were conducted either via video call (n=33), telephone (n=6) or face-to-face at participants' homes or nearby public spaces (n=12), and lasted 22-93 minutes (about 50 minutes on average).

We had budgeted for professional translation/interpretation services, and the researchers themselves also speak further languages. Translation/interpretation had only been necessary, however, for two South-American participants. Another participant preferred her husband to help with interpreting from Persian to English where needed. Otherwise, no other adult non-participants were present during the interviews/FGD, but many pregnant/post-partum women had their babies/older siblings with them, which usually disrupted the discussions only slightly.

SB obtained informed consent and completed all interviews and SB & OOA facilitated the FGD, introducing themselves as university public health researchers, not involved in clinical NHS work. As mentioned above, both SB & OOA completed a positionality statement prior to conducting the qualitative research, and maintained self-reflexivity not only during field work, but also during analysis, aiming to avoid imposing own assumptions and pre-defined theories onto participants' narratives[13].

We followed the six steps of thematic analysis including 1. familiarizing ourselves with the data, 2. generating initial codes, 3. searching for themes, 5. defining and naming themes, and 6. producing

the report[29]. We followed these steps in an iterative way and alongside data collection. In the early stages, SB and OOA duplicate coded the first few transcripts inductively line-by-line. They then discussed with the team the results of this initial coding, and reflective notes of further interviews, in the light of the initial conceptual framework above (Figure S1). Based on these discussion, SB and OOA then developed a provisional coding framework. According to this framework, an initial sample of transcripts was coded by SB and duplicate coded by OOA and AI. Then, SB coded all remaining transcripts, but transcripts from younger participants were 'duplicate read' by the youngest team member (AI) and transcripts from black participants by a black team member (OOA) and subsequently discussed. SB initially coded and analysed data from pregnant/post-partum women (interviews and FGD) and from health service providers (interviews) in two separate NVivo databases, but looking for and comparing patterns and newly generated themes across the whole dataset, thereby also searching for deviant cases. At the later stages, after all data had been coded in NVivo, SB created a word document in which she analysed data from both participant groups combined.

As data collection and concurrent thematic analyses progressed, SB presented preliminary results to the team and repeatedly adapted the conceptual framework based on discussions. The conceptual framework helped us to visualize how different themes were linked. We initially still used the Capability, Motivation and Opportunity categories from the COM-B model[7]) and mapped themes accordingly, but later moved away from it given the increasing complexity and overlap of themes and categories. Our final conceptual framework (Figure 1) includes a time component/directionality, and visualizes how pregnant women navigate the antenatal care system during their pregnancy journey, and the encounters and potential factors that may influence their decision-making. It is implied that not only the pregnant woman, but also the midwives, other service providers, and indeed all other persons that the women interact with are influenced by a long list of own individual characteristics and behavioural influences (that could theoretically be mapped to separate COM-B models), that determine whether and how they recommend vaccines to the pregnant woman.

As shown in Table 3 of the results section, we finally organised results by two overarching categories (structural and behavioural factors) with an additional separate section on participant recommendations.

References

1. Bahaskar R: **A Realist Theory of Science**. London, UK: Routledge, Taylor & Francis e-Library; 2008.
2. Gilson L, Hanson K, Sheikh K, Agyepong IA, Ssengooba F, Bennett S: **Building the Field of Health Policy and Systems Research: Social Science Matters**. *PLOS Medicine* 2011, **8**(8):e1001079.
3. Castillo E, Patey A, MacDonald N: **Vaccination in pregnancy: Challenges and evidence-based solutions**. *Best Practice & Research Clinical Obstetrics & Gynaecology* 2021.
4. Kilich E, Dada S, Francis MR, Tazare J, Chico RM, Paterson P, Larson HJ: **Factors that influence vaccination decision-making among pregnant women: A systematic review and meta-analysis**. *PLoS One* 2020, **15**(7):e0234827.
5. Larson HJ, Jarrett C, Eckersberger E, Smith DMD, Paterson P: **Understanding vaccine hesitancy around vaccines and vaccination from a global perspective: A systematic review of published literature, 2007–2012**. *Vaccine* 2014, **32**(19):2150-2159.
6. McLeroy KR, Bibeau D, Steckler A, Glanz K: **An ecological perspective on health promotion programs**. *Health Educ Q* 1988, **15**(4):351-377.

7. Michie S, van Stralen MM, West R: **The behaviour change wheel: a new method for characterising and designing behaviour change interventions**. *Implement Sci* 2011, **6**:42.
8. Wilson R, Paterson P, Larson HJ: **Strategies to improve maternal vaccination acceptance**. *BMC Public Health* 2019, **19**(1):342.
9. Wilson RJ, Paterson P, Jarrett C, Larson HJ: **Understanding factors influencing vaccination acceptance during pregnancy globally: A literature review**. *Vaccine* 2015, **33**(47):6420-6429.
10. O'Shea A, Cleary B, McEntee E, Barrett T, O'Carroll A, Drew R, O'Reilly F: **To vaccinate or not to vaccinate? Women's perception of vaccination in pregnancy: a qualitative study**. *BJGP Open* 2018, **2**(2):bjgpopen18X101457.
11. Wilcox CR, Bottrell K, Paterson P, Schulz WS, Vandrevala T, Larson HJ, Jones CE: **Influenza and pertussis vaccination in pregnancy: Portrayal in online media articles and perceptions of pregnant women and healthcare professionals**. *Vaccine* 2018, **36**(50):7625-7631.
12. Woolf K, McManus IC, Martin CA, Nellums LB, Guyatt AL, Melbourne C, Bryant L, Gogoi M, Wobi F, Al-Oraibi A *et al*: **Ethnic differences in SARS-CoV-2 vaccine hesitancy in United Kingdom healthcare workers: Results from the UK-REACH prospective nationwide cohort study**. *Lancet Reg Health Eur* 2021:100180.
13. Darwin Holmes AG: **Researcher Positionality - A Consideration of Its Influence and Place in Qualitative Research - A New Researcher Guide**. *Shanlax International Journal of Education* 2020, **8**(4):1-10.
14. Green J, Thorogood N: **Qualitative Methods for Health Research**, 2nd edn. London, UK: SAGE Publications Ltd; 2009.
15. Maxwell JA, Mittapalli K: **Realism as a Stance for Mixed Methods Research** In: *SAGE Handbook of Mixed Methods in Social & Behavioral Research*

Edited by Tashakkori A, Teddlie C, 2 edn. Thousand Oaks, California: SAGE Publications, Inc.; 2010.

16. Affun-Adegbulu C, Adegbulu O: **Decolonising Global (Public) Health: from Western universalism to Global pluriversalities**. *BMJ Global Health* 2020, **5**(8):e002947.
17. Stewart RE, Williams N, Byeon YV, Buttenheim A, Sridharan S, Zentgraf K, Jones DT, Hoskins K, Candon M, Beidas RS: **The clinician crowdsourcing challenge: using participatory design to seed implementation strategies**. *Implement Sci* 2019, **14**(1):63.
18. Tucker JD, Day S, Tang W, Bayus B: **Crowdsourcing in medical research: concepts and applications**. *PeerJ* 2019, **7**:e6762.
19. Wang C, Han L, Stein G, Day S, Bien-Gund C, Mathews A, Ong JJ, Zhao PZ, Wei SF, Walker J *et al*: **Crowdsourcing in health and medical research: a systematic review**. *Infect Dis Poverty* 2020, **9**(1):8.
20. Baum F, MacDougall C, Smith D: **Participatory action research**. *Journal of epidemiology and community health* 2006, **60**(10):854-857.
21. Melles M, Albayrak A, Goossens R: **Innovating health care: key characteristics of human-centered design**. *Int J Qual Health Care* 2021, **33**(Supplement_1):37-44.
22. Haigh F, Kemp L, Bazeley P, Haigh N: **Developing a critical realist informed framework to explain how the human rights and social determinants of health relationship works**. *BMC Public Health* 2019, **19**(1):1571.
23. Mercer J: **The challenges of insider research in educational institutions: wielding a double-edged sword and resolving delicate dilemmas**. *Oxford Review of Education* 2007, **33**(1):1-17.
24. Merton RK: **Insiders and Outsiders: A Chapter in the Sociology of Knowledge**. *American Journal of Sociology* 1972, **78**(1):9-47.
25. Ormston R: **The Foundations of Qualitative Research**. In: *Qualitative Research Practice - A Guide for Social Science Students and Researchers.* 2nd edn. Edited by Ritschi J, Lewis J, McNaughton Nicholls C, Ormston R: SAGE; 2013.

26. Okely J, Callaway H: **Anthropology and autobiography Participatory experience and embodied knowledge**, 1 edn: Routledge; 1992.
27. Abimbola O: **Being similar: other-identification during fieldwork**. *Anthropology Matters Journal* 2009, **11**(1).
28. Atkins L, Francis J, Islam R, O'Connor D, Patey A, Ivers N, Foy R, Duncan EM, Colquhoun H, Grimshaw JM *et al*: **A guide to using the Theoretical Domains Framework of behaviour change to investigate implementation problems**. *Implementation Science* 2017, **12**(1):77.
29. Braun V, Clarke V: **Using thematic analysis in psychology**. *Qualitative research in psychology* 2006, **3**(2):77-101.
