# Supplementary material for: “Figuring stuff out myself” – a qualitative study on maternal vaccination in socially and ethnically diverse areas in England
Source: BMC Public Health. 2023 Jul 21;23:1408. doi: 10.1186/s12889-023-16317-z (PMC10362695; doi:10.1186/s12889-023-16317-z)
Supplement: Supplementary file 3 — Additional file 3. [file 12889_2023_16317_MOESM3_ESM.pdf]

SUPPLEMENTARY INFORMATION FILE 3 – Topic guides, VIP-IDEAL study, 2022

VIP - IDEAL

a) Topic guide outline for interviews with pregnant/postpartum women

Introduction & Informed consent

- Welcome, making participant comfortable, reminding of purpose, reason for selection, reminding confidentiality, anonymised quotes in report, participant rights and other content of participant information sheet; no right or wrong answers; note keeping, audio-recording; questions bevor start of recording? Can stop/interrupt audio-recording or interview anytime.
- Informed consent (written consent or audio-recorded oral informed consent)
- Take note of socio-demographic info

Interview (audio-recorded)

General

- Can you tell me about your recent pregnancy?
- *If not yet mentioned*: Would you mind telling me about your experiences with the health care system since you have been/ while you were pregnant?

  [Follow-up questions/ probes regarding whether person felt well taken care of, listened to, whether there were any access issues to antenatal care, GPs etc, whether healthcare differed from previous pregnancy (if applicable) or from where the person lived before (if applicable) and whether any perceived impact of the Covid-19 pandemic.]

Key topics

- What do you think about having vaccinations during pregnancy?

  [Follow-up questions/ probes regarding maternal vaccine information sources, explore whether aware of different types of vaccines, and if so whether attitudes towards flu, whooping cough and Covid-19 vaccines differ, whether view towards vaccines has recently changed]

- Can you tell me about any experiences regarding any vaccinations you may have been offered by any healthcare workers?

  [Probe for by whom, when and how vaccines had been offered; timing of different types of vaccines (and boosters); explore decision making process; reasons for acceptance/ refusal/ uncertainty.]

- *If not yet covered:* Have you sought/ did you seek advice from anywhere or anyone (else) regarding vaccinations in pregnancy?

  [Probe from whom, for which vaccines, why, and whether it changed their views]

- What advice would you give a friend or family member if they asked you whether to accept any vaccines during pregnancy?

  [Probe for reasons and whether/why advice differs from own vaccination decision]

- What improvements do you think are necessary regarding vaccinations offered during pregnancy?

  [Probe for information needed to be able to make decision on current or new vaccines, preferred/most trusted information sources, healthcare worker behaviour, structures, processes, policies]

Acceptability of potential future interventions

- What would you think if …

  [Explore views/opinions regarding potential future interventions suggested during previous VIP-IDEAL study interviews, during PPI work and in relevant literature.]

Closing

Invite for questions or comments without recording; offer hand-out with advice and support organizations if needed; reiterate how data will be used; potential use of anonymised quotes; ask if can contact again in case of further questions/ help with analysis/interpretation of results, and/or dissemination of results and/or future related research; explain where results can be obtained; thank you and participant reimbursement (cash or gift voucher)

b) Topic guide for focus group discussion with pregnant/postpartum women

Opening/ Introduction & Informed consent

Welcome, reminding of purpose, reason for selection, role of facilitator (and note keeper), housekeeping and ground rules, respect, no right or wrong answers, reminding confidentiality, participant rights and other content of participant information sheet

Informed consent (written consent or audio-recorded oral informed consent)

Discussion (audio-recorded)

General

– Everyone to briefly introduce themselves, including name (preferably first name only, names will not be transcribed), how many months pregnant/ how old baby and other children (if applicable)?

– Could you please tell us about the healthcare you received during your pregnancy?

  [Follow-up questions/ probes regarding whether person felt well taken care of, listened to, whether there were any access issues to antenatal care, GPs etc, whether healthcare differed from previous pregnancy (if applicable) and whether any perceived impact of the Covid-19 pandemic.]

Key topics

– Can you tell me about your experiences regarding any vaccinations you may have been offered during your pregnancy?

  [Probe for by whom, when, how and which vaccines had been offered; timing of different types of vaccines (and boosters); explore decision making process, whether advice was sought from anywhere or anyone, including social media, and whether views have changed; reasons for acceptance/ refusal/ uncertainty]

– What advice would you give a friend or family member if they asked you whether to accept any vaccines during pregnancy?

  [Probe for reasons and whether/why advice differs from own vaccination decision]

– What improvements (if any) do you think are needed regarding how vaccines are offered to pregnant women?

  [Probe for information needed to be able to make decision on current or new vaccines, preferred/most trusted information sources, healthcare worker behaviour, structures, processes, policies]

Acceptability of potential future interventions

– What would you think if …

  [Explore views/opinions regarding potential future interventions suggested during previous VIP-IDEAL study interviews, during PPI work and in relevant literature.]

[Note: the final topic guide will include questions with sample probes and prompts]

Closing

Invite for questions or comments without recording; offer hand-out with advice and support organizations if needed; reiterate how data will be used; potential use of anonymised quotes; ask if can contact again in case of further questions/ help with analysis/interpretation of results, and/or dissemination of results and/or future related research; explain where results can be obtained; thank you and participant reimbursement (cash or gift voucher)

c) Topic guide for <u>interviews</u> with healthcare providers

Introduction & Informed consent

- Introduction, reminding of purpose, reason for selection, confidentiality, anonymised quotes in report, participant rights and other content of participant information sheet; no right or wrong answers; note keeping, audio-recording; questions bevor start of recording? Can stop/interrupt audio-recording or interview anytime.
- Informed consent (written consent or audio-recorded oral informed consent)
- Take note of socio-demographic info [Just to start, can you tell me a bit about yourself please? – then complete App6 table b) incl. age, gender, ethnicity/nationality, religion, education, current job title]

Interview (audio-recorded)

General

- Could you please tell me about your current role and any services you may provide to pregnant women?
- *If not yet mentioned*: Has your role changed in any way since the beginning of the Covid-19 pandemic? [If yes, probe how/why etc.]

Key topics

- *If not yet covered above*: How does your role relate to vaccinations in pregnancy?
- Could you tell me (a bit more) about how the services relating to vaccinations in pregnancy are organised at your clinic/organisation?

  [Probe for current practice, organisation and timing of different vaccines during course of pregnancy, and if it has changed during past few years]

- Could you please tell me about your views regarding vaccinations during pregnancy in general?

  [Probe for reasons, whether views differ for specific types of vaccines (flu, whooping cough and Covid-19 vaccines), and whether views have changed during the past few years, and if so, for reasons; probe for past infections]

- How do you usually approach the topic of vaccinations in pregnancy when speaking to pregnant women?

  [Probe whether they recommend any of the vaccines to their patients and reasons]

- Would you recommend any vaccinations to pregnant family members or friends?

[Probes/ follow-up questions for reasons and if it differs from recommendations to patients; if applicable probe under which circumstances (if any) they would recommend the vaccine?

- [If sufficient time: Imagine a new vaccine became available to pregnant women, under which circumstances would you recommend it?

  [Probe/ mention new vaccines currently under development, including vaccines against Covid-19 variants, but also Group B Streptococcus and Respiratory Syncytial virus, and what information would be needed in future for them to be able to make a decision on whether to recommend these new vaccines; how high the risk/severity of disease and vaccine efficacy would have to be, what policies and structures would need to be in place, etc.]

- What are the main sources of information available to you on vaccinations in pregnancy?

  [Probe for most trusted sources, and if/what additional information or training is needed]

- What do you think should be improved regarding vaccinations in pregnancy (if anything at all)?

  [Probe for suggestions for and opinions on possible improvements regarding how current and/or future vaccines are offered to pregnant women.]

Acceptability of current and potential future interventions

- Do you know about any current initiatives to improve the uptake of vaccination in pregnancy in South London or elsewhere? [Probe for details and opinions about these]
- *If not yet mentioned*: Have you ever heard about any …
- What would you think if …

  [Explore views/opinions regarding potential future interventions suggested during previous VIP-IDEAL study interviews, during PPI work and in relevant literature.]

[Prior to stopping recording, ask if participant would like to say anything else regarding vaccinations in pregnancy.]

Closing

Invite for questions or comments without recording; offer hand-out with advice and support organizations if needed; reiterate how data will be used; potential use of anonymised quotes; ask if can contact again in case of further questions/ help with analysis/interpretation of results, and/or dissemination of results and/or future related research and PPI work (indicate accordingly in table d of App6); explain where results can be obtained; thank you and participant reimbursement (cash or gift voucher)

d) Topic guide for <u>interviews</u> with other stakeholders

Introduction & Informed consent

- Introduction, reminding of purpose, reason for selection, confidentiality, anonymised quotes in report, participant rights and other content of participant information sheet; no right or wrong answers; note keeping, audio-recording; questions bevor start of recording? Can stop/interrupt audio-recording or interview anytime.
- Informed consent (written consent or audio-recorded oral informed consent)
- Take note of socio-demographic info

Interview (audio-recorded)

General

- Could you please tell me about your current role in your organization/institution?
- *If not yet mentioned*: Has your role changed in any way since the beginning of the Covid-19 pandemic? [If yes, probe how/why etc.]

Key topics

- Could you please tell me about your views regarding vaccinations during pregnancy?

  [Probe for reasons, whether views differ for specific types of vaccines (flu, whooping cough and Covid-19 vaccines), and whether views have changed during the past few years, and if so, for reasons; probe for past infections.]

- Would you recommend any vaccinations to pregnant family members or friends?

  [Probes/ follow-up questions for reasons if it differs from recommendations to patients; if applicable probe under which circumstances (if any) they would recommend the vaccine?

  If expresses concerns, ask what would allay these concerns; if unsure, probe what information would be needed to enable decision making about vaccines in pregnancy.]

- [If sufficient time: Imagine a new vaccine became available to pregnant women, under which circumstances would you recommend it?

  [Probe/ mention new vaccines currently under development, including vaccines against Covid-19 variants, but also Group B Streptococcus and Respiratory Syncytial virus, and what information would be needed in future for them to be able to make a decision on whether to recommend these new vaccines;]

- Which are the main sources of information available to you on vaccinations in pregnancy?

  [Probe for most trusted sources, and if/what additional information or training is needed]

- What influence if any, do you think your organization has on uptake of vaccinations during pregnancy.

- What (if anything) do you think should be improved regarding vaccinations in pregnancy?

- Do you know about any current initiatives to improve the uptake of vaccination in pregnancy in South London or elsewhere?

  [If yes, probe for details, and views on these initiatives.]

  [If interviewee thinks that improving uptake of vaccines in pregnancy is important ask questions regarding feasibility of potential future interventions suggested during previous VIP-IDEAL study interviews, during PPI work and in relevant literature.]

Closing

Invite for questions or comments without recording; offer hand-out with advice and support organizations if needed; reiterate how data will be used; potential use of anonymised quotes; ask if can contact again in case of further questions/ help with analysis/interpretation of results, and/or dissemination of results and/or future related research and PPI work; explain where results can be obtained; thank you and participant reimbursement (cash or gift voucher)
