# Supplementary material for: “Figuring stuff out myself” – a qualitative study on maternal vaccination in socially and ethnically diverse areas in England
Source: BMC Public Health. 2023 Jul 21;23:1408. doi: 10.1186/s12889-023-16317-z (PMC10362695; doi:10.1186/s12889-023-16317-z)
Supplement: Supplementary file 4 — Additional file 4. [file 12889_2023_16317_MOESM4_ESM.pdf]

SUPPLEMENTARY FILE 4 – Further exemplary quotes, VIP-IDEAL, 2022

This file includes additional anonymised data extracts illustrating (sub-)themes.

[Acronyms behind quotes in parenthesis: W=pregnant/post-partum woman, M=Midwife, GP=General practitioner, Ph=Pharmacist/Pharmacy manager, O=other service provider; All quotes are from interviews, unless FGD (Focus Group Discussion) is indicated.]

Contents

Structural and organisational factors 2

Organisation of and access to ANC 2

ANC booking visit 4

Subsequent ANC visits 5

Access to influenza and pertussis vaccines within maternity and at GP clinics 6

Access to Covid vaccines within maternity and via vaccination centres 8

Access to maternal vaccines via pharmacies 9

Resources, Roles & Responsibilities 11

Health Information System (HIS) and Apps 13

Behavioural factors 15

Passive versus active decision-making process 15

Interaction with HCP/ provider recommendations 16

Engagement with information material and social media 18

Interaction with family, friends & others 20

Individual characteristics and influences on vaccination decisions 21

Risk-benefit perceptions 21

Knowledge and skills 26

Emotions and Trust 31

Cultural norms, philosophy and beliefs 36

Attitude towards mandatory vaccines 38

Participant recommendations 40

Structural and organisational factors

Organisation of and access to ANC

Limited GP involvement due to new midwife-led ANC services and access via new self-referral system

"In years gone by the woman would, when she knew that she was pregnant she would go to her GP and then the GP would refer the woman into the hospital that she wants to give birth in, so the women has a choice as to what hospital they want to deliver in, then that the GP used to refer them to that hospital, but nowadays in the last few years women can self-refer to the maternity service via the website, so they log onto the website of the hospital that they choose, complete a self-referral form, and then they will be contacted with an appointment." (M)

"sometimes people will come and see us when they first are pregnant to ask advice about referral process into antenatal care and if they've got any other medical conditions during pregnancy they'll see the GP, but generally in [*anonymized borough name]* we don't provide any antenatal care. But in [*anonymized borough name*] if people are booked at [*anonymized hospital name*], normally they would do a shared care for antenatal so that we, the patients see the midwife for some of the antenatal visits and they will see the GP for some of the antenatal visits." (GP)

"the thing is you can self-refer yourself to the hospital, you have all your antenatal clinics at the hospital and then you have a baby and this whole time the GP doesn't know you are pregnant." (GP)

"Yeah, so the beginning, to be honest, that was probably the most complicated area […] I had to make a self-referral. […] Mainly that my doctor's receptionist didn't know where to go to and they weren't clear with what directions to give. So, once I'd done that and worked it out, there was also, like it was, like you submitted your information but it didn't give you a timeline of how long it would then take to get back to you, and that was, I think it ended up being like six weeks or something, so that just, I don't know whether it's gone through, you know, that was the bit that was a little bit uncertain." (W)

"So I Googled it, contact a GP, contacted them and they said, oh, come in and get a form, that you then need to send off to [*anonymized hospital's name*]. So I went in, collected the form, filled it out and scanned it to this email address that they told me to send it to. Then I got a reply from [*anonymized hospital's name*] saying, oh, we don't accept paper forms any more, you should tell your GP service, like you should tell your GP practice we don't do that any more, you need to fill in this online form instead. It was like, well, surely it's your job to tell my GP service, not mine." (W)

"I did the registration, … where you make a self-referral for your pregnancy, and then I think you indicate which hospital that you wanted to give birth in, and then that's it. […] I think it was straightforward, but I don't know if it's because of the fact that I kind of knew what I was doing [as it was her second pregnancy], or just because of the fact that it was easy to find, I mean the form is easy to find, you just kind of Google it and it comes up." (W)

"we have about six to seven hubs in the community where we see women and provide antenatal care, and actually postnatal care as well. […] They are located in children's centres, and health centres, we used to be located in GP surgery many years ago but because there's limited space for midwives to practice from, they only give us like one room per week, but we prefer to work more closely together with other teams like health visitors and with other midwives, so we now are in children's centre or health centres where they give us two to three rooms Monday to Saturday." (M)

Delayed access/ disrupted ANC

“I registered my pregnancy thing with my GP, I got I think an email also saying that I’ll be contacted by the CCG or something, it was my first time so I didn’t know who was supposed to contact me or whatnot so it was my friend who was telling me that you are supposed to have a scan by now and I had to call my GP, my GP said someone was going to call me back, they called back apologising, they said I was lost in the system. So it looks like if I hadn’t called, no one would have followed that and they were saying it was because of the Covid and staff shortages.” (W)

“Yes, first I want to share with you my situation is, in this country, is I’m overstay so I believe that I couldn’t have access to the medical service. […] So I believe I don’t have right to a medical service. At the beginning I believed that so I was very worried because I was pregnant, but a friend of mine explained me that everyone have free access for the healthcare and I receive a very good attention in the hospital. All my checks up were very good, I receive a very good attention in this country.” (W)

“So I was living in, that’s my first temporary accommodation they give me in [*anonymised South London Borough*]. [A*nonymised South London Borough*] give my temporary accommodation in East London, so I was there till I give birth to [*anonymised*], so the second temporary accommodation, because they say I have to move, I have to move from East London to North London, I’d say a couple of months before they sent me back here [to South London].” (W)

Organisational challenges and changes due to the Covid-19 pandemic

“it has changed [due to the pandemic], as in how we deliver our care, and also the schedule of care was disrupted, so some things were moved to virtual or telephone consultations, and some things remained face-to-face, so definitely there has been some changes, but not one thing, like a big change.” (M)

“because of Covid that’s been virtual, So yeah, 16 weeks virtual appointment is not ideal because for them it feels like a really long time that they haven’t seen a midwife and it can be quite sort of lonely for them in that time, so yeah, doing it over the phone is not ideal. […] Before the pandemic it would have been, they would have been face-to-face for their first appointment before 10 weeks and face-to-face for their 16 week appointment, so that changed, that changed with the pandemic, so yes, sometimes people can not see a midwife until 28 weeks.” (M)

“I don’t think anybody planned for the pandemic and all the restrictions associated with it, so we changed quite significantly as a result of Covid, you know, a lot of our NHS providers for at least a quarter of the year were redeployed elsewhere so those services came to a standstill, so for example, our team of midwives who deliver continuity of care they were gone, so we had paused, all those women were cared for by other midwives because of the complexities involved with redeployment.” (O)

“Yeah, so the goal of continuity is that the woman only sees a few midwives in her pregnancy […] they're only seeing a few midwives and building a really, relationship and trust with the midwife. […] Yeah, so continuity is kind of the goal for the NHS service, but I think it was, unfortunately, put on hold with Covid, but the, in terms of difficult, I think it's difficult for the system to accommodate, but, actually, in terms of satisfaction for both the midwife and the women, we see there's higher satisfaction in, you know, like I prefer working in this way and I know that the women also prefer having this type of care model. […] I think the challenges are mostly staffing...” (M)

"women [prioritised for continuity of care] are those who are considered to be socially vulnerable, so that can be a range of things from mental illness, asylum seeker or refugee status, domestic violence, social care involvement and sometimes it can be poor obstetric history. So someone who's had a number of miscarriages. And […] those of black and other ethnic minority groups, so at least 51% of [continuity care] caseload are from one of those ethnicities, and that's to reduce the health inequalities which we know currently exist within maternity." (M)

"We [continuity of care service] were suspended for at least 6 months during 2019, yeah the service was suspended because of staffing and the hospital was not considered to be safe and the care we were providing was not a priority at that point. Yeah, so we were redeployed into working in the hospital. I think the problem was essentially there wasn't enough staff to provide safe maternity care without us. So the idea was that the service needed to be safe before we, you know, I think continuity of care in a pandemic was considered a luxury and it wasn't essential." (M)

"So, women who are under what are called traditional teams, so non-continuity of care, they may during their antenatal care see a different midwife each time they go for an appointment." (M)

"I never had the same midwife, but I think maybe it's Covid or maybe an English system […], just because I thought it was nice to have a continuity of one person, like they always see you and then they know exactly what, you know, what you have, instead of always asking you the same questions and not knowing how your body is, so I found that a little bit hard, quite impersonal, but I think that's also because of the lack of personnel in the NHS…" (W)

"I saw the different midwife during my pregnancy. […] I had a, I think 7 or 8 midwives I saw during my pregnancy. It's not good for me, I don't like that." (W)

"I saw a different midwife every time for my appointment, so I didn't really have any consistent relationship with a midwife. […] It was just frustrating that it was always a different midwife..." (W)

ANC booking visit

"She [the midwife] mentioned in the first visit, she mentioned everything that will be done, all the scans, all the vaccinations, and blood tests, she mentioned everything. But you know, I can't keep hold of everything in my head on the first visit. […] And this is the time that I've been vomiting, tiredness and everything. It should've made sense if it was, if there was a leaflet for me to remember. Okay, oh at this stage in pregnancy I need this, at this stage in pregnancy I need to do this. There was nothing like that." (W)

"it's really difficult because you get an hour and 15 minutes to do that whole appointment and obviously you want to go into a lot of depth but I think you end up, unless they're asking more questions, I think you end up with your headline of what you say to everyone. […] So it really like boils it down to sort of 20 seconds of talking [about maternal vaccines at booking]..." (M)

"It wasn't on my first appointment [*that the midwife mentioned the vaccines*], I can't remember when exactly but it wasn't on my first appointment. […] I think not even the second time, maybe the third time, maybe the third time of going for antenatal, I think it was then they raised it, yeah." (W)

“in my notes, in my antenatal notes there’s a big A4 piece of paper about the Covid vaccine that says, “Do not get it, it’s not safe in pregnancy,” and then, but at my booking […] the midwife did sort of say, “I know this is in your notes but have you thought about getting it because the Government are now starting to say der-der-der-der-der?” If it’d been me I would’ve been like, right this needs to come out. […] Yeah, it was bizarre, it was like, and like for me it didn’t make a difference, I was vaccinated, but I can really understand why other women were conflicted if that’s what was happening in every hospital up and down the country.” (W)

Note: See also quotes on ‘reduced trust and/or confusion due to change of guidance’ in ‘Access to Covid vaccines within maternity and via vaccination centres’ section above and in ‘Emotions and Trust’ section below

Subsequent ANC visits

“So I always make sure that I mention [the vaccinations] at booking, so they’ve been given that information, and then I give them the written information, and then to be honest because there is so much I’ve got to fit in I don’t always mention it, because there’s so many things, and then the ladies have got their own questions, so unless they’re asking me then I don’t necessarily raise it. … Yeah, so I mean in this 20 minute slot I’ve got to do baseline observations, blood pressure, dip sticking urine, get the lady on the bed for an abdominal examination, measure her bump, feel the baby, listen to the baby’s heartbeat, draw bloods, then I’ve got to put everything onto the computer … I think that yeah it’s definitely time and then priorities, and I think that would depend on midwife-to-midwife, because you might have some midwives that really prioritise this and spend maybe five minutes talking about it and then others that don’t.” (M)

“So booking appointment we tell them which vaccines we’ll offer. 16 week appointment over the phone we remind them of those vaccines and then when they go to their 20 week scan usually there is a whooping cough vaccine clinic running, so they should be offered it there and then at 20 weeks and get it while they’re already in and then following that, just following up to see, oh did you have that, did you have the vaccine when you went in? So that’s the kind of main interactions that I would have with people about vaccines other than if they bring it up and they ask because they’re unsure of it and they want advice.” (M)

“There’s posters up in clinics so when they come for scan and then they’ve got one, two, three, four, five, about six midwife appointments [if taking place at hospital rather than within the community] where they’ll walk past the drop-in clinic for the vaccines, so there’s ample opportunity for, you know, for this to be…” (M)

“Maybe yes, maybe no [regarding whether pregnant clients will be reminded about the vaccines at their week 20 appointment]. If we have the midwife support workers in the reception and they are our core midwives support workers which they know how the system works sometimes to remind them “any woman here who come in for scan, vaccines are there”, sometimes they remind them, sometimes they don’t. We have posters but…[…] The thing is that usually woman are bombarded by [various] posters. You come to Antenatal Clinic you don’t know where to look and you go to toilet and the doors are like full.“ (M)

Access to influenza and pertussis vaccines within maternity and at GP clinics

Access to influenza and pertussis vaccine within maternity

"I had to ask myself okay so when can I take the whooping cough, […] it was more like you have to remember next time you come to ask for it. So if I forgot, or if I didn't know, I probably would have left without having the, the vaccine." (W)

"Maybe one thing that I find was difficult to grasp is who to call and what to do, so I was told that I would get those vaccines but I wasn't told what to do in order to, you know, do I need to call someone to book it in or, and at my 12 week scan that I was then told, you know, in passing by the sonographer to then go you know, next door and make sure I wait two doors down to get my vaccine, otherwise I would have just left and waited for a phone call…" (W)

"Actually I'm not sure how they do it. I guess they've got posters up and I think sometimes whoever's doing the vaccine clinic kind of goes looking for customers and tries to catch people but I don't know, yeah, maybe it's an idea to kind of have a poster in the scan room or let the sonographers know that it's happening." (M)

"And we also in the vaccine clinic give women their MAT B1 forms which are their forms for their employer so they do sort of have to come to us *[maternal vaccination clinic*] anyway which helps… […] They are notified during their booking appointment that they will be offered the vaccines and what they are and why they're offered to them so they should be told then. They also have a 16-week appointment, with Covid that's a telephone appointment and again they should be mentioning you know, next time you see anyone it'll be the scan and you'll also be offered these vaccines, but I do have quite a few people come to the drop-in clinic and I say, "Oh do you know why you're here?" and they say, "No," say, "Oh, has anyone spoken to you about the vaccines?" "No," and you sort of have to start from scratch. But we do have posters all around the antenatal clinic saying basically are you 16 to 32 weeks pregnant and have you had the whooping cough vaccine, and then signposting people to where they can have it. […] I think some of the time the women have forgotten, they get so much information in their first appointment that, and they don't get given any kind of paper copy of the information they've been given so I think it's relatively easy to forget anyone's spoken to you about it. But I think also midwives probably miss it as well when they're giving out so much information, I think language barriers can be a bit difficult as well" (M)

"when I went to my whooping cough vaccine which I'm pretty sure a midwife mentioned or told me to get and I could just pop in and have it, walk in after my 20 week scan maybe, you could just pop around the corner and have it, super quick, very easy." (W)

"I think it needs to be more accessible around work, because you're already taking quite a lot of appointments off, it was quite difficult to be out of work so much." (W)

Access to influenza and pertussis via GP versus maternity and recent changes

"[*In the past, when GPs were still more involved in ANC and pregnancy was coded in GP systems*] we run like flu clinics and it literally, it's often outside office hours, so it's been on Saturday morning, and you literally just you're all there. You've literally got, each person has a one-minute appointment, or something, or a two-minute appointment […] you do the consent, you do like, you know, a mini risk assessment, stab and go. […]." (GP)

"So, we (community continuity of care team) don't do the vaccinations ourselves in clinic we refer them to either seek it from their GP or [*anonymized hospital name*] do a walk-in vaccination clinic which runs in the hospital. So they can attend that as well." (M)

"The GP [they] can access for whooping cough, […], they're quite familiar with it, smaller, small, community-based, parking is, sometimes accessibility's better, parking is better, they know where they're going, the hospital can sometimes be quite a daunting place, get lost, you know. […] a lot of our clients will access the practice nurse service for the vaccines which tends to be a little bit easier to obtain then an actual GP appointment" (M)

"I know at the moment that the GP surgeries are under a lot of pressure, I mean running Covid clinics, catching up on children's vaccinations that weren't done during lockdown and things so I think if there's things that are felt necessarily to antenatal care, and especially now that we don't always know that patients are pregnant, and also you know for a lot of my patients they don't really understand or maybe English isn't their first language and you know it's difficult for them, so I think if there's things that are kind of recommended in terms of the health of the, as part of the pregnancy care probably that's better done in a, in the antenatal setting in the hospital just to kind of streamline it and make it easier. Because otherwise if people are, you know it can be difficult to get appointments at the GPs or the nurse is away and then it's delayed and then that might potentially have implications for… But I guess it's also making it what's easiest for the mother just to access." (GP)

"women are, the ones that can access their surgery and get an appointment are having the vaccine through the surgeries, but the ones that are finding it difficult, which most women find it difficult to get an appointment nowadays through their surgery, are finding it a lot easier because we ask, we have women asking questions all the time about when they can come and have their vaccinations." (M)

"Service users were finding it difficult to get an appointment via their GP surgery's practice nurse, to get a vaccination nurse, appointment for their whooping cough and flu. […]. So we now have two vaccination clinics per week […] so we have the vaccination nurse come in and offering whooping cough and flu vaccines [*at vaccination clinics in hospitals*]." (M)

"it [*the pertussis vaccine*] was first provided by GPs, then there was always a conversation about it should be the midwives giving it […] So we start with a clinic […] we had problems with rooms, we didn't have space, clinical space to facilitate that. […] We didn't have staff for it as such. […] So, uh, Covid happened and things changed […] So yeah, what happened, GPs closed, there was no option of whooping cough vaccine from them, a lot of the primary care fell on hospitals, not just vaccinations, a lot of things, so we facilitate one room to provide these vaccines… So most we reduce the appointments for the pregnant woman to face-to-face appointments to the absolutely necessary ones to avoid contact, […] so it was basically agreed at 20 weeks when they come for the anomaly scan it was we tried to offer all. We still do like that, we offer their scan, we offer the vaccines, we offer the maternity certificate which has to be given out from 20 weeks, and we did also observations, […]. And then now we are going back to normal, primary care GPs are open, they can provide vaccines again, but obviously this service run very well, increase the intake to vaccines and woman's demanded, it was very popular and it was working very well. We are happy to give it and also from the Public Health England it said that we should, the maternity services they should provide these Vaccination Clinic within maternity, so the clinic should be, the placement should be within maternity services. The staffing doesn't need to come from maternity services" (M)

"So anywhere that will sort of offer the flu jab, because they're pregnant, should give it to them, so either through their GP or wherever the clinics might be, it should be, but it's one that they have to

go and do and be proactive with as well. […] I think, having the clinic for the whooping cough vaccine is probably the main reason that women get it, I think without that they just, they wouldn't because if you have to, if you have to call your GP for anything, that's enough to put you off even if you, you know, you've got a real significant problem because GP services are so stretched at the minute and so hard to get hold of" (M)

Opportunistic vaccination

"I think, I think the key is that like opportunistic, let's get them while they're here and give them a vaccine." (M)

"it was, with the nurse, yeah, I was like, you know, I'm feeling sick, is there anything I can take, and it was like, oh, we can give you anti-sickness tablets, and she was like, oh, while you're here you might as well, when you're leaving you might as well book in for, because you'll be back. Yeah, so … I just booked it while I was there. " (W)

"we may see a patient for a medical problem during pregnancy but because we've got the ten-minute slot I guess we wouldn't always have the, you know think, oh this lady's 26 weeks maybe I should check whether she's had her vaccinations or not." (GP)

"I think because when it comes to like flu season, we've always been encouraged like, give opportunistic jabs, even if the patient, like the patient is in your room, and they come up as they're eligible, give it to them there and then, and so you should be like Oh, Have you had your flu vaccine this year? […] It's a lot easier when you're doing it with like people that have chronic health issues because they they probably had it before, but when it's pregnant women they're usually fit young, healthy, and they've never had the flu jab before, so it. It gets a bit more complicated or longer anyway." (GP)

Access to Covid vaccines within maternity and via vaccination centres

Temporary access to Covid vaccines in maternity in hospital (in different South London Boroughs)

"I think at the height of the pandemic, when, or when the vaccines came out we had nurses that came from the vaccination clinic to set up a makeshift, like a pop-up vaccination to offer the Covid jabs and I think this was so that the pregnant women didn't have to go and join the main queue downstairs in the marquee as it were, so we had two separate clinics running on the same floor which was quite confusing." (M)

"So there was a pilot of providing Covid vaccine here in Antenatal Clinic. […] They took another room, even if we were struggling with rooms, they provided Covid vaccine there. There was a nurse and an admin person, they have their fridges, everything. […] no one went there, women don't want a Covid vaccine […] so it didn't really work." (M)

"At one point we were offering Covid vaccines, but the uptake wasn't that great in terms of how long you can have the vaccines out, so, but we also attached to a mass Covid Vaccination Centre within the hospital, so women are signposted just down the corridor to have their Covid vaccines." (M)

Access to Covid-vaccines at vaccination centres (no special arrangements for pregnant women)

"…they were urging vulnerable people to get the vaccine, and but we weren't particularly contacted to get it any quicker and, you know, pregnant women still had to queue for like an hour to go and get their vaccine which when you're pregnant and you have to go to the toilet about every 20 minutes, it was not realistic. […] I remember I had an appointment, I looked at the queue [at the vaccination center] and I was like, oh my gosh, I can't wait this long, so I didn't even join the queue, I was like I'm just going to book it at another odd time or something, and then I returned and it was still…" (W)

"some of the questions [*that clients are asked when arriving at Covid vaccination centre*], I would say, doesn't help, because on the, when you go and have your Covid vaccine it's are you pregnant? So that immediately rings, you know, makes people feel like, oh, should I be having it, do you know what I mean? But it's, what they don't realise is, it's aTrust

data collection question, just so they know the amount of women, so I tend to ask, tell women that question, that question is not a trick question, it's not that it's not safe in pregnancy, it's because we want to keep, we want to keep an eye, or we want to know the amount of women that have had vaccination whilst pregnant, it's just there for data collection. Whereas the general public don't know that, they just think, oh, why are you asking me?" (M)

Reduced trust/confusion about change of guidance

"I felt like that advice was still mixed and confused because I was speaking to friends who were maybe two or three months ahead of me in their pregnancies. And they said that they had made the decision to go along and get their first vaccines, but once they got there, when they got asked are you pregnant they then were told oh wait, we need to read this information and they said that their reactions that they were getting from the people at the vaccination centres was enough to sort of them put them off for them to then go back home and reconsider […] It was after April, but still the reactions, they were getting still at the vaccination centres would, it was just not and yeah completely clear." (W)

"some of the questions (asked at the vaccination centre), I would say, doesn't help, because on the, when you go and have your Covid vaccine it's are you pregnant? So that immediately rings, you know, makes people feel like, oh, should I be having it, do you know what I mean? But it's, what they don't realise is, it's a data collection question, just so they know the amount of women, so I tend to ask, tell women that question, that question is not a trick question, it's not that it's not safe in pregnancy, it's because we want to keep, we want to keep an eye, or we want to know the amount of women that have had vaccination whilst pregnant, it's just there for data collection. Whereas the general public don't know that, they just think, oh, why are you asking me?" (M)

Note: Further quotes on this theme in 'Emotions and Trust' section below

Access to maternal vaccines via pharmacies

"no vaccine should be done through GP practice at all, […], it's a waste of time and that should be done through pharmacies because they can basically walk in at any time to get it done, so if you booked an appointment at the GP practice, you wake up in the morning and you're not feeling very well, right, you're not going to get a vaccine done, you've got a temperature, you're not going to get a vaccine done so then you have to cancel that appointment and rebook it, now if no-one else takes that appointment it gets wasted. […] we offer both service *[drop-in and by appointment]*, […]

especially pregnant women should be going to a pharmacy first to ask any questions because they are there to provide healthcare advice, they know what they're doing and they're the ones you should be talking to, whether it's vaccine-related or not […] if it's anything more serious then the pharmacy will escalate it to a GP practice or to 111 or even to urgent care or A&E or whatever it is but most of the time, 99% of the stuff can be dealt with in the pharmacy." (Ph)

"…the flu vaccination the pharmacists are doing and it has increased some uptake, some people who would not have the opportunity to have it, because here it's, you don't, in most pharmacies you don't have, you don't book an appointment, you just walk in and it doesn't take long, then you have it so it's good, it's increasing uptake, it's increasing uptake." (Ph)

"I used to work in a pharmacy so I'm a big fan of pharmacies, so yeah, and I sort of know that pharmacies... […] if they [pregnant clients] don't believe us they can ask a pharmacist, but yeah, certainly pharmacists have definitely got a role to play, for sure. […] Pharmacists, if I know the pharmacists are like working, you know, with vaccinations. I wouldn't necessarily ask a high street pharmacists [questions relating to Covid-19 vaccines in pregnancy] they wouldn't necessarily, you know, have the most up to date information, they usually do but you can't guarantee it so, you know, with access to hospital staff I think you've got a bigger pool of talent you can draw on haven't you really." (O)

"I know they [pharmacists] do a lot of the flu vaccinations, they've done that for quite a number of years. I guess the main thing with the pharmacy is you know have you got the facilities there if some, you know if somebody faints or has an allergic reaction of that kind of thing? Because obviously if you have an allergic reaction and you're pregnant and your, so you know your blood supply is for two people not one then that could be reasonably significant, so… […] I think probably the pharmacists have to pre-screen certain people anyway I think to be allowed to complete the vaccination within their, the way that they can prescribe it so, [pause] yes. But I mean I know there's a lot of encouragement for more vaccinations to take place in pharmacies and things just for convenience and to try and increase uptake and things, which you know it does seem sensible" (GP)

"And I think that the area that I'm working in now is quite diverse and I've noticed that this group of clients, it's far easier to direct them to the GP surgeries because they're very familiar with that and it's very easy, I don't know if they would feel as comfortable into walking into Boots for example and saying you know, oh can I have my vaccine? I think they feel there's a, more structure to going to their GP practice that they're familiar with, yeah." (M)

"As long, as long as that information [on maternal vaccinations by pharmacists] is then passed on to whoever it needs you passed on to so ideally to be passed on to the GP, um, and then, if the midwife wants it, they can access it from the GP. But the information needs to be readily available. […] I think actually, if you do get other healthcare providers involved in vaccines, it does take a lot of pressure off primary care. Um, it allows us to focus on things that needs doing. I think that way, if we do if we do that, I think we need to invest more like the admin and the so the whole infrastructure needs reinvesting." (GP)

"we were made a priority in my opinion far too late, I mean I was in a massive, massive, massive queue for the booster vaccine when I was eight and a half months pregnant, like out in the cold in December and like someone literally… the pharmacist literally came out and like escorted me to the front, like, "Please come to the front of the queue,[…] but just there wasn't really thought given to like the comfort of people who might be struggling, not just pregnant women, but I mean like there wasn't a bathroom there and things like that, it's like if people are having to go to the loo every hour and you have to queue for two hours they're not going to go, and but if there is ever a mass vaccination like

that again if you want pregnant women to be there make sure there's a bathroom, make sure they don't have to wait for two hours on their feet, like just basic stuff like that could make a difference." (W, FGD)

Resources, Roles & Responsibilities

Resources challenges in maternity services (staffing, space, equipment, material & supply)

"… the pressures and the staffing crisis that we were having because people were asked to isolate for two weeks, which it then fell to those who were in to work a lot more." (M)

"…in community, there's a lot of sickness and a lot of burnout with the midwives, so a lot of the time you are not going where you think you're going to go that day, so you might think you're on home visits one day but actually someone's off sick so you have to go and cover a clinic and that clinic might be over an hour from where you live […], so if you're one of the ones who can do anything, you tend to be the one that gets moved to go and cover wherever on a given day." (M)

"And the other problem is the actual setting, the actual physical clinic, we don't have a room as such for it. […] So if [*anonymized, others*] need the room … they take out our posters and put theirs, which is fair because then we can't run the Vaccine Clinic there or at least during times. So whoever the vaccine is, the midwife is coming back to do the vaccine again, the clinic again, sometimes they forget to put the posters back again. […]." (M)

"The other thing that could potentially increase the uptake is if the midwives can all do their vaccination training. I mean, it would be difficult in the community clinics to know how much stock you would need of each and for there not to be wastage and things but if, you know, if they are there for a 28 week appointment and you say, oh do you want to have your flu jab, yeah, I'll do it for you, then yeah. […] But I know that that would be really difficult to make possible in the community, to have the right amount of stock and to have fridges and to make sure that the midwives are able to give vaccines and etc but ideal world it would be well you're here for your midwife appointment, do you want a flu jab, yeah, I'll give it to you now." (M)

"I don't know about, the Covid vaccine I think is a lot more difficult because of the storage, storing is a bit of a nightmare." (M)

"I ended up not having the whooping cough vaccine, and not for not want of trying. I called [anonymized hospital], which was my place to go and get it, three or four times. […] I got told by my midwife it's a walk-in service, so I went to [anonymized hospital], one day, …, they said, oh, no, we've run out of the vaccine, you'll have to come back another time. So I thought, oh, that's a pain. I called a couple more times over the next two or three weeks, in advance, so like just to check you've got the vaccine. Oh, no, we don't have any today, or the clinic closes at one … I'm only free in the morning, or I'm only free in the afternoon,…" (W)

Resources challenges in pharmacies

"a lot of pharmacies fall down [*cannot provide Covid-19 vaccines*] because they don't have two or more consultation rooms and the other thing is, not having two pharmacists in place is another thing that's different. […] It's not really the pharmacy's fault, it's more the Government in that side of thing

because the funding isn't there. So pharmacies had funding cuts for the last, since June, for the last 7 years they've had it cut every year, cut. So now we're looking, at the moment we're about 12% lower than we were 5 years ago and that's real term cuts. […]

some pharmacies have the space but they're just not busy enough to have the two consultation rooms and you think if they make that kind of investment, unless they're more or less guaranteed to get the service then they'll make this room up but not being used and then it's just there's an empty space and it's not generating any income […] If there was a contract actually given to pharmacies, like this is your contract, we're going to give you this service but to provide this service you have to have x, y and z in place and then they say right, if you get all these things in place then you would definitely be providing the service then pharmacies would be like, okay, cool, I'm getting that, I know I'll be getting that money to provide the service so then I can make the change in the pharmacy to accommodate that and then as soon as that's done someone comes and checks it and yeah, it's all done, start providing. […]

So pharmacies have fridges for all the vaccines anyway because we provide fridge products, so we have inhalers, we have eye drops, we have creams, you know, all in the fridge anyway […] no-one actually has the capability of storing vaccines at -20 or -30, whatever degrees it is, no pharmacy in the land is going to have that, no GP practice is going to have that, it's all going to be done centrally, at central depots and then it gets thawed and sent out to the pharmacies and then you have x number of weeks to actually use that up." (Ph)

Vaccination-related roles and responsibilities of different providers

"Though I think actually, if you do get other healthcare providers [such as pharmacists] involved in vaccines, it does take a lot of pressure off primary care. Um, it allows us to focus on things that needs doing. I think that way, if we do if we do that, I think we need to invest more in like the admin and the, so the whole infrastructure needs reinvesting." (GP)

"*Interviewer:* In general, what is your opinion, do you think GPs should be more or less involved in vaccinations in general and vaccinations in pregnancy specifically? *Interviewee:* No, I think it works the way it is. I think it is, from my perspective on what I've seen, I think it works fine the way it is. There is a lot of information out at booking appointments. But then, when you come back, you get that particular information again. So I do feel there is enough information and education for women to make that decision themselves, and they are then followed up by midwives to see if they've made that decision. If there are any queries they will tackle them. So I don't think GPs, GPs per see need to be any more involved. I do feel that sometimes we don't know enough about vaccines though." (GP)

"I know at the moment that the GP surgeries are under a lot of pressure, I mean running Covid clinics, catching up on children's vaccinations that weren't done during lockdown and things so I think if there's things that are felt necessarily to antenatal care, and especially now that we don't always know that patients are pregnant, and also you know for a lot of my patients they don't really understand or maybe English isn't their first language and you know it's difficult for them, so I think if there's things that are kind of recommended in terms of the health of the, as part of the pregnancy care probably that's better done in a, in the antenatal setting in the hospital just to kind of streamline it and make it easier. Because otherwise if people are, you know it can be difficult to get appointments at the GPs or the nurse is away and then it's delayed and then that might potentially have implications for… But I guess it's also making it what's easiest for the mother just to access." (GP)

"The thing is that we are midwives and our priority is midwifery, not vaccinations, and we have, I keep saying it, we have so many things to go through as a midwife so that's not our priority and it's not our

field. And okay, we understand it's great for woman that they have all together, you can give it to them, that's it, and yeah we understand we have to give the information but we don't have time to discuss with them." (M)

"Well I was asked, I was asked if I wanted to do that [get training for vaccination clinic], but I declined on the basis that I don't feel that that's a role of a midwife, I would much rather be in clinic rolling up my sleeves, you know, helping women than sitting in a clinic waiting for people to drop in to give vaccinations, because I feel that that's more like a nurse's role, than midwives, I think it's a waste of resources." (M)

Health Information System (HIS) and Apps

Insufficient information transfer between providers and shortcomings of electronic health records, referral systems & documentation

"I think the issue with pregnant women is that they're not necessarily coded on the GP system, if you've got diabetes, it will be coded and it will be coded for life, whereas if you're pregnant it comes and goes and like I know many GPs don't like coding it because it then doesn't come off so you end up being pregnant forever. […] the thing is you can self-refer yourself to the hospital, you have all your antenatal clinics at the hospital and then you have a baby and this whole time the GP doesn't know you are pregnant." (GP)

"they can self-refer so they don't actually need any sort of GP interaction and the communication isn't always very good so we don't necessarily get a letter to say you know your patient's had their booking visit, their baby's due now, and if it's not we, unless the patient tells us we don't actually have any record of that pregnancy on our, you know we have no way of recording it on our system. […] I think it would be really sensible for it to be on the record because for, in terms of things like prescribing you know there'll be lots of people who may be on certain medications which you know should be stopped in pregnancy." (GP)

"it's definitely something that's been raised a lot by sort of all the clinicians that I've spoken to is 'we need a link with GPs, we need a link with primary care' […] I think especially with maternity, ideally the GP would know that the woman was pregnant but a lot of the time they don't and I think there's a gap there somewhere to be honest that probably needs to be looked at." (O)

"it's asked [*prompt in online system to ask clients if they have been vaccinated*] at the booking and we're made aware of these things, but it's not a mandatory field later on at any other points during the sessions to ever mention it again. […] and then to be honest because there is so much I've got to fit in I don't always mention it…." (M)

"that booking appointment that I mentioned has, on our system it has prompts to go through the different vaccinations in pregnancy and talk about them at that first appointment. It's not a mandatory field on that computer system. The main ones are, yeah, that we talk about the Covid vaccine and then the flu jab and whooping cough. […] I tend to talk a lot and sort of go through that with people and I have a list of things to talk about at 16 weeks but I know sometimes you'll look on someone's records and the documentation will say, you know, 16 week phone call done, no concerns. So I kind of go through a massive list of things but I don't think everyone necessarily does..." (M)

"I think there's a section [in the electronic health record system] where you can, let me, I'm trying to look at it now, there is a section, vaccinations, vaccinations plus, so you could document if they'd had a vaccination but it's not widely used. *Interviewer:* And what do you think, why it's not widely used? *Interviewee:* I don't know actually, I think because it's, I think it's quite new to the system and it's not really been advertised. I mean only now when you're asking me about it have I looked and seen that you can put it on. I don't think it's something that people know you can do." (M)

Lack of user-friendliness of apps and not used to access vaccine information

"*Interviewer*: Has the midwife explained to you before how to use the app and what's in there, or…? *Interviewee:* No, I don't think they know themselves because sometimes I see them struggling to use it and they don't know what's on actually, what information's on there themselves, so I feel like there maybe hasn't been enough training…" (W)

"It was like it was made in 2005 and the world has progressed and app development has progressed, and the app has not, or it's like they did it on a massively tight budget. I just think the user experience is so bad." (W)

"in the app, I got information week-by-week about my pregnancy, so every week you could log in and say you're at week 14, here's what should be happening in your body, dah, dah, dah, so that was quite handy, but I find that the midwives, maybe because they're not trained enough to use the app but they weren't logging things into my [anonymised] app so I had to basically still book things in my personal diary and I would open it and it would be sometimes some appointments would be in, but it wouldn't say what it is, so my 20 week scan would just show up appointment, and I didn't know what it was, but I knew because I noted it down. […] it was not used in its optimal way, yeah, the app. I think it's a really great app but yeah it's the way it's used probably is not…" (W)

"there was a lot of information on that app and it wasn't easy to find" (W)

"…certainly no-one at the hospital seemed to have any access to that [*the info in the app*] as far as I'm aware, and I felt like you needed to say that, tell each person what you wanted to do regardless of what you'd put in the app, so I'm not really sure what it was for. […] I'd prefer the app as a means of communication but I think needs to be better linked.

[…] There is a little information section on the app where you can sort of go digging through to find, but there might be something in there, but I… certainly nothing would, I don't remember anything popping up and saying have you thought about a vaccine, so if there is there might be information buried, but I don't know." (W, FGD)

"I'm not very good with apps, I just didn't find it that user-friendly, every time I'd forgotten my login, or my code." (W, FGD)

"I never used it like, it's tiring, you know? *Interviewer:* Tiring to use the app, in what way tiring? *Interviewee:* You know when you are pregnant, like we go through a lot when we are pregnant, you know, you become lazy, you become tired, so these apps it's tiring, you know, like literally it is tiring, so if you take information, good information and keep it in the app and I think that every pregnant woman go there, you know, not all of us go there. […] It's tiring, plus social media, you know, we have a lot now in our phones to look at, you know, mm. […] From your check-up, instead of getting inside and reading the notes and what you get into your phone you start watching your social media, you know?" (W)

Information for participants via hardcopy material versus app (and digital exclusion)

“one had to sign up [*to the App*], which I think about 70% to 80% of our women do now, and then they, yeah, they opt in for push notifications […] The numbers that have declined have been very low, there is definitely a gap between the ones who consent to sign up and never do, and then there’s ones who do just decline...” (M)

“like the only thing with paper notes you’ve got to lug them everywhere, like if you go away for the weekend you take your paper notes with you just in case. But I liked it because I could read it and I was like, let’s see what they’re saying about me [laughs]” (W)

“Yeah, I was lucky because I was in the hospital so much, they were able to give me the leaflets […] Yeah, I think … for me it was easier to read a physical thing and sort of come back to it and put it to one side and, you know, highlight or mark any bits that I was unsure of, and then take it back and say, oh, could you just explain this bit, or whatever?“ (W)

“I prefer hard copy because some people, not everybody has access to internet and not everybody that can use the computer. But when you give me hard copy like this, I can easily just pick the paper and go through it, then I know what to do.” (W)

“I’m sorry, I’m not good at internet. […] Mm, I don’t think I have anything paper. We always sit down and discuss.” (W)

Note: Please see ‘Knowledge and skills’ section and ‘Participant recommendations’ section below for quotes regarding language skills/ barriers.

Further quotes regarding ‘Health information systems and apps’ in ‘Participant recommendations’ section below

Behavioural factors

Passive versus active decision-making process

“To be honest I wasn’t even sure if you have to take it [*pertussis vaccine*] or not. They offered it and they say, because for the first time when I was pregnant with my first child I thought it’s, I have to take it that’s why. So, I didn’t, I didn’t know it’s an option so that’s, they just gave me an appointment that I have to get this whooping cough so I went. And now, with the second one I said, “Okay the first one got it so let’s get it for the second one as well, let’s not make any difference,” that’s why. […] It’s, like I said it’s not like I have to take, they don’t, they are not going to force you. If you decide, no I don’t want it, I’m sure they say, “Okay, that’s your decision.” But like, but they just mention like, “It’s for your child.” You know, they explained why it’s there and stuff, so I just, I just sort of thought, okay let’s do this for the child. So that’s why and that’s why I took it with the second one as well.” (W)

“I’m thinking back about the confusing messaging around pregnancy, at first it was don’t do it, then it was… […] so I really understand why [Covid-19] vaccine uptake has been relatively low in pregnant women and of course that varies within ethnic groups as well. So I get it, you know, I understand the reluctance for people that might not have as much capacity to dig around and find out if it’s safe or not.” (O)

"I always thought that when I asked a question, I didn't get a very good answer, so then I had to do my own research." (W)

"I don't think midwives or GPs were knowledgeable to answer like questions that I had, they will just give sort of a blanket response to say "oh yeah, check the website and, you know, do your own research", as opposed to going deep into some of the other queries that I had. So it was more blanket responses." (W)

"it's left for you to, to actually read about it, research about it if it's actually good for you. So it's left for, left for us. Some people can be like, oh it's good for me, I'll have it, some will say no, let me read about it, let me research about it and see if it's actually good, and that's how it meant to be. If you, you don't just say, oh it's good, oh okay I'll take it, you know I told you I saw a specialist doctor, she recommended I take Aspirin for headache, but I'm a nurse, I was thinking, why would I take a blood thinning medication for headache? I didn't take it, …" (W)

"you make your research, […] you can actually just ask randomly, random people who are pregnant or who have given birth, oh did you take this, did you take that... […] So asking your doctor, your consultant, your GP, your midwife and probably friends and family who, friends and family who are pregnant or who have given birth before, their experience and what they think about it and you can also read online, yeah a lot of articles online." (W)

"No just I don't know if it's because the GPs and the midwives don't have that thorough information [about Covid vaccine] themselves either. […] I had to do my own research on it. (W)

"She [*her mother*] trusts the healthcare system. I trust the healthcare system but I'm also someone who would try to do more research if they were to be like "oh you should take this". I wouldn't just be, you know, it's the word gullible but just be like yes straightaway." (W)

"I would say do your own research, make sure you're comfortable with what the side-effects or what the implications could be. I don't think I would really push them [*other pregnant women*] to take anything if I'm honest because I think it's your body, it's your child, so you have to do your own research and make sure that you're comfortable with whatever you're putting in your body." (W)

Please also see quotes regarding 'Importance of not putting pressure on women, and vaccination as personal decision' in 'Interaction with HCP/ provider recommendations' section below.

Please also see opt-out versus opt-in approach in 'Participant recommendations' section below.

Interaction with HCP/ provider recommendations

Recommendation against Covid-19 vaccine or no clear recommendation or information

"So I know also somebody, she's like also family but she works in hospital with the babies and she knows lots of midwives, so we spoke about it as well and she said most of the midwife doesn't, they don't recommend it as well…" (W)

"I had my first midwife appointment in [*anonymized, a few months after UK government recommended Covid-19 vaccines to pregnant women*], and the Covid vaccine I asked specifically about,

I mean I was already of the intent to get it via the midwife, and she was very cautious in her answer, saying there was no data, you know, no research that it should be safe, but really it's you know, it's entirely down to my decision that, you know, […] no further information than that, and again really not encouraging me as such, just kind of authorising me if anything […] I already had the intention of getting it, but it did shake my confidence a bit, […] that is the voice of the NHS kind of telling you it's your choice, it should be fine, but you know, can't say anything. That did shake me a bit, I still got it afterwards, but it means I delayed getting it." (W, FGD)

"I said, well, just don't want it [*Covid-19 vaccine*] yet because I just don't know what the impact could be, or the effects could be, and I think one of them probably said, yeah, I agree, and maybe another one said, right, fair enough." (W, baby born ≥6 months after UK government recommended Covid-19 vaccines for pregnant women)

"I just felt like it was very throwaway..., so I just felt like again they've ticked off their box, they've mentioned the whooping cough" (W)

"they mostly talk about the whooping cough and the flu vaccine and then they do mention the Covid but kind of not as, it doesn't seem as priority. I think it sounds like something that they just have to say. And then when I say I haven't had it, they don't really say anything further, so there's not really the chance to have that conversation." (W)

The only one that they kept pushing was the Covid vaccine, that's the one that they kept saying to get […] you haven't had the Covid vaccine, we do recommend you getting it done, and then I would just say no, and then they'd just say, okay, and that was literally the conversation each time. […] They never really asked, they literally, as soon as you would say sort of no, that would be the end of the conversation, and that was it, really." (W)

"I feel that once someone has expressed to me that they are going to decline the recommendation I don't push it because one complaint that a lot of people have is that they don't feel that their wish is respected and that we're badgering them and I don't want to do that so… […] If people say, "Oh I don't want the Covid vaccine." I never ask them why, once I've given them the information that's fine. And I think people don't, they don't, I don't know if it's fear of judgement or I don't know. But I find a lot of people, they don't really want to talk about vaccines. " (M)

No clear recommendation or no information/dialogue regarding pertussis and influenza vaccines

"I remember whooping cough they just sort of was like, "By the way, at around 16 weeks go and get your vaccine from the GP," and I think because like […], it wasn't like you should, like advice or anything given about it, it was just like, oh by the way, like a fleeting comment…" (W)

"nobody approached me for the whooping cough or for the flu, I actually proactively had to ask and approach them" (W, FGD)

"…once the whooping cough injection time came, she asked me if I had the injection and I said no. *Interviewer:* And then, what did she say? *Interviewee:* Nothing [laughs], literally nothing. *Interviewer:* How did you feel then when she said nothing? *Interviewee:* That I felt I didn't actually need to get it done, and I felt like jumping on the rooftop and saying to people, don't get your whooping cough because you don't actually need to get it done [laughs]." (W)

"No, they didn't, actually, they just kind of say that we recommend getting it, but, no, I don't actually remember them saying the sort of benefits to it. […] the flu vaccine, they weren't, they kind of asked me once and I said no, and that was about it." (W)

Importance of not putting pressure on women and vaccination as personal decision

"vaccination is kind of like what information can I give you to be empowered to make your own choice rather than I think you should have this so I'm going to convince you […]. Because a lot of midwifery is about empowering people, you know, because they're not, they're not unwell, they're not poorly, it's not, you know, your blood pressure's high so this is the best medication, a lot of it is what do you want to do, this is your body and your baby and your choice, so I think maybe we were slightly different from nursing in that sense, that we're so used to, you know, always trying not to be convincing someone but you're empowering them and giving them the information to make their own choices, so maybe there was a slight difference from nurses" (M)

"The midwife doesn't need to compromise to force, doesn't need to go in depth, they just offer information, 'this is why we advise and we advise it because of this and this is the evidence, you do whatever you want'. That is basic information and the woman doesn't feel too pressured to say anything" (M)

"we can underestimate the amount of influence that we have as a doctor and I don't know whether it sort of, you know I think shared decision-making where people feel comfortable with their own decision is better. […] I think it has to be an individual decision as well you know for women to make in terms of what they feel the risks are to themselves and their babies in terms of vaccination. Because even if you know the evidence says that it's the, it's better to do, if they have some sort of negative outcome from the pregnancy and they felt that they were pressured into making a decision they may feel that the negative outcome was due to the vaccination even if it may be unrelated and so I think it has to be a, you know that it's better just to give people the information and then allow them to make the decision." (GP)

Unambiguous recommendation as facilitator to vaccine uptake

"…it was like the normal thing, like it prevents the baby from whooping cough, it prevents the mother and baby from whooping cough so not like, they were like, oh, they didn't give me like a 50/50 percentage or I'm 50% sure I'm negative or positive, like they were 100% positive about it like oh this is okay and so I was fine with it." (W)

Note: Please see 'knowledge and skills' section below for further quotes on unambiguous recommendations from HCPs committed to relay relevant information and have honest dialogue regarding maternal vaccines, including Covid-19 vaccines

Engagement with information material and social media

Hardcopy or electronic leaflets

"Yeah, I was lucky because I was in the hospital so much, they were able to give me the leaflets, but I do believe that they had them electronically as well, for people who weren't going in." (W)

“we’ve moved away a lot from giving paper information, but to me women still find it useful having handheld information, a lot of things are now electronic, all our leaflets, everything are online, they have to log onto their [anonymised maternity app] in order to access the leaflets, and the information, and if you’re not IT savvy they don’t always, they’re not always able to access those information’s.” (M)

“No, I haven’t [received any vaccination-related information material], the only thing I got is a piece of paper after the whooping cough one, just to say about the possible side-effects, of what to look out for. “ (W)

“there was nothing in there to say at this stage of pregnancy you do this. There was information leaflets like, if you have an emergency, if you’re bleeding, blah, blah, she gave that, and then I think she gave something else as well, I can’t remember what that was. And she gave the hard copy, but there wasn’t a reminder, there was something, oh the blood glucose tests, that one she had already booked it that day, so I knew I was going for something on this day, I think this was, this was like four months in advance she had booked it. […] so I knew it was already in my calendar, I’m going somewhere on this day, but that whooping cough I didn’t even remember, I didn’t even know, they didn’t ask, there was nothing. Yeah and I missed it, I didn’t have it. […] There was a leaflet in there. […] Not on vaccination, not on vaccination, it was just a leaflet on, if you have an emergency, if you have a bleeding, that’s what was in there.” (W)

Searching online/ via social media

I did my own research, so I would go and look for like blogs, like real-life experiences from woman who’ve taken it and yeah […] Some were negative, some were positive. Of course I would just kind of go with how I feel. I would just go with how I feel and just kind of combine the experiences together, instead of just going for one. […] I’ll Google it … I’ll just take, first of all I’ll just type in like the name of the vaccine and I’ll type in like people who have taken the name of the vaccine and then a bunch of like, a list of like different sites will come up, and then I will just see like articles from women’s blogs, sites, and yeah.” (W)

“So there was this particular lady [*in her women’s forum*], she has five kids so she would document, so for the past five pregnancies she’s documented it and yeah, so hers was very interesting. […] and she was actually having complications with her pregnancy at that time and she didn’t actually take the whooping cough vaccination and I believe it was down to the fact that she just said that because it’s fairly new she doesn’t know this reliable so she would rather just do her research, intensive research on that rather than just taking what her midwife would say.” (W)

“on twitter… there were a few who said they would just not have it [*the vaccine*] because it’s too risky for your unborn child and they feel like having immunity from having Covid is better” (W)

“I also joined a pregnant women’s forum, and then there was lots of conversations about the Covid jabs […] It was Baby Centre. […] you could join, and you could speak to other mums who are due to give birth around the same time as you.” (W)

“I go on this app called the Peanut app […] And it’s a mummies community because I don’t have many mummy friends around me,[…] someone saying that “oh, you know, this vaccination or this medication gave my”, was the cause to, that contributed sorry towards, you know, their child being maybe disabled or, you know, just have some sort of disability. So yeah, that would kind of like just worry me.” (W)

Please see ‘Health information system and apps’ section above for quotes on hardcopy information material versus apps.

Interaction with family, friends & others

“when I spoke to my mum about it, who is actually, who gets the flu vaccine, she just thought I was a bit too young to get that” (W)

“And my mum, she was … just like oh back in her time they didn’t offer that, so I guess that’s the new like guidelines […] she was just like “you’ve done your own research and you feel like you want to take it then why not? And if they’re advising you to take it then I don’t see why”, she puts her trust in them so she was just like, you know, she would trust them not to … give me something that is not good for the baby or good for myself. So I just took that and I just went.” (W)

“I can feel that sometimes some groups of the ethnicities they, or their culture, their family, they don’t want it, I’ve seen that. […] They are “we don’t do it, my family never done it, we don’t do it in my family, no, I’ve never seen it done”. They refer to the family which is their culture. That’s their comment. That is why it’s culture thing.” (M)

“I speak to [anonymised relative/friend] and [anonymised relative/friend] who’s a pharmacist, because they’re very much against it and [anonymised relative/friend] has like fertility issues already so she said she’s basically not trying to have anything else that would worsen that, so everything was really around fertility and that’s the main conversation that I’ve had with people, it seems to be centred around that.” (W)

“my partner have a friend, a friend who got pregnant and they got the flu vaccine, so the wife it didn’t take her well, you know, so when I told my partner about it as well, he’s the one that discouraged me as well because he told me that, ‘Oh my friend the wife got that vaccine, that vaccine is not good.’ ” (W)

“I think maybe there just could be some statistics to show maybe the levels of people taking it within certain Boroughs potentially or yeah, maybe just allowing people to know, you know, other people are taking it so it’s not that bad. I think not knowing who’s taking it and not, like low levels of uptake with the vaccinations might put people off. Maybe sharing statistics of, yeah, how many people in each Borough are taking it or how many people in each area are taking it might help, maybe more media coverage because I think maybe people want to know that they’re not alone and that it’s okay.” (W)

“I remember always when I went to the hospital for my check ups I found other mums and I spoke with them about the vaccines and asked me didn’t want take any vaccines. […] They told me that they don’t take any vaccine because you can affect your baby, to your baby. *Interviewer*: So do you think that had an effect on your decision or…? *Interviewee:* Yes because they didn’t take, I believe maybe they are right so it’s better, so maybe it’s invasive. […] Yes, was in, at [anonymised hospital name] when I had a scan, first scan. In the waiting room there are some other mums waiting and they [vaccination clinic staff] offered the vaccine. They told us you don’t need to wait, you don’t need an appointment, you can take it in this moment and they [the other pregnant women] didn’t want. […] Yes, yeah, someone say everybody in the room you can take it if you want, come at this room to take the vaccine. […] if you want take it, go to the room and in the room they explain more. […] and nobody goes to this room. […] they start talking, I no, I don’t want to take this vaccine, no, me too….” (W)

"There is one lady again I met at the GP, she was complaining about it as well, so because I didn't want to start with, when I heard all those things, now they made me to completely not take it, especially the flu one, that's the one that I heard stories about. […] She was just at the waiting area. […] In fact she was asking me if I had already taken the flu vaccine, because when I'm pregnant I have to take it, I say no, she said [baby making noises] she was talking about her experience but it was not good for her…." (W)

Individual characteristics and influences on vaccination decisions

Risk-benefit perceptions

Risk benefit evaluation

"I just thought the risk for reward wasn't worth it for me in terms of, I didn't need it." (W)

"Most mums don't want the flu vaccine, a big majority, and the most common comment I hear is "I get worse side-effects from the vaccine than from the infection itself"." (M)

"All in all, it's, I would say, safer to get them than not to get them [*Covid and flu vaccine*], because you don't want to catch either whilst pregnant." (W)

"…you know on balance I would probably still opt to have the vaccination because of the risks of not having it outweigh the risks of possible side effects." (M)

Perceived high risk of vaccine on baby

"I just wasn't sure that vaccine, and because people were having blood clots at that time, so I'm like, no, not when I'm pregnant." (M)

"my biggest fear like prior to getting pregnant was, say, for example I get the Covid vaccine and then in five years' time they then realise there's a side-effect and it like affects the baby or like it affects fertility?" (W)

"I personally wouldn't do it just due to, just some things that I've seen in the media. I haven't necessarily done a lot of research on it but things that I've seen in the media about women miscarrying due to having the vaccination. … it happened very quickly to sort of solve the issue you might say of Covid and I don't know how much I trust that with my unborn child. So, it's a risk that maybe I would take for my own self because of sort of my system, not my immune system but my own body, but not for an unborn child who is still very dependent on me whilst growing inside me." (W)

Perceived risk of vaccine side effects expressed by single mothers

"I don't want to risk anything because I'm the only one around so I don't want anything that will give me complications and everything." (W)

"So this is why I'm scared to take vaccine, yeah, because I don't want to fall sick for my son. If I fell sick, example, who would take care of this boy? Nobody." (W)

Note: See box 14 (after 'emotions and trust' section) for further quotes regarding fear of negative vaccine effects

Perception of pregnancy as vulnerability

"…but when you're pregnant you have so many, you know, you feel vulnerable … I think there's this underestimation of how, like I'm creating this little being and it hasn't been easy, so I'm not just going to take any risk blindly, I want to find out about things, I want to be reassured, and I find that there was maybe not enough of that, reassurance." (W)

"I think when you are pregnant you do naturally feel more vulnerable and so you, I don't know about other women but I felt I automatically want to know what I can do to look after myself and my baby and I do feel like the campaign for the flu vaccine was targeted towards not one particular group but several groups but you know people that were vulnerable." (W)

Lack of perception of pregnancy as vulnerability

"Yeah, they always say pregnant women are vulnerable, but to me, I'm not vulnerable, I'm still myself. I'm a low risk, I'm not part of the high risk birth, whatever, I'm low risk, I'm fine, I'm healthy, I'm eating, I exercise, I go to work, I do everything, so I'm fine. I just want, …, I am not having it [Covid vaccine] when I'm pregnant, that's it, no. […] I was never vulnerable. […] Yeah because they always, so with pregnant women they graded them low risk, moderate risk, high risk. […] So I'm the lowest, so that meant there's no risk at all." (W, herself HCP)

"I'd see some people on Instagram that I knew that were like, thank God women now have access, pregnant women are now considered a vulnerable category and we finally have access to the vaccine and amazing, and I was thinking, oh, God, like I don't want, like I'd not even thought about how dare we not be categorised as vulnerable, I was thinking, I'm not vulnerable at all, I'm fine, and then you saw other mums who felt the same as me, like they just don't know enough yet, and a lot of people saying, I do want to take it, but after I have the baby." (W, refused all vaccines)

"with flu and with Covid they were so, so sick, especially the unvaccinated ones, they were already vulnerable. I think the difficulty with pregnancy is that these women are generally fit and healthy and they don't understand that this does make them, like being pregnant made them vulnerable to Covid, especially, they didn't, and even to flu, much more vulnerable […] but generally it would just be the same thing, like we advise that as a pregnant person that you're more vulnerable to, like more susceptible to, and advise that we have a vaccine for it, essentially. Essentially it's this conversation that you are more vulnerable, this is on offer, it's seasonal…" (W)

"although, yes, pregnant women did fall into a vulnerable category, the majority of women who are pregnant are not sick, they are pregnant" (M)

Perceived risk in case of underlying health conditions or complications

"If I had severe asthma or I was obese or I had Type 2 diabetes, I would have taken it [the vaccine]." (W)

"So if I had like high blood pressure, diabetes or just problems carrying a pregnancy for a certain amount of time, then yeah, that would be the health issues that I would consider not taking the vaccine." (W)

“I had too much nausea and vomiting, it was so much vomit. So I can’t have vaccine with my health and they said OK. […] Maybe it would add more to my health stuff so I didn’t take it.” (W)

“So I can’t take it, for me, because I don't know what will happen. Because I’m always careful. Because I have a series of miscarriages, I don't want anything that will affect me when I’m pregnant.” (W)

“I think, again, underlying medical conditions, people who are more susceptible to it may have a stronger reaction to having the vaccine, … but then again, they are also the group who are probably more likely to benefit from having the vaccine, so that’s where it’s very hard to weigh it up and that’s why I think it really had to be a personal decision for every single person.” (M)

“I didn’t think about it because, maybe because during my pregnancy I was mostly cranky, not just myself, not very excited most of the time and you know coupled with the fact that I was having some pains so I didn’t just want to inflict more pain…” (W)

Perceived risk of vaccine in case of allergy:

“when you live with an allergy you always feel a little bit vulnerable because it can kind of crop up from nowhere.” (M)

“we’ve had a few people saying oh, I’ve got allergy to, I’ve got a nut allergy I can’t have the vaccine, so it’s got nothing to do with nuts, you know, that kind of thing. Even one of my staff actually has got quite severe nut allergies and he’s like, I can’t get the vaccine because I’ve got a nut allergy. I said no, you can have the vaccine done, it’s got nothing to do with nuts.” (Ph)

“I think with any vaccination there is a risk of anaphylaxis, and I think pregnant women are immunocompromised, and so I think that extra caution needs to be given… it was very worrying to me when they took away that 15 minute wait after giving the vaccines, […], so say that this 15 minutes is being taken away in order to get more people vaccinated, but that was in turn putting more people at risk.” (M)

Perceived risk and individual biological differences

“Our immune system is not the same, you know, sometimes when we are having an underlying condition, then when I receive that vaccine then it will make me sick or dizzy or what… […] our bodies are different, you never know what will happen by then, because on me sometimes it can mislead us, like I had that, that for the Covid vaccine, I felt no need to take the Covid vaccine and I ended up being a patient of Covid.” (W)

“For example, my system, my system is not the same. That person might take the vaccine, she’s fine, another person would take the vaccine, there is issue. So it’s different. … It’s different systems. Everybody has their own.” (W)

“I know some people say, you know, “but I’ve taken it and I’m fine” but everyone has different reactions…” (W)

“everyone is affected differently as well, like you say someone could have three vaccines and during the nine months and be fine, but other people who say what if I’m a sensitive one and I have a bad reaction” (W, FGD)

Influence of experiences or decisions during previous pregnancy

“with the second one I said, "Okay the first one got it so let's get it for the second one as well, let's not make any difference," that's why.” (W)

“I wasn’t comfortable with it, you know, yes from my two babies [born in home country] I never got that kind of vaccination before, like my two pregnancies, so I didn’t, I was not comfortable in taking it. […] The thing is it differs, you know, the first pregnancy we come too excited, you know, you come eager to learn about a lot of things, you know, that one it can trigger a pregnant woman to be taking the vaccine, but only when you’re having your third child, your fourth child it’s quite difficult, you know, because yes, that’s the journey you have gone through before…” (W)

Perception of low risk of disease/ low susceptibility to infection (based on experience)

“I had the Covid and I didn’t have a vaccine and my symptoms was very mild, so I think my body is not, you know, my, yeah, it’s not too bad. So for now it just needs to be treated like another flu or something.“ (W)

“Flu, I know in myself I don't have flu so why am I going to take flu vaccine? So I will not take it, no, no, I won’t. […] But I know I’m healthy, I don't have flu so how come my baby will have flu? So I will not take it.” (W)

Perceived high risk of disease (based on experience)

“I had a feeling that it will have negativity in my baby, that’s why I didn’t take the Covid one. But at later stage I took it and I was still breastfeeding her, I decided to take it, to take the Covid vaccine. *Interviewer:* So what made you change your mind then…? *Interviewee:* The seriousness of Covid now, the seriousness of Covid, my husband got Covid, my partner, he got Covid and he passed it to me, then I could see that this is serious, this is serious. […] I could see that this sickness is serious, because the same one I didn’t want to take the flu one, I never thought that it is important to me, because I don’t get flu like that, but now I see the importance of like if it happened I get pregnant, I will take it, why because I see the seriousness.” (W)

Less motivation to recommend if HCP has no experience with vaccine-preventable disease

“just off my minimal experience of the ladies I see in my [*community*] clinic, there wasn’t a huge difference in outcomes between the ladies at the beginning of the pandemic where majority were unvaccinated and those towards the end of the pandemic where majority were vaccinated.” (M)

Greater motivation to recommend if HCPs has experience with vaccine-preventable disease

“The midwife there bless her she basically went to go into a spiel about please get the Covid vaccine, and she was so relieved when she found out that I’d already had it, because she was like, “Oh my God, thank goodness, I don’t have to give you the spiel,” because she’d been working in the ward and she’d seen so many pregnant women admitted, being really, really poorly with Covid and she was basically just desperate to… she wanted to get as many people vaccinated as possible. So I thought it was completely different midwife experience, ones in the community were very nervous about recommending it, and then on the flipside ones that had been working in the hospital with some very sick women, pregnant with Covid, she was desperate for me to get vaccinated. So it’s just within the NHS, within the same system you had vastly different opinions and experiences which yeah was interesting.” (W, FGD)

“Yeah, I’m very pro [vaccine]. I’ve, I think from looking after, like me looking after women who [Pauses] you know, who have had Covid during their pregnancy and how difficult that became and how anxious they were about, you know, about baby and everything like that.” (M)

“we have certainly seen women and birthing people become incredibly unwell as a result of not having you know a Covid vaccine, and yeah it’s the risk to mum, baby, it’s really quite sad […], I would definitely recommend women to have it and yeah.” (M)

“Well just look at what’s going on, what was going on, people were like dying from it, who haven’t been vaccinated, we’ve had loads of women that have got, come into ITU seriously ill with it, and in comparison, women who had the vaccine weren’t as seriously ill as those who didn’t have it, that were looked after in intensive care, so it’s giving people the facts of the likelihood, you know, what the likelihood is, so and giving them the, you know, the knowledge of what had been found out so far, I think that’s what convinced quite a few women saying, you know, out of just giving example, five women that had Covid three of them, or four of them were in intensive care, and those four didn’t have the vaccine, where the one who did didn’t, wasn’t as seriously ill.” (M)

I think it was a good idea [*Covid vaccinations in pregnancy*], I know people who have died…” (Ph)

Benefit for baby versus pregnant person

“I’ve actually saw like a tiny baby with whooping cough once and so the, really you know the vaccination is more for the protection of the new-born baby rather than the mum and so sometimes that’s easier to explain.” (GP)

“Yeah, so I think, I think with the whooping cough vaccine it’s because it’s mainly it could pass on that immunity to the baby so, you know, they’ll say, …, that’s when they’re quite motivated for it is because it’s passing on the immunity to the baby.” (M)

“With the flu vaccine I feel more women decline it because they’ve never had it before, it’s for their protection, it’s not for the baby…” (M)

“whooping cough, obviously at that stage I took that one up, and they were really good in explaining that it wouldn’t just have an impact for me, it would cover the baby for those first few weeks, until they got their inoculations.” (W)

Perceived low benefit/ effectiveness of vaccine

“I don’t really see like the main sort of benefit of the Covid vaccine because it doesn’t prevent you from getting it and you can still sort of spread it on, and a lot of the people that I know that have been vaccinated have actually had worse symptoms than like me and my friends, who haven’t been vaccinated. I don’t know, it could be also an age thing as well, because it’s a lot of my mum’s friends that have had the Covid vaccine that have been pretty unwell, whereas sort of when me and my friends have got it, like I had it literally like a month ago and I literally had a cough and that was literally about it. “ (W)

Evaluation of risk based on exposure

“she didn’t get any of the vaccines when she was pregnant because the flu and the whooping cough, it was during Covid, so she didn’t see anyone, so she didn’t think it was necessary for her to vaccine her baby because when the baby was born, she didn’t, wasn’t in contact with anyone, so, whereas like

we had talked about it and then she had told me, oh, yeah, I recommend doing the whooping cough because of my nephew, who is at nursery, and then I have other friends who also have babies and who also, you know, get sick and stuff. So then I didn't want, I understood, yeah, that's the reason why I got the whooping cough…" (W)

Alternative risk-mitigating behaviour of women who refused Covid-19 vaccine during pregnancy

"I was taking all the pre-measures, I was wearing the mask, washing my hand, and I was still working …, I was a [anonymised frontline worker], yeah, but still I didn't have it [the Covid vaccine]. My health and safety and the safety of the baby comes first, so I was like no I'm not having it [the vaccine]." (W)

"I don't want to take the vaccine so always I protect myself. … I'm really, really, really, really scared of Covid so I don't want that. So always have my sanitiser. I have a big bottle of sanitizer…" (W)

Covid was quite bad, so I was still worried, you know, to get it when I was pregnant. But I was like what can you do? Just, yeah, protect with the mask, clean your hands and that's it, yeah." (W)

"I didn't want to share the news I was pregnant yet yeah so. For that reason, we had to sort of tell people that I was unwell for for various other reasons and that's why we couldn't come to a wedding, for example. But the real reason was I didn't want to catch Covid and I knew, especially having not had any vaccinations, I was more potentially at risk of getting it in a more serious way." (W)

Knowledge and skills

Conflicting information

"I don't know if it's because I'm scared because there's not enough information or it's I'm scared because there's too much information, that I think it's, no, I think it's there's too much information that contradicts each other all the time, and so I think that's what makes me not want to do it [get the Covid vaccine]…" (W)

Misconceptions and general health knowledge

"The flu jab is kind of, I think a lot of people have this idea that if you have the flu jab you will get the flu and a lot of people who have had it once and been ill afterwards say, oh I'm never going to get the flu jab again…" (M)

"Because I've heard lots of like negative things about the flu vaccine, and I know that it injects you with the flu" (W)

"Yeah, having those conversations with them about the differences between live vaccines and not-live vaccines. Asking them what, you know, your relative, how was the vaccine administered? It was an injection, OK, well, it's not going to be a live vaccine then, it couldn't have caused it." (M)

"Yeah, well, I had, we were having one discussion with a midwife […] She [*the midwife*] was like, "how can they make this vaccine so quickly when they haven't even cured cancer." I was like, because it's so different. And yeah, just that sheer misunderstanding and again was still touting that thing about well, you know, they've proven it that Covid was created in a lab in China and it was because somebody ate a bat. And it was like, "what are you saying?" (M)

“I think it’s quite an uphill battle sometimes countering this misinformation, isn’t it, it's so widespread with the internet and you know, WhatsApp and text messages and TikTok and all this sort of stuff, you know, it’s, yeah, it’s hard to fight it sometimes...” (O)

“Nobody spoke to me about vaccination. I think I never had vaccine before. The only vaccine that I had when I go to the A&E, they want to check, they take my blood, they check.” (W)

“The whooping cough, I don’t know the difference between the flu vaccine and the whooping cough, I don’t even know, it’s just something to do with flu, both of them, I don’t really remember which one were they talking about” (W)

Lack of awareness and information about vaccines

“And also the whooping cough, just because I had never heard of it before. So I had never heard of this whooping cough before, so it wasn’t something that I was familiar with, so I didn’t really think it was very necessary” (W)

“All people was speaking about the Coronavirus but whooping cough, I never heard this name so I less important about this.” (W)

“*Interviewer:* Have you ever heard about the whooping cough vaccine at all? *Interviewee:* Yes, but the whooping cough is for children, it’s not for adult. My baby has it, they gave it to my baby.“ (W)

“the whooping cough vaccine’s been quite long established so there’s lots of leaflets, there’s lots of stuff online. I think the trouble with the Covid vaccine, it’s a new thing isn’t it and for every bit of information that was coming out in the press and on the news and, you know, Chris Whitty doing his nightly talks and things, there were people shouting the other side saying it’s all a hoax, it’s all rubbish.” (O)

“So from that experience with [anonymized community engagement programme], what really was kind of obvious, it was the lack of factual information in the community that was relayed by Public Health officials, by the NHS staff and so on, so I’ve heard about women, pregnant women, not really knowing if it’s safe for them to take [Covid] vaccination or they weren’t even offered the vaccination because they were pregnant.” (O)

“in the end they, it’s about them [pregnant women] being able to make a judgement call for themselves but you can only do that when you’ve got the available information.” (GP)

Ability or opportunity to ask HCPs questions

“If I’m honest with you I didn’t [ask questions about maternal vaccination] just because of, uh… Because I kind of like, their energy was just very boom, boom, boom, boom, boom. […] Even though she did say “oh do you have anything?” It was just sort of like “okay, I’m just saying this just to say that I’ve said it but, like to seem nice but really I don’t want to answer your questions, I don’t have time if you have”, you know, and I know that’s assuming but it did feel like that because, you know, once I came out of the office and they’re just like “next person” […] I just noticed all my appointments were just very, very quick, like everyone’s just coming in and out, in and out. So that’s why I did make that assumption that maybe I wouldn’t have, really and truly I don’t think they would want to listen to whatever I had to say, yeah.” (W)

“I'm the sort of person, I don't like to feel like I'm taking up people's time [with asking question] […], because, you know, you always feel like they're running late and you're taking up their time and you don't want to take up, the appointment's already a very short amount of time and you don't want to feel like, yeah, you're pushing them back or making them run late or things like that.“ (W)

“No, I feel I can ask anything about, like anything pregnancy related, so I mean that’s why you have those appointments, to ask like if something is happening to your body, is it normal, should you be worried. And, yeah, same for vaccinations so I don’t feel like I, I mean I’ve asked questions about the whooping vaccine, whooping cough vaccines so I’m, yeah, they’re all very approachable.” (W)

Lack of HCP training/ knowledge / specific information to pass on to pregnant women

“I don’t think midwives or GPs were knowledgeable to answer like questions that I had.” (W)

“So it did feel as though we were getting very, you know ‘here's the advice, this is what we've been told to say, there it is, make your own decision’. We still came away from each appointment still craving very much like, well, I still don't know what's best for me.” (W)

I asked, “Look, I know the Covid vaccine is going on, and you know, on the news they really encourage pregnant women to get vaccinated, but is it safe, does it need to be at a specific time during the pregnancy as well, like I’m in my first trimester now, am I better off waiting,” or you know, I had all those questions about the timing, about my specific condition as well because I had a haematoma in the first few weeks, is it going to put any risk in terms of like blood clot, we had those things on the news about is it AstraZeneca were some cases of blood clot were disclosed, so you know, it was a bit stressful whether or not you know, yes in a general way it was recommended, but it’s hard to determine where in my specific case I was better off getting it or not, and if so when, and I found there was not a lot of guidance, yeah. […] but the response I had was very generic, basically the midwife sent me the link of the NHS website saying, you know, which I had already read, you know, saying yes we recommend it.” (W)

“I mean as you know there’s been a lot of back and forth with the Covid vaccines, you know, giving information about that, first of all it wasn’t recommended for pregnant women, then suddenly it was recommended, so it’s where there isn’t much knowledge about it, I think people are less likely to recommend, or they will just skim over it, because if you don’t know much about it yourself then you won’t be able to give the woman the full information, so it’s very important that the health professional themselves, or the person recommending the vaccine has the knowledge to be able to give that information to the woman to make their own decisions, so I think that’s where it was kind of the Covid vaccine was a bit low intake at first. Because we didn’t know about it much for pregnant women and then suddenly it was okay for pregnant women to have it, and then you know, I mean it was with a general public as well isn’t it?” (M)

“I personally feel, from research I’ve done, that there was not enough information to make a proper informed choice for the ladies, especially at the beginning of the pandemic there was so much unknown that I didn’t really think it was ethical for us to be promoting it in the way it was being promoted. Obviously those are my personal views so I didn’t tell that to the ladies but it meant that I was not trying to force it down their throats […] I always approached it with, the advice is to have the vaccine, here’s the information to do your further research and please make up your own mind.” (M)

“I think in terms of the flu vaccination because there is now more long-term sort of safety data then probably you know I’ve had more information about that and especially, and now with whooping cough you know several years on there’s more safety data available. But certainly in, with the Covid

vaccination it felt you know very much like we were giving advice but really the evidence wasn't there for us to be able to say yes this is definitely safe and I can reassure you. And so, I think then that it was more about sort of saying, well this is what the Government advice is, here's the other information, you know if you want any more information, you know maybe speak to the midwives because you know the hospital teams may have more you know information because it's their sort of specialism, rather than us. […] you know quite often actually I'll just go to the practice nurses and say, where would you go for advice?" (GP)

"I think, when you are pregnant you are not just looking after the most precious thing in the world, and so you want to make sure whether whatever you're putting in your body is safe for the baby. I think the issue a lot of like some of the queries, some of them I could deal with, some were just, I don't didn't have the answers, because it's just such a niche area that I don't have the knowledge or expertise to sort of reassure them. I can sort of signpost them, but actually being able to fully reassured them, I can't. [...] *Interviewer:* So where would you sign post them to? *Interviewee:* So either that's the midwife or the NHS websites or help pages. Yeah, or the practice nurse actually, because I some of them are quite good. Some of them are better than us." (GP)

"I think that midwives in particular are not necessarily well versed in being able to properly consent women on all of the side-effects [of vaccines], and all of their risks and benefits, so I don't think that there should be a blanket uniform approach that every single, as with any medication or any procedure, I think that informed consent and choice is really important and if we are not giving them the correct training to answer quite difficult questions that come from some of the patients, they come and ask us quite difficult questions, it leaves us in a very tricky situation to promote something blindly based on what the government, local government kind of policy is, so I think they certainly have a place in maternity and but it needs to be on an individual risk assessment basis, and to have the proper time and counselling to go through what that means for each woman, which we don't have the time to do." (M)

Examples of HCP's commitment to relay relevant information and have honest dialogue

"I tend to say, we have a poster up in the clinic actually which shows a piece of research that was done on I think 250,000 women and that showed us that the Covid-19 vaccine is safe and that actually getting Covid in pregnancy can pose significant risks. I think it doubles the risk of pre-term birth and also doubles the risk of stillbirth. So, you know that, from the research that's been done, it is considered safe and that getting Covid in pregnancy is risky. That sort of tends to be how I phrase it. […] I've had some people say, "Oh okay, I didn't know that." I had someone on the spot decide actually they did want to be vaccinated. So I think having statistics and infographics to show the counter risk of not being vaccinated can be effective in encouraging women to have the vaccine. But equally some people look at it and say, "Yeah still it's not, I'm not, it's still too new and I don't want to have it done." (M, Note: the poster actually targeted HCPs, but it was put up in the clinic)

"[*In response to queries regarding Covid vaccines in pregnancy*] I've sent a lot of people, you know, links to websites of the Royal College of Midwives and Gynaecologists websites, I give a lot of links to that, there's lots of up to date information there…" (O)

"That's long conversation, trying to change an anti-Covid, anti-vaccine person […] They always think about numbers and I said, I try to be on their side and it's true they are very few numbers, risk is minimal but it happens and it's true and it's happens and you don't want it to happen to your baby. And then it's when they get like a bit, like you can see that something opens in their mind and it's a bit like more information, get more, please read more, think about it, and then you can see that you open a bridge there and then like "okay". I think just getting that okay and just getting on their minds

"okay, I think about it", that's just huge thrill. When we are talking about anti-vaccines people that's huge but that takes ages." (M)

"I mean I often ask them you know, what's your reasons for not wanting the vaccines, is there anything I can help you to understand, is there anything you've heard, you know can I dispel any rumours or any things that you've heard? So yeah, we have to really explore it and as long as they understand you know the risks then they've made an informed decision. […] I mean I have had some women, birthing people that have changed their minds and I've had some that have come in and they've said no, I don't want it, from booking they're absolutely adamant they don't want it, they understand, they know the risks, they've made that informed decision. But every now and again yes, you are able to, with further information and conversation, how it's delivered I think, it's how it's delivered, isn't it? It's how it's related to that person so that they can understand, oh actually this is what I thought about a vaccine but actually it's not true. For example, lots and lots of our service users didn't think that it was safe to have the vaccine in pregnancy but we know that it is so, but a lot of that was mainly reassurance and by talking to them and having an honest conversation with them and they're able to make the decision that it is safe." (M)

"if I've not seen them before I don't know what other midwives are saying to them so I can't guarantee that they've been told all the information. […] to the women I'd sort of say, we're not trying to force you into this at all but I just want to make sure you have got the information to make that decision, can I just tell you about it again?" (M)

Lack of research evidence

"…let's err on the side of caution, because you know there's not a lot of studies or research done with pregnant women, breastfeeding, breastfeeding as well, it sucks, so if you read the, for any medication it says nah don't take it during pregnancy, don't take it, because it's just not worth for the pharmaceutical companies and whatever to pay for study, it's sometimes not ethical as well to do studies with pregnant and breastfeeding women, so we are just left out of science." (W)

"People have been complaining they're having blood clots, different side effects, and I ask, have they had a study with pregnant women that have taken it, they're like no. So why would I take something that they're not sure? So are you trying to use me for a test?" (W)

"It's a bit, people were a bit sceptical about the speed at which the vaccine was created really." (M)

"A lot of people will have a vaccination but they don't want to be vaccinated in pregnancy, they'd say I'm going to wait until after the baby's born. Their reasoning tends to be that there's not enough evidence to show that it's safe." (M)

"my baby has Ashkenazi like heritage and there's all sorts of like blood conditions, people who are of black origin there are all sorts of different, I mean […] things like sickle cell anaemia only like applies to black people. So research that's based on largely white people isn't going to convince like …" (W)

Language skills

"Because you know, the reason why I said no then, I don't really understand the language then, for my first child. I don't understand the language. But for my second child, I was able to understand the language so I have to take it [*the flu vaccine*]. When they said it's a risk for me that like, it's to protect me and also to protect my child. So that was why I said OK, I will go ahead with the vaccine. But the first one, language barrier." (W)

"and also you know for a lot of my patients they don't really understand or maybe English isn't their first language and you know it's difficult for them" (GP)

"have something called [*name of telephone interpretation service*] so we just phone a virtual interpreter there and then. I would say probably out of, you know, out of a week you would do 5 of those initial booking appointments and at least once or twice a week you would be calling for an interpreter. [...] and then that potentially is putting extra pressure on, you know, if you already have quite a short time for that appointment and you then have to, you say it, interpreter says it, person says it, interpreter says it, you know, it's adding time to it, so maybe that gets rid of some of the nuance of the conversation you might be having." (M)

"I think language barriers can be a bit difficult as well, if you've got an interpreter you're doubling the amount of time because everything you say and everything the patient says is repeated via the interpreter […] We have like hospital-approved leaflets in different languages, I think French, Spanish, Portuguese and Arabic for vaccines so if they come to us and they don't speak any English we can sort of show them the vaccine and sort of say, you know are you happy, yes, no? […] But there's obviously quite a lot of languages that we don't have those leaflets for[…] there is a telephone service that we can phone from the hospital which works quite well but can take really quite a long time to, you know sometimes you're waiting like 15/20 minutes." (M)

"the area that I'm leading now, we have a high number of language barriers that impact on our, the care that we give so we, for example a large number of our ladies are from the black and Asian communities, we have a large number of ladies from the Eastern European communities […] And when I say language barrier, even English, white British women who have social concerns, you need to make sure the language that you use is also just as important as the ones that don't speak any English, if you don't understand what I'm trying to say… […] I also find in my experience that in the UK some of the vaccines that we offer may not be as available or readily so in other countries so they will question you know, why do I need it? For example, so then it's about going really back to the importance of the vaccine, sometimes the interpreters might not understand what the vaccine is themselves, so that can also be quite difficult sometimes but yeah, we, I feel that a large number of, I mean I've just been covering a lot, lots of clinics recently and I feel a lot of these ladies are really proactive once you explain to them the benefits of the vaccine, they are proactive in having it. (M)

For quotes regarding digital skills please see 'Health information system and apps' section above (under 'Information for participants via hardcopy material versus app') and 'Participant recommendations' section below (under 'Include hardcopy information material to avoid digital exclusion')

Emotions and Trust

Fear of vaccine

"Anxiety around the needles, anxiety around, you know, what you're putting inside of your body, what the impact is going to be on the baby, how it's going to impact them, whether or not, you know, there's maybe any like side-effects and stuff like that, those kind of anxieties. … Because when you get told to go and take the whooping cough vaccination, you don't get told whether or not it's going to have any side-effects, you don't get told whether there's a 1 in 100 chance that your baby's going to have six fingers and six toes, and you don't get told any of that, you just kind of get told to go and do your whooping cough injection at this time" (W)

"Obviously in the back of my mind I'm just always thinking like "oh I just hope that these things are doing more good than harm", so it's just because, you know, you read different articles saying it leads to different disabilities for the baby so obviously in the back of your mind you're just worried, like you just hope it's not that and yeah, you just hope that it's not that, any chemicals or whatever, medication that they're giving you that is causing all of that." (W)

"So I said there's a mark there, so and I'm thinking if I produce that or something. … Yeah, in arm, and the shoulder, and the shoulder. […] No, maybe the injection affected to [anonymised baby], because when I see something is not, no-one has any in your family the marks and everything, yeah. […] *Interviewer:* So you think that maybe because you took the flu vaccine when you were pregnant he/her [anonymised baby], could have had that mark? *Interviewee:* Yeah, yeah, because in my country they don't have a vaccination." (W)

"I am in kind of an online group because I had a lot of period pain, to try and help sort out period pains, but most of the people in the group are in there for getting pregnant, and there are a lot of women who are not getting Covid vaccines because they're struggling to get pregnant and they don't want anything to interfere with their menstrual cycles, which there are a lot of records with it, interfering with it. So there's definitely a lot of people wanting to get pregnant who are avoiding Covid vaccines specifically. […] that's led by a dietician, and she's very passionate against that [Covid vaccines] […] that dietician group I'm in, there's people actively telling people not to get the Covid vaccine, that it will destroy their fertility" (W, FGD)

Fear of vaccine-preventable disease

"Well, I remember one of the midwives […] she was like, oh, there's also the whooping cough, you should consider it, you can do whatever you want, but you should consider it, here's the pamphlet, and then she said, I like to suggest it, […], because, I don't know, two weeks ago someone died, like a baby died, and I was like shocked, so I was like, okay, fine, I'll go get it." (W)

Fear of both, vaccine and vaccine-preventable disease

Interviewer: And then what did you think when they told you that *[why Covid-19 vaccines were advised in pregnancy]? Interviewee:* How I became really scared. *Interviewer:* Scared of what, of Covid or the vaccine? *Interviewee:* Yeah, like of Covid, of Covid. So I start taking precautions, like I don't go out unnecessarily, except I have an appointment in hospital, I wash my hands, I cover my, I use a nose mask, I take lot of precautions that okay, I don't want to contract the virus." (W, *declined Covid-19 vaccine*)

Trust in healthcare system and healthcare professionals

"After I trust here too much because in my country they make a different type of vaccine and people not satisfied but here I completely trust them and I did for my health." (W)

"Yeah, no, she [her mother] trusts the healthcare system. I trust the healthcare system but I'm also someone who would try to do more research if they were to be like "oh you should take this". I wouldn't just be, you know, it's the word gullible but just be like yes straightaway. […] I did my own research, so I would go and look for like blogs, like real-life experiences from woman who've taken it…" (W)

"I'm just, if there is a vaccine available I just trust, I trust the medical world that it's for my own good and I will take it, so that's my premise, so I wasn't overly bothered about vaccine in general, unless it's

a very new and recent vaccine such as Covid then I would like to have more information, but for the other ones I wasn't very bothered about getting them." (W)

Reduced trust and/or confusion due to change of guidance

"I think the guidance changed from when it initially came out. I know I had my first dose December 2020, so like as early as I could and they spent ages sort of saying, no chance you could be pregnant, not planning a pregnancy in the next 3 months, not breastfeeding, you know, it was really and then very quickly it changed to being we are actively recommending this for pregnant people and I think people are maybe a bit mistrustful of that, so I think that's quite a factor in not wanting to have the vaccine, the Covid vaccine." (M)

"And also, weirdly, another thing that was playing on my mind is, my sister, she basically was pregnant when Covid first, first came about, like the first proper lockdown, and initially her midwife had called her and said like, you need to get Covid, you need to get the Covid vaccine, we highly, highly recommend it, and she didn't want to get it, but then she was thinking about it overnight, she thought, no, because this is right at the beginning of Covid, no, I should get it done, and then just as she was going to get it done, she then got a call from the midwife again saying, actually, no, we don't recommend it any more." (W)

"I didn't want any (Covid-19 vaccine) during my pregnancy because I had, you know, kind of that initial advice stuck with me, but yeah every appointment I was being told to get the booster, get the booster, but yeah the initial advice was don't get pregnant for three months after your second vaccine." (W, FGD)

"I also found really frustrating that the communication went from the early days of oh we're not sure if you should have it while pregnant, to I guess you probably should have it while pregnant but we're not really going to push it on you, to suddenly it was you must have it, it's really urgent, you're really irresponsible for not having it while pregnant, you're literally putting your baby at risk. The communication was absolutely horrendous, and it went from oh we're not sure, to oh my goodness why haven't you had this six months ago, like at the drop of a hat and I thought that was absolutely appalling." (W, FGD)

Note: Further quotes on this theme in section on 'Access to Covid vaccines within maternity and via vaccination centres' above

Lack of trust and historical events

"a lot of people think about things like the Thalidomide drugs and stuff like that, so I think it makes people uneasy." (M)

"…on social media or sort of if you Googled about the different types of vaccinations. I think there was the Johnson & Johnson one but then there was the case a few years ago about Johnson with regards to it affecting women and cancer I think it was, a few years ago." (W)

"I grew up when the thalidomide crisis happened and that sort of thing so you just think, you know, is it going to have an effect that we don't know or something that will come up in 10, 20 years and then be linked back to this." (M)

"there was this big kind of furore about the MMR, you know, people saying it was linked to autism and then lots of people refusing it for their children and people are like "no, he's been struck off that doctor, it's all rubbish" and, you know, but there's obviously there's still out there a movement of people who are sceptical. There's nothing like that about whooping cough. There is nobody out there saying "ooh be wary of whooping cough vaccine [*which participant accepted*]" (W)

Lack of trust and ethnicity *(all quotes below from people of black or mixed ethnic background)*

"…there was very much a drive for the BAME community must all have this vaccine because they are most susceptible ... but there was very much a huge reservation from many people in the BAME community as to having this vaccine and this was worldwide… You have in America the legacy of them testing many, many vaccines on populations of African Americans without their consent, done in very sinister ways and so you can understand why people from that context, with that history would not want to be the first to jump in the line to have this vaccine, especially when was being pushed for them, you know, you must have it, let's all wait and see but you guys go first so, you know, why." (M)

"a lot of the black ethnic minorities were a bit sceptical about having the Covid vaccine specifically, because of rumours that it was dangerous, it contained this, it contained that, it contains DNA, and they also felt that it was they were being used for experiment" (M)

"I don't want my daughter to be used as an experiment […]" (W)

"I was really upset that day because … when I went, the first midwife I met she called me in, I went in there and she was like, take a seat, so I sat down and then the next thing she said was, do you speak English, and I was like, do I look like someone that doesn't speak English, […] why don't you have a conversation with me to check if I actually speak English or not. […] I was like, I don't want her anymore because, no you can't be treating me like discriminating, because I'm black, I'm covered and you're like, oh maybe she doesn't understand English,…, well no, I've got a BSc, I'm a graduate, I have a degree, I understand and speak English. […] you don't go about asking people and say, do you speak English, it's rude." (W)

"I'm just going to be really blunt now, if I'm speaking to someone and maybe a white person, white British or white European […] I'm not going to really identify with what she's saying because what I know is although we're the same people but what affects the Blacks affects them differently, or if that's not the case it's just I would really be reassured properly if I am speaking to someone who is of the same ethnicity as I am, that being said, if I should go to the health centre and then I've got posters around or someone of a different ethnic background giving me a poster or explaining something to me, that wouldn't, that as well wouldn't let me say 'oh we are not of the same ethnic background, so I'm not going to listen', yes, I would listen but it's it comes, it feels more at home if you have someone who is just like you speaking to you about it. […] Yeah, so if I say it feels like at home, it makes you under… not just understand more but I don't know how to put it, but it's just reassuring, that is what I have realised that it's just reassuring that I'm not alone." (W)

Lack of trust and conspiracy theories /misinformation

"We found in certain ethnic minorities it was very hard to get certain, like sort of black and minority ethnic women, to convince them that the [Covid-19] vaccine was safe, there was a lot of

misinformation going round in those communities … about how the vaccine was dangerous and they were trying to sort of control the population and it could make you infertile and this sort of thing.” (O)

“No, yeah, I said I don’t take [Covid] vaccine because I’m, if they giving the injection probably they are just killing people I said. […] I read on Facebook and it was, I think I still have some doctors who were saying, from Ecuador, and Peru said there is not such a vaccination can stop, it’s just want to kill people. […] Well they said because if they vaccinate they’re going to stop, or they can’t be pregnant again, they’re going to kill the, you know, you produce, [pause], that they’re going to stop having babies.” (W)

“I didn’t want to take the vaccine because I thought maybe Covid was not for me, and because of the myths that the Covid vaccine is here to kill us and what, what, what, I didn’t want to take, but Covid and then, I ended up getting Covid. […] there is a myth that it stopped you from, stopped, I think the female production system. But when you have it, when you are pregnant it will be hard for you, yeah, it will be hard for you to conceive again. […] Yeah, we would be just refusing because of the myths sometimes, you know, and you wouldn’t know why somebody say no I’m not taking it, it’s because of the myths that we hear from outside, you know, yeah.” (W)

“You know, they, all those classic things that have been spreading and the ridiculous media that’s gone on about it. And saying, yeah, you know, […] it’s going to cause damage and insert microchips and what not. We’ve also had people who thought that if they got a Covid swab, then it had like metal wire in it, so that it would catch on the back of their, I’m not entirely sure what she was worrying about. … And thought that that was going to implant something in her throat. Yeah. Sometimes I’m not entirely, I get very confused by their explanations as to, they’re taking concerns and worries and anxieties from about six different unfounded media things. So, yeah, and people who, yeah, it’s that kind of distrust. […] I think, in the media where, well, I’m guessing it’s come from the media. People saying, you know, corona is just a conspiracy for, so that the doctors can get more money. That’s hilarious. [Laughs]” (M)

Emotions and trust linked to pressure and mandatory vaccines

“I think the issue was that the NHS staff themselves were not convinced that the vaccination is appropriate. I think there was quite a lot of politics involved as well, so forcing nurses to get vaccination, then they opposing it, so there was a lot of emotional messaging rather than facts and guidelines, you know, and anyway being a nurse you’re coming from a place of authority, so if you’re using your emotional personal sort of views to convey the message, I think that needs to be addressed because obviously you’re representing a scientific community here in a way and you kind of, you know, this is not optional in my view.” (O)

“I think there is an illusion of freedom and choice that we have and it’s not questioned, so when you have someone who kind of goes, hang on, am I really getting a choice, am I really being given all the information, what are you holding back from me that might impact on the choice I make, I find that quite often when I start to ask those questions I’m very quickly silenced and pushed into the corner because we don’t need that stirring up the waters […]

“I love being a midwife. I hate working for the NHS [*due to pressure regarding mandatory vaccines*]” (M)

Note: See also quotes under ‘Attitude towards mandatory vaccines’ section below

Cultural norms, philosophy and beliefs

Social, cultural norms, including in country of origin/ abroad

"I can feel that sometimes some groups of the ethnicities they, or their culture, their family, they don't want it, I've seen that. […] They are "we don't do it, my family never done it, we don't do it in my family, no, I've never seen it done". They refer to the family which is their culture. That's their comment. That is why it's culture thing." (M)

"it was just after it had become mainstream to have it while pregnant, but I'd heard similarly from a few people that their midwives had been super cautious about it, so to be honest I didn't ask, and it wasn't mentioned to me, because I just knew I wanted to have it. My sister lived in New York and actually this is really bad but a lot of her friends were pretending to be pregnant so they could get the vaccine as a priority in America, and I'd seen you know, that there'd… a lot of women were having it while pregnant in America and had been for quite a long time, so it felt like if there was anything really badly to happen, it felt like there was enough people having it around the world that we would know if there was anything seriously wrong at that point" (W, FGD)

Religion and Covid-19 vaccination

"…with the Covid vaccine there was a lot of cultural sort of issues …, so in the Muslim population things about alcohol and vaccines and pork…" (O)

"we do struggle during Ramadan, women will decline although as far as I'm aware, …they are allowed to have vaccines and pregnant women are exempt from Ramadan, they will sometimes say, "No I don't, during Ramadan I can't have it," and you say, "Oh how many weeks pregnant are you?" and they say, "Oh I'm 17," and you say, "That's fine, when does Ramadan end? Have it after that, you're still in the window"" (M)

"I think that has some impact as well, you know, the fact that I believe that not everything is in our hands, certain things are predetermined but I'm also not to the point where I believe that, you know, let's blame everything on God and the devil…" (M)

"I think I'm in a minority of Catholic people thinking that way […] I suppose as a Catholic death is something that is more accepted than people who are in a way atheist, so my feeling on like death, […] is that people who are, and I saw this a lot in Covid, like Covid pandemic, people who are atheist tend to continually want medical intervention and they don't want to accept that people will die, they want that more and more should always be done to preserve life, especially of the elderly or those with like severe kind of injury or medical condition at all costs, whereas in the Catholic faith it's about the dignity of life and therefore like having a dignified death and therefore accepting that we don't have like the right to choose, that there is like a higher power that may have chosen that that is our time to go." (W)

Other beliefs and philosophy (including letting nature take its course, preference for natural products and homeopathy)

"Humanity is a very interesting, we're a very interesting species and when you look back through history there will always be times of population rise and fall, especially when exposed to a new disease or a new enemy so to speak, warfare or whatever it might be and in some way it's... It's almost like we can try to slow things down but nature will take its course in many ways but also I don't think it will be anywhere near as dramatic as people predict these things to be.

[…] as a child or as a teenager I had many tropical disease, I had malaria, I had typhoid and so on and so forth. In my physical experience, personally, I felt Covid was a lot milder than having something like malaria. I felt that, you know, as most sort of flu. […] a virus has to run its course, you can't medicate against a virus. […] but then it's a bit like chicken pox, you know, there's the whole camp of do we vaccinate our kids against chicken pox or do we let all kids have chicken pox and build up that natural immunity to it. […] I just believe that if we have that sort of natural immunity that builds up within a population it would probably have a better effect than the artificial immunity that we would get, again the whole chicken pox debate." (M)

"I mean like there are natural products you know like teas and lemons, herbs and stuff and I don't think you always have to take medications or vaccinations. … It's not that I'm not going to take it at all, but yeah I was never, I was always a healthy person, I'm not like very sick or stuff, that's why as well. […] No, it's like you know there are so many diseases in the world, like there are people dying from cancer or people are dying from flu every single day. And this one was some other, the Covid is some other disease which okay people will die but it's like a, it happens every single day. … and until now there is nothing to stop cancer or flu you know. Okay, there are some vaccinations as well but, or medications, but it doesn't, if you will get it you either get through it or you will die. I mean like, sorry to hear, sorry to say that but you know like some people they died but it's part of the life I would say, so. I had Covid … I had it 5 days, or one week before I gave birth to my son. And I just, I was, I was totally fine, I just lost, I just lost smell and taste but I was totally fine […] I think it's very mind, it's in your mind, it's in people's minds. You know, like if you think negatively negative things will happen to you. If you think positively positive things will happen to you." (W)

"My view on death is if I'm going to die I'm going to die and, you know…" (W)

"And I'm not an anti-vaxxer in terms of like, if I was travelling I've got all, you know, I'd get my Hep A, Hep B, my rabies, whatever, but that's because if I caught rabies there's no, really, coming back from that, you know, whereas if I caught the flu I might feel awful for two weeks, but don't have the heavy metals in my body and I'm just, yeah. (W)

Homeopathy and antidots against vaccine side effects:

"then I talked to my, I do a lot of homeopathy and so I talked to my homeopath and then she gave me an antidote [*against the whooping cough vaccine*], like a, not an antidote, but like a, yeah […] It's like an, like a, where all of the metals inside the vaccines and, just so they don't feel bad, like when you don't feel bad, so you're not like having a reaction to them, the vaccines. […] I think when you have vaccine sometimes things that flares up your immune system, you get fevers or you feel very bad for one or two days, you know, like the Covid vaccines … my friend was very tired and she was having a fever and everything, so I had an antidote for the Covid vaccine as well and I didn't feel anything after, but I don't remember what was inside this antidote. … But, for example, when he's [*her baby*] going to have his vaccines in a couple, in four weeks, there is an antidote for each of the vaccines that's inside, so like the polio one, all of the side-effects he's not going to get, well, we hope he's not going to get." (W)

Attitudes regarding altruistic reasons for vaccination

"I feel very strongly that you know, we need to take the vaccines for the greater good of everyone." (W)

"Well, for the Covid I thought, I didn't really want to be selfish, so then I was like, you know, for the greater good I didn't want to, I didn't want to put people that I love at risk […] I also, I'm working in a

restaurant, so then there's a lot more people I'm in contact with all the time, so I thought maybe that would also be better for everyone involved. I don't get, I barely get any sick pay anyway, so then I also didn't want to lose out if I did get Covid. […] then if I was ill and then gave it to my other colleagues, then the whole restaurant would have had to close as well." (W)

"Just general people, like my parents or people at work, friends, but, interestingly, I'd say all of them understood me not wanting to get it for the safety of my baby, which is really interesting, because I had a debate at work with someone about this, that they only understood where I was coming from, because they were like, oh, of course, of course, because it's for the safety of my baby, and I was like, well, isn't it interesting, if I said to you, I don't want to get, if I wasn't pregnant, and I said, oh, I don't want to get it, and they said why, and I was like, well, I'm just not sure about the long-term health impact on me, like I don't want it, then you'd have judged me because I was selfish and not doing it for the greater good of society, blah, blah, blah, but why is my health not a valid reason but the health of my baby is a valid reason? " (W)

"unfortunately the situation that one is like under as a like non-double vaxxed person that basically you're selfish, you don't have the right to choose because you could be killing other people. I mean I guess in being pregnant I had more, you know, obvious rights during my pregnancy to say to people "well it's my own, I'm going to put my own baby first" and like choose kind of what to do or not do to try and as it were like keep my baby safe." (W)

Attitude towards mandatory vaccines

Vaccination needed for travel or work

"I have, actually, now got it, but that was because of work, travel." (W)

"Because if I don't have that card […] they're not going let me to fly to my country if I don't have that, or give me a job. Because the Prime Minister said anyone doesn't have that they're not going to work, if they don't have that like identification. […] Yes. You know when you go to the companies for work, and if you don't have a vaccination card they're not going to give you a job. […] No, it was for all the workers, even you can't go, if you don't have that card you can't go to any public restaurant, that was they been already talking about that. […] Yeah, because I need to work, how I will have money if I don't work." (W)

Planned mandatory Covid-19 vaccines for HCP

"80% of people that I knew …had Covid, we'd had it, we'd worked on the frontline the entire time looking after Covid patients with no PPE and no protection, they weren't even wearing masks for about the first four months… So there was a lot of people who just felt like, well, …we didn't need it before and we've either contracted it or we've contracted it twice and we're over it, so why would I now go and have another vaccine to protect myself from it? I think there was a lot of anger towards the Government decision, because it felt like it's a bunch of people who have no idea what's going on on the ground in hospitals who've made this decision, which seemed almost baseless, […], and then to also wait until …less than 48 hours before the final date that they said you could be vaccinated for the first time, to then scrap it. It felt unfair and it felt quite coercive" (M)

"I do not enjoy my job anymore in terms of working for the NHS. […] I feel initially we were thrown out onto the frontline so to speak with no PPE, no idea what was happening and it was just like just

go out there and wing it, you know, there was no vaccine available yet we were there being told just go, just go, just go and then when the vaccine appeared and some of us went, well we've been exposed to this for so long, some of us have had the virus and we've recovered and actually we don't really feel we want the vaccine, we weren't even acknowledged in the sense that we had this view. […] I would have gone there with nothing, if I had a woman who needed to be seen I would have gone there with nothing but it was that fear that if there was something to bring home I was bringing it home to my children, to my husband who didn't deserve that so to speak, so yeah, I put my family on the line for months on end to provide a service to my women and I never complained once about doing that, my family didn't complain about that either and then, so for it suddenly to be a complete… And we had that whole clap for heroes […], I think, if we had managed to get enough of a grassroots movement to make the clapping for heroes evolve into something else to help the NHS it would have been worth it but I feel one minute we were heroes so to speak and a few months later we were the devils who were not going to comply and have this vaccine and I was now endangering all the women I was coming in contact with because I didn't want to have the vaccine, whereas I had seen hundreds of women beforehand and they had no problem with me seeing them even though I hadn't had the vaccine then, so that's, that's the grey area for me, so yeah" (M)

"when I signed the contract I never signed it saying that there was going to be these additional clauses, but and my thing was it was more nobody was listening to my concerns, my genuine concerns about having these allergies, […] I went there and I said, "Look I'm applying for a medical exemption against this, I want to keep my job, I've had Covid, I've got antibodies, I've got severe allergic reactions and I'm not sure that this is right for me, and so I spoke to the GP about getting that and he said he's been told categorically by his practice manager to not sign any medical exemption forms because each medical exemption form that they administer from their surgery they get a fine by whoever, or they don't get funding, they were getting money for getting people vaccinated, and then getting fined if they were giving out any of these… which I just thought was absolutely shocking. […] remember that they put us all at risk with poor PPE and we still had to go to work, things cannot be postponed, so we turned up every day and I didn't have a day off throughout the whole of 2020, yeah, 2020, and you know, to then say look you guys are going to lose your jobs unless you have this was just a deal breaker for me, and it was heart-breaking, because I looked at changing my profession and my career and I was ready to kind of hand in my notice, because it made me really think about do I want to work for a company that is going to take this approach." (M)

"So basically any medical staff that needs to be, has the chance of potentially being in a body cavity needs to have a Hep B vaccine. So that was my thought when everyone was making a fuss about it. They were like, "they can't force it." I'm like, "well, you wouldn't have your job if you hadn't gone and got a Hep B vaccine and got all your virology bloods checked. So, you know, why do you view that as that's just something necessary for your job but this is suddenly something that's being forced on?" (M)

"lots of people sort of resisted all the way though and were prepared to you know give up work so as not to be vaccinated and I think you know, I don't know I'm not really one for societies where everyone has to do the same thing and can be told what to do, I think you should have personal opinion. […] in general practice we were doing lots of telephone calls during the lockdown and then if we were seeing patients in, so we weren't having big waiting rooms full of patients like we do now again because of the risk of them spreading Covid between each other and so you know, say within the practice if there was one doctor who didn't want to be vaccinated and didn't want to do patient-facing work then potentially other GPs could take over. […] you know it's difficult because once you make everyone do something then, I don't know, I guess it would've been the first time probably wouldn't in the UK for a long time that everyone was forced to, doctors were forced to something or not […] *Interviewer:* One of the midwives mentioned that people have to get a Hepatitis B vaccination…

Interviewee: So, it's similar to that, so yeah I don't know why it was, you know that was the way I saw it was sort of in a similar way to that, that actually to work in this job there's certain things that you, you know need to do and you know Hepatitis B is one and maybe Covid was the other. […] I guess with the Hepatitis B it's protecting yourself isn't it from blood-borne viruses, if you get a needlestick injury, and so for that you have to have a booster, we used to have to have a booster every, and once you've had five then you just have an antibody test and if your antibody level is over a certain amount you're protected. And then if it's not, you need a booster basically." […] (GP)

Perceived pressure/ no pressure to get vaccinated

"…especially for those particular women with underlying conditions who were on the fence or who were against the vaccine, they were the ones who more than anything were coming back with the we are feeling bullied and pressured into having this." (M)

"If you feel you're not safe taking it, don't take it, or if you feel you're safe to take it, take it. This country there is nothing like, they'll force you to do things you don't want to do. […] It's good, because we all, we all have our human rights. We're adults, not babies." (W)

"I mean I don't think you should be putting like pressure on people and I appreciate we have campaigns for sexual health where people might spread, I guess that's the only other main thing in our society where we're thinking oh people are spreading things, you know, we have campaigns like that and I'm sure there are people who tell people like, you know, that they must do things not to like risk others. But I haven't had that from a midwife telling me that like I'm selfishly risking other people, you know, fortunately." (W)

Participant recommendations

Clearer provider recommendation, vaccination dialogue and more information (including earlier on, and including in person and/or via digital media)

"I think from what, everything that I've experienced is very much, very brief the information, it just says okay, so you need to have this at 20 weeks, they're just rushing through it a lot of the time so I then have to go back and do my own research so it would be good to spend quite a big chunk of time speaking about what the actual whooping cough is, the risk to your baby and the benefits of having the vaccination." (W)

"they [the midwives] should ask me why is the reason because nobody asked me why didn't you take a vaccine, do you have any problems, do you have a reason? They could speak about this" (W)

[Related quote by HCP: "If people say, "Oh I don't want the Covid vaccine." I never ask them why, once I've given them the information that's fine. And I think people don't, they don't, I don't know if it's fear of judgement or I don't know. But I find a lot of people, they don't really want to talk about vaccine. If they know they're not going to have it, they're not going to attend things to try and encourage them." (M)

“I definitely think it [vaccination] should be discussed with women more, it is in the first appointment but it’s very easy to not do it because we’re giving so much information and we do get quite a few women [at later visit to vaccination clinic] come … and say, oh no, no I don’t know about it, so I think signposting women to leaflets more or that, yeah definitely more information in the first couple of appointments to make, basically make sure they’re getting that information.” (M)

“I think to have a nice conversation, a good conversation with someone who’s knowledgeable and can answer all your questions and your fears, and to not, and to not be judgemental, you know, I, sometimes you ask questions and people will be like, well, it’s to protect your child, but that’s not the question that I asked, I’m asking, you know, is it safe, but instead of just telling me, yes, of course it’s safe, I’d like to know why is it safe and if there’s any effects and, you know, if, yeah.” (W)

“before you have the baby you’re given like a perfect opportunity where people are, you know, doing their research, trying to figure out what the best things, options are, (...), and I think that it’d also be a great time to just add in that there are some vaccinations that you can take once you’re pregnant and these are the benefits for you, because you’re already trying to do that research into how to have the best pregnancy, how to get pregnant, and so you know, I think it’s just like timing as well, just being able to like really utilise what’s available.” (W)

“It would be once you are pregnant but as early on as possible I think would be good, even if you don’t have to take the vaccination until you’re 5 or 6 months it’s still good to sort of prepare for that.” (W)

“I suppose before getting pregnant it’s quite handy to have all the information you need, you can possibly have, it’s always good because we have a bit more time to prepare, and you know, yeah.” (W)

“[there should be more vaccine-related information] in the waiting areas, in national TV, in pictures, like in flyers, yes, by the time you just register for your pregnancy then it becomes part of package of your pregnancy journey.” (W)

“I just think on all vaccines whilst you’re pregnant, there should be lots of information given verbally and in leaflets. Just because I think you just question everything, a lot more so when you're pregnant than if you're getting a vaccine when you’re not.” (W)

“Well if it’s just my GP practice, but I would, I would hope, to be honest it’s complicated to say because I have, it’s very difficult to even speak to a GP there, so, but I feel like they should be doing more in terms of protecting expecting mothers and you know pushing forward because they should be the one that are closest to the, the patients there registered to that practice instead of just coming from you know a vague central NHS source, so if they could, you know, the GPs, they know they should know a woman’s pregnant under their register and they should ask them and say look this vaccine is available go for it.” (W)

“Maybe if they had, um, I know it might be some extra but like maybe classes to kind of like, or drop-in sessions to kind of inform people what they’re getting themselves into or could or couldn’t, you know? (…) it’s just more better to have that interactive session and just hearing it out of, you know, a health professional’s mouth shall I say, yeah, it’s just much better.” (W)

“I think because of Covid vaccination everyone started to think about vaccinations in general and maybe before they haven’t even thought about it, they were just kind of automatic, okay, your kid needs to get vaccinations, you go, you do it and this whole anti-vaxxers movement, you know, it kind of all overlapped each other and maybe there is something that we need to focus on here and promote vaccinations more but based on solid information.” (O)

"there should be digital technologies helpful now, so there should be simple ways to raise queries and maybe allow other people to say "oh that's a good question", you know, and have someone that's quite knowledgeable respond and answer those questions." (W)

"I think the Covid should be more promoted, the Covid, maybe more on TV because you don't see many things on TV like, yeah, there is like, there was discussion and stuff but you don't see much if you don't want to really like interest about the programme, but there should be definitely more and more media about it. (…) So it will be in the evening and yeah, like BBC maybe or something like that. And yeah, they should maybe have as well on these, you know, when you go to hospital and like you said these TV screen there you're watching when you're waiting in waiting, so maybe there should be more advert about the Covid vaccine because I haven't seen any." (W)

"So definitely NHS, with like websites and campaigns and things, GPs, I feel like maybe just more visibility of like adverts on telly, because you don't really, besides baby milk adverts you don't really see many adverts geared at pregnant women or new mothers erm…, and, so, yeah, like adverts, like local pharmacies, like Boots or Lloyds pharmacy, then also just talking to, or having like a come in and talk with us about, you know, vaccines, and we're here to talk to you as well, because especially as you can't always get an appointment with your GP." (W)

Vaccination programmes and messages more targeted to pregnant women

"when you're pregnant you're very much more conscious that you're responsible for someone else, aren't you, you know, it's a difficult time, you know, having a baby, you're more conscious of what you put in your body, you're more conscious of things like pollution and you know, this, that" […] I mean I think sometimes, like I say, the information isn't targeted at pregnant women, it's targeted at everybody isn't it, the pregnant women are a separate class, they feel like they're a separate class but nobody seems to treat them like that when it comes to vaccination they're just, you know, you're pregnant, you'll be fine kind of thing, just get on with it, you know. I think it needs to be more targeted at pregnant women. " (O)

"I think the more information we can get out to women the better and targeted at pregnant women I think, you know, I think they felt they were sort of tacked on the end, pregnant women, it was sort of like saying to everybody, oh and pregnant women as well." (O)

"because you want to make sure that it is safe because it doesn't, it's not just about you, it doesn't just involve you and so if it's targeted towards pregnant women it makes it really focused and it makes it, it allows those questions to be answered. So yeah, I think yeah more focus would definitely help sort of relieve a lot of anxieties for pregnant women." (GP)

"I think the whole communication and management around it [*Covid vaccination*] for pregnant women was awful, we were made a priority in my opinion far too late,…" (W, FGD)

Vaccination programmes, messages and information more personalised

"I still don't feel comfortable in having this blanket approach that everybody must have this, and I think that's just my approach to maternity in general that I don't think one shoe fits every person that there's always going to be risk assessing that needs to be done in order for people to make the right decision. […] because we know that everybody's individual and more maybe frequently asked questions, sheets, or a better way to be able to direct them to a specific person in order to answer or risk assess on a needs basis. And I also think that all pregnant women should be offered an antibody test to check their level of antibodies." (M)

"I had all those questions about the timing, about my specific condition as well because I had a haematoma in the first few weeks, is it going to put any risk in terms of like blood clot, we heard those things on the news about is it AstraZeneca were some cases of blood clot were disclosed, so you know, it was a bit stressful whether or not you know, yes in a general way it was recommended, but it's hard to determine where in my specific case I was better off getting it or not, and if so when, and I found there was not a lot of guidance, yeah." (W)

Provider training

"I think that more education needs to be done for everybody to be able to be sharing the same information, more studies available" (M)

"I think there should be a very strong message from a very, from a very unanimous message coming from the healthcare sector, that the media, and this is all influenced by policy, the message coming forward, and without that I think you will get people that are very, very confused. And then they will go out and seek out information from Professor Google, and they will either come across valid information or fake information, and I think that that is where the complications come into play." (W, OW, FGD) P6

Organisational changes (including opt-out versus opt-in approach)

"it should be an opt out rather than an opt in. […] Meaning that it's standardised, it's in the calendar and you just need to decline it, if you don't want it, but that if not you just go ahead with it. Rather than actually having to proactively go and get it, and discuss it." (W, FGD)

"I like the idea of it almost being an official point in the calendar, like you have your 20 week scan, you have your X week vaccination, you have your, and it's kind of normalised as part of the pregnancy journey rather than this sort of, oh there's this whole separate thing I need to think about and there's data I need to read and research, it's like no it's just very normalised, this is just part of what you do when you're pregnant, at this week you get this." (W, FGD)

"Yeah in that folder, there should be like a leaflet to say, at this week you're meant to have this vaccine, at that week this vaccine, at that, you know, something to show that, okay week-by-week I'd plan ahead and say, oh next week I'm 30 weeks, oh I'm having whooping cough I need to book an appointment, you know, just so you knew what you were doing week-by-week, and the app as well, because the folder can get lost, but the app as well would be very beneficial in the sense that, if I open the app and then it comes up and says, oh whooping cough is due in 30 weeks, yeah, yeah that would make sense." (W)

"I think because of Covid vaccination everyone started to think about vaccinations in general and maybe before they haven't even thought about it, they were just kind of automatic, okay, your kid needs to get vaccinations, you go, you do it and this whole anti-vaxxers movement, you know, it kind of all overlapped each other and maybe there is something that we need to focus on here and promote vaccinations more but based on solid information." (O)

Increased accessibility

"I think it needs to be more accessible around work, because you're already taking quite a lot of appointments off, it was quite difficult to be out of work so much." (W)

"Yeah, I think just making it accessible for in more areas, so yeah, the GP surgeries/clinics works well, the pharmacy works well, and maybe the pharmacies if it's a united effort so it's not that you could go to one pharmacy and have it without no problems but you go into another pharmacy and there's 1,001 questions, you know make it easy and quite streamlined for people would be quite good." (M)

Improved health information systems and apps

"I think it would be good for us all to be on the same page in terms of how we document whether or not women are vaccinated. […] I see them every appointment so I have my system. Women who are seeing different practitioners each time, I might think, oh the person who saw her last discussed it, when actually they didn't and now I'm not discussing it and then it never gets talked about. […] I would make it mandatory. …Because all women should be entitled to getting that information, what they choose to do with that information is up to them. But everyone should have equal opportunity to access that information." (M)

"you're an advocate in your own right so you have your own system of reminding yourself what to discuss with women at each touch point, so you know, some people make themselves a note to discuss this, this, this at that appointment, and I mean you're accountable for your own practice, but I think making it mandatory will generally help, a mandatory documentation." (M)

Cons on mandatory fields:

"I do think, especially being a caseload midwife [for continuity of carer system], you are like building a relationship with the woman, so you don't want it to seem like a checklist all the time [as with mandatory fields that require to check a box to proceed], like if you really want to make a difference, I mean, if you want to like get through to her, maybe it's kind of up to the health professional to understand when would be the best time to discuss it and how to discuss it, rather than someone just reading, have you had vaccinations, and they would be like no, and, you know, I don't know, there's benefits and negatives to both, but I think sometimes having the health, if the health professional knows the woman, then kind of understanding when would be most effective, would be good. […] I think just in, yeah, in general, like if I feel, if the woman is receptive to some things, then maybe I would repeat the vaccine thing again and again, but if I'm already struggling to get through to a woman, then maybe that would be a point where the woman would shut down more if I continued to repeat it. So maybe leaving it to the health professional to try to, it's hard because obviously you want to have a system, but erm…, yeah, I don't know [laughs]." (M)

"the app is just not very good but the idea of having an app is good to have all your information on there, I just think that kind of the user experience could be a lot of better, because I've had to figure it out myself and it's just because I work in digital that I'm able to do it but somebody else might struggle." (W)

Provision of hardcopy information material to avoid digital exclusion

" [better us] a pamphlet, it can just take two seconds to look at it and you've already got all the information, but our phone we still have to log in, you still have to log into that app for you to access that information" (W)

"What should be improved, I think it's giving them, making sure that the women have all the information that is required, so for example given written… because some people… see we've moved away a lot from giving paper information, but to me women still find it useful having handheld information, a lot of things are now electronic, all our leaflets, everything are online, they have to log

onto their [*anonymised maternity app*] in order to access the leaflets, and the information, and if you're not IT savvy they don't always, they're not always able to access those information's, so I think that there is, there is still scope I think for paper information if the woman prefers that method. Because I recall many years ago when we used to give, when the women booked there was a big, big booklet that had all the information about pregnancy, vaccinations, everything, and we used to give it to all first time mothers. And the women found it fascinating, but now a lot of the information they have to go online for it, go on the internet for it, and it just makes it you know, a bit more difficult." (M)

"I do wonder if there, that the paperless model that we are all striving to get to, which is important, maybe has been too much in the maternity setting, I wonder if having a booklet of the ultimate maternity experience, including information about vaccination, that you have to bring along to your appointments like you do with your folder, would be a useful, a more useful way of delivering information. But I appreciate some people feel that's going backwards." (W, FGD)

"*Interviewer*: So thinking about electronic versus hard copy leaflets, what do you think would be more effective? *Interviewee:* I think, to be honest, a hard copy. […] with application you have to actually open it, you have to actually go there and want to read it so it has to be a directed intention whereas with a leaflet that you have at home, it's something that is there, you can just, you know, skim through it but it's there, you can come back to it, someone else can pick it up and read through it, someone can, oh what's this, ah, yeah, yeah, I read it, no, I haven't read it yet, I'm going to read it when I'm in bed. It's kind of a different way that you function with that sort of material." (O)

Addressing language barriers

"I did want to talk about the language barriers in terms of vaccines, especially since I'm, since where I'm working now for me that's been a real obvious barrier I think, but we need to support people to be able to overcome that so they can access vaccines." (M)

"there should be resources out there that, for women to be signposted to immediately, easy access, easy available and this information should also be available in different languages as well. […] Yeah, I don't think they are, they're all English, the majority of them are in English and a lot of the times, even if the Trust wants to transcribe the leaflet into different languages, there's so many things that you have to go through to be able to have it in another language. It needs to be certified, if you know what I mean. " (M)
